# Structure elucidation of female-specific volatiles released by the parasitoid wasp *Trichogramma turkestanica* (Hymenoptera: Trichogrammatidae)

**DOI:** 10.3762/bjoc.10.72

**Published:** 2014-04-02

**Authors:** Armin Tröger, Teris A van Beek, Martinus E Huigens, Isabel M M S Silva, Maarten A Posthumus, Wittko Francke

**Affiliations:** 1 Institute of Organic Chemistry, University of Hamburg, Martin-Luther-King Platz 6, D-20146 Hamburg, Germany; 2 Laboratory of Organic Chemistry, Wageningen University, Dreijenplein 8, 6703 HB Wageningen, The Netherlands; 3 Laboratory of Entomology, Wageningen University, P.O. Box 8031, 6700 EH Wageningen, The Netherlands

**Keywords:** natural products, structure elucidation, sex specific, *Trichogramma turkestanica*, volatile deoxypropionates

## Abstract

Females of the parasitoid wasp *Trichogramma turkestanica* produce the putative polydeoxypropionates (2*E*,4*E*,6*S*,8*S*,10*S*)-4,6,8,10-tetramethyltrideca-2,4-diene and (2*E*,4*E*,6*S*,8*S*,10*S*)-4,6,8,10-tetramethyltrideca-2,4-dien-1-ol or their enantiomers as sex specific volatiles. The structures were assigned on the basis of GC–MS investigations using synthetic reference compounds.

## Introduction

Wasps of the genus *Trichogramma* (Hymenoptera: Trichogrammatidae) are egg parasitoids of insect eggs [1]. They are commercially used as biological control agents against many insect pests, worldwide [2]. The taxonomy of *Trichogramma* wasps is hampered by their particularly small size and by strong morphological similarities between the species. Several attempts, including chemotaxonomic approaches, have been made to solve this problem [3–5]. While it is generally accepted that parasitoid wasps use chemical cues for host location [6], little is known about (volatile) semiochemicals playing a role in intraspecific communication [7–11].

Based on bioassays with *Trichogramma turkestanica* and extensive analytical investigations, two female-specific volatile compounds have been described as possible pheromone components and were tentatively identified as the hydrocarbon 2,6,8,12-tetramethyltrideca-2,4-diene (**A**) and the corresponding allylic alcohol 2,6,8,12-tetramethyltrideca-2,4-diene-1-ol (**B**) [12]. Since the two proposed structures were deduced from analytical data only, the aim of the present study was to scrutinize these suggestions by unambiguous synthesis.

## Results and Discussion

**Figure 1:**
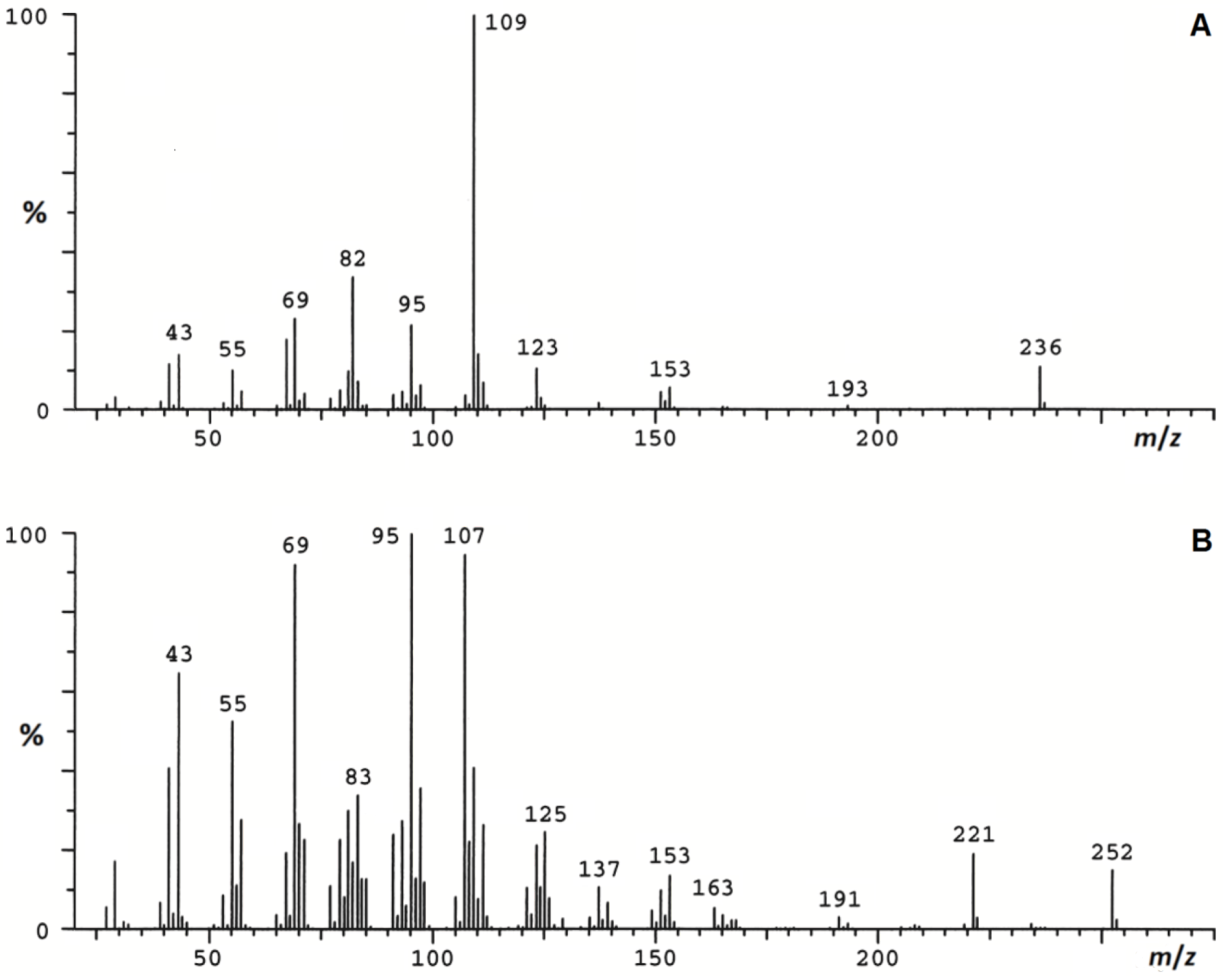
Published 70 eV EI mass spectra of the naturally occurring compounds **A** and **B** [12].

**Figure 2:**
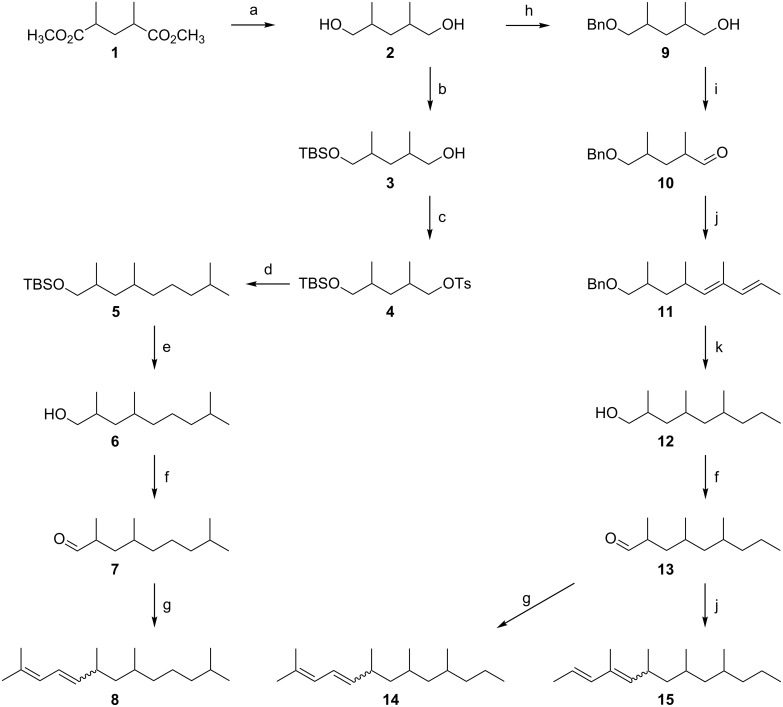
Synthesis of tetramethyltrideca-2,4-dienes **8**, **14** and **15**. Conditions: a: LiAlH_4_, Et_2_O, −30 °C to rt, 12 h; b: 1) NaH, THF, −20 °C to rt 1 h; 2) TBDMSCl (1 equiv), rt, 10 h; c: *p*-TsCl, DMAP, TEA, DCM, −20 °C to rt, 12 h; d: (3-methylbutyl)magnesium bromide, CuI, THF, −90 °C to rt, 16 h; e: TBAF, THF, rt, 14 h; f: PDC, MS 4 Å, DCM, −20 °C, 90 min; g: (3-methylbut-2-en-1-yl)triphenylphosphonium bromide, *n*-BuLi, THF, −40 °C to rt 16 h; h: 1) NaH, THF, −20 °C to rt, 1 h; 2) BnBr (1 equiv), *n*-Bu_4_NI, 0 °C to rt, 16 h; i: PCC, MS 4 Å, DCM, −20°C, 90 min; j: [(*E*)-1-methylbut-2-en-1-yl]triphenylphosphonium bromide, *n*-BuLi, THF, −40 °C, 14 h; k: H_2_, 20 bar, 10% Pd/C, pentane, rt, 18 h.

The 70 eV EI mass spectra of the two natural products **A** and **B** are depicted in Figure 1 [12]. Because the carbon skeleton had been reported to be the same in both compounds*,* we used the hydrocarbon **A** as the first target for the preparation of a reference sample. As an initial approach we aimed at the synthesis of a library of stereoisomers of **A** (Figure 2).

**Figure 3:**
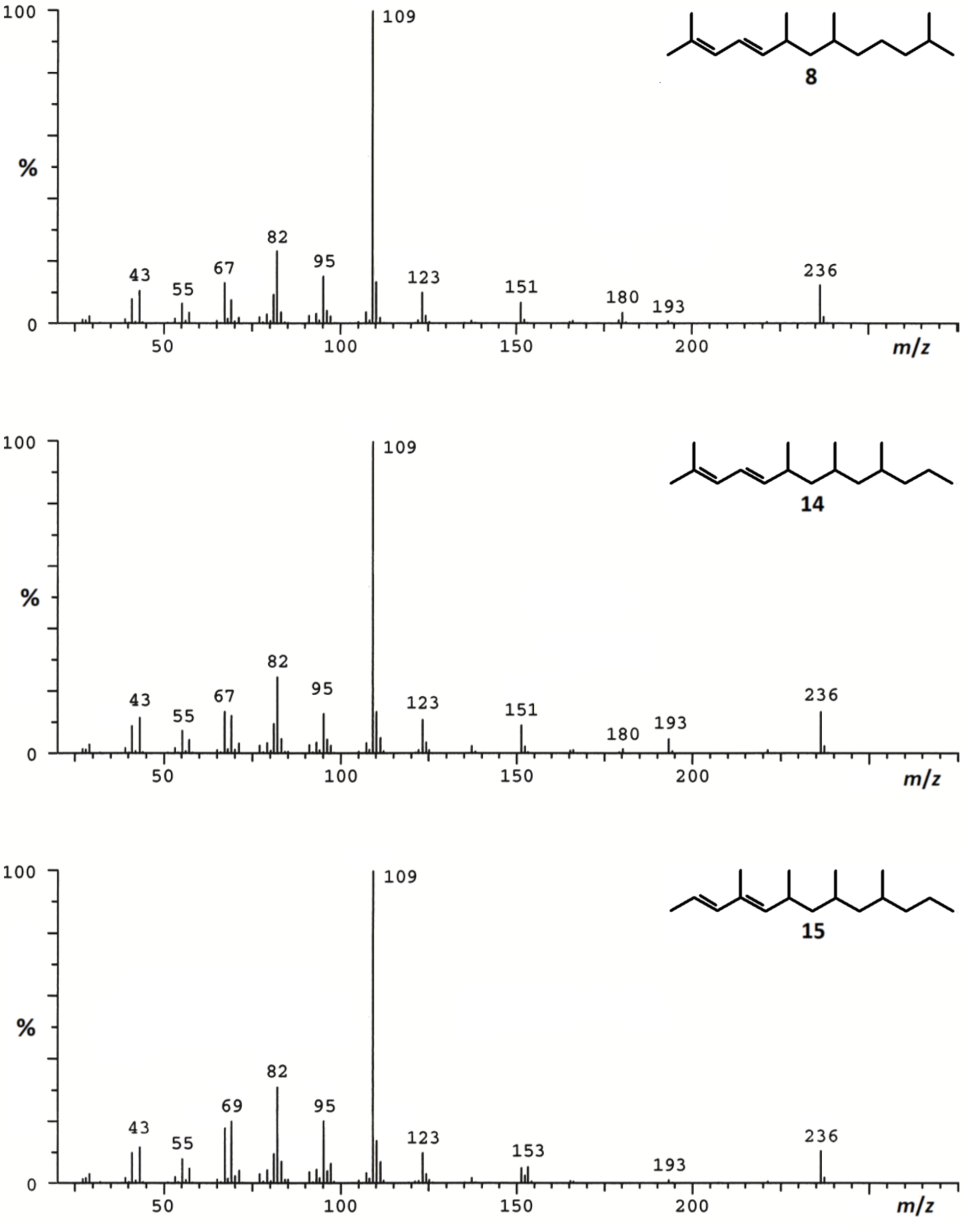
70 eV EIMS of synthetic tetramethyltrideca-2,4-dienes **8**, **14** and **15**. These spectra were run under the same analytical conditions as described in the original paper [12].

The commercially available racemic *anti*-dimethyl 2,4-dimethylglutarate (**1**) was reduced to the corresponding racemate of 2,4-dimethylpentan-1,5-diol (**2**) [13], which was further transformed to the monosilyl ether **3** and subsequently to the corresponding tosylate **4**. Using 3-methylbutylmagnesium bromide and applying cuprate chemistry [14], **4** was chain elongated to **5**, which was deprotected to yield *anti*-2,4,8-trimethylnonanol (**6**). After oxidation to the corresponding aldehyde, the stereogenic center at position 2 underwent ca. 6% epimerization to yield **7** as a mixture of 4 stereoisomers. The synthesis of 2,6,8,12-tetramethyltrideca-2,4-diene (**8**) was completed by Wittig reaction using (3-methylbut-2-en-1-yl)triphenylphosphonium bromide [15]. As expected, the obtained mixture of all possible stereoisomers of **8** showed an *E*/*Z*-ratio of ca. 2:1 and was highly dominated by the *anti*-isomers (see Supporting Information File 1, Figure S1). The *E*/*Z*-isomers of the two pairs of diastereomers could be separated by gas chromatography. Analysis by coupled gas chromatography/mass spectrometry (GC–MS) showed the 70 eV EI mass spectra of the synthetic compounds to be almost identical. Though the mass spectra of **8** (Figure 3) and that of the natural product **A** (Figure 1) showed strong similarities, small but decisive qualitative and quantitative differences in the fragmentation pattern were obvious. In contrast to the mass spectrum of **A**, those of **8** showed an additional signal at *m*/*z* 180 (loss of isobutene), whereas the signal at *m*/*z* 153, present in the spectrum of **A** was absent. In addition, the pair at *m*/*z* 67 and *m*/*z* 69 showed different relative abundances.

Thus, it was obvious that the structures published for the natural products needed modification. Sticking to the basic carbon skeleton proposed for **A** and **B** [12], we looked for a plausible biogenetic way providing an alternative structure for **A** from reasonable building blocks without too much changing of **8**. Methyl branching at positions 6 and 8 was indicated by pronounced signals at *m*/*z* 123 = M^+^ − 113 and *m*/*z* 151 = M^+^ − 85 in the mass spectrum of **A** [12] (Figure 1), which we regarded to result from loss of the last eight or six carbons, respectively. Therefore, we kept this feature in the structure of the next candidate. Leaving the dienic system unchanged, we consequently decided to shift the methyl group from position 12 in **8** to position 10. As a result, our next candidate was 2,6,8,10-tetramethyltrideca-2,4-diene (**14**), because the formation of such a polydeoxypropionate by condensation of four units of methylmalonate, followed by chain elongation with malonate and another methylmalonate (including deoxygenation steps) seemed to be plausible [16]. These biogenetic considerations excluded methyl groups at positions 9 and 11.

To prepare a mixture of all stereoisomers of **14**, we again used **2** as the starting material (Figure 2). Monobenzylation to **9**, followed by oxidation, yielded the aldehyde **10**, which was chain elongated by Wittig reaction with [(2*E*)-1-methylbut-2-en-1-yl]triphenylphosphonium bromide [17] to afford the protected dienol **11** as a mixture of 4 racemates. This was due to partial epimerization at position 2 of the aldehyde **10** and the less selective Wittig coupling. Consequently, hydrogenation of **11** yielded a mixture of all 8 possible stereoisomers of the saturated alcohol **12**; for relative proportions, see Supporting Information File 1. Starting from **12** and following the sequence described for the transformation of **6** to **8**, a mixture of the stereoisomers of the target diene **14** was obtained via 2,4,6-trimethylnonanal (**13**) [15]. GC–MS-analysis of this mixture revealed a 3:1 dominance of the *E*-configurated racemates over the earlier eluting *Z*-series. The chromatogram showed seven well-separated peaks, indicating that only two isomers co-eluted (see Supporting Information File 1, Figure S1). However, it soon became clear that the natural product was not among the synthetic compounds **14**, because their mass spectra basically showed the same differences to that of **A** (presence of *m*/*z* 180, absence of *m*/*z* 153) as did the 2,6,8,12-tetramethyltrideca-2,4-dienes (**8**) (Figure 1 and Figure 3). In the next approach we considered a structural change in the dienic system of **14** as an additional modification of the published structure of **A**.

As indicated above, the formation of the signal at *m*/*z* 180 in the mass spectra of **8** and **14** may be due to the loss of isobutene – which would be avoided by a shift of the methyl group from carbon 2 to positions 4 or 5. Results of derivatization reactions of the natural product by Diels–Alder reaction with 4-methyl-1,2,4-triazolin-3,5-dione left room for the positioning of a methyl group at carbons 2, 3, 4, or 5 of the chain [12]. Location at position 2 would provide **14**, which was already ruled out. According to a conceivable biosynthesis of the natural product from methylmalonate and acetate, positions 3 and 5 would be less favourable [16]. However, linear condensation of five methylmalonate units and a final malonate (and again corresponding deoxygenation steps) would produce the carbon skeleton of a 4,6,8,10-tetramethyltrideca-2,4-diene (**15**). Thus, from a biosynthetic point of view, the structure would be reasonable and follow established principles.

To test the validity of our assumption, we transformed **13** into **15** by Wittig reaction using [(*E*)-1-methylbut-2-en-1-yl]triphenylphosphonium bromide [17] in the chain elongation step (Figure 2). The reaction yielded a mixture of products representing two quantitatively different series of eight racemic stereoisomers of **15**. According to the *E*-configuration of the double bond in the starting phosphorane and the known *E*-selectivity of the reaction [17], we assigned the (2*E*,4*E*)-configuration to the more abundant and later eluting series of stereoisomers and the (2*E*,4*Z*)-configuration to the less abundant one. The diastereomers could be well separated by GC, and only two members of the less abundant 4*Z*-series co-eluted (see Supporting Information File 1, Figure S1). The mass spectra of **15** were the same as that of the natural product (Figure 1 and Figure 3). Gas chromatographic properties on a non-polar DB-1 and a polar Stabilwax (for conditions see ref. [12]) of the first eluting racemate of the more abundant 4*E*-series matched those of the natural product, strongly indicating (2*E*,4*E*)-configuration of the conjugated double system in the target compound (see Supporting Information File 1, Figure S2).

After the gross structure of the natural product had been found to very likely be (2*E*,4*E*)-4,6,8,10-tetramethyltrideca-2,4-diene (**15**), we had to assign the relative stereochemistry at the three stereogenic centers. Actually, we had to decide between 4 structures: *syn*,*syn*-, *syn*,*anti*-, *anti*,*syn*-, and *anti*,*anti*-configuration of the methyl groups at carbons 6, 8, and 10. Since semiochemicals representing saturated polydeoxypropionates typically show *syn*-configuration, suggesting general principles in enzymatic chain elongation [16,18], we hypothesized (2*E*,4*E*,6*S*,8*S*,10*S*)-tetramethyltrideca-2,4-diene or its enantiomer to be the natural product.

**Figure 4:**
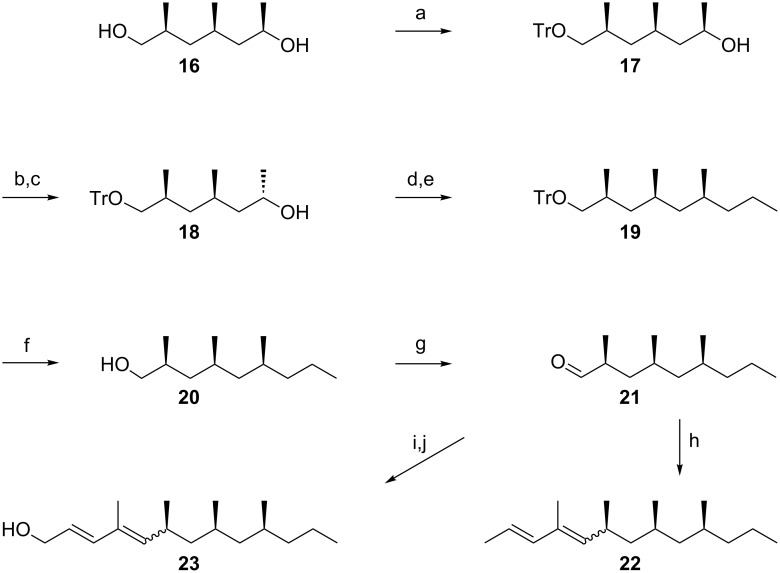
Synthesis of (2*E*,4*EZ*)-*syn*,*syn*-4,6,8,10-tetramethyltrideca-2,4-diene (**22**), as well as (2*E*,4*E*)- and (2*E*,4*Z*)- *syn*,*syn*-4,6,8,10-tetramethyltrideca-2,4-dien-1-ol (**23** and **23a**). Conditions: a: TrCl, DMAP, TEA, DCM, rt, 16 h; b: 1) DIAD, TPP, THF, −20 °C, 30 min; 2) 3,5-DNBA, rt, 40 min; 3) **17**, rt, 17 h; c: 2 N NaOH aq, THF/MeOH, rt, 45 min; d: MsCl, TEA, DCM, −80 °C, 2 h; e: *n*-propylmagnesium bromide, CuI, THF, −90 °C to rt, 48 h; f: *p*-TsOH, MeOH/THF/H_2_O, rt, 2 h; g: PDC, MS 4 Å, DCM, −20 °C, 90 min; h: [(*E*)-1-methylbut-2-en-1-yl]triphenylphosphonium bromide, *n*-BuLi, LiBr, THF, −40 °C, 12 h; i: 1) diethyl [(1/2*EZ*)-3-(methoxycarbonyl)-1-methylprop-1/2-en-1-yl]phosphonate (as a mixture of isomers [23]), NaHMDS, LiBr, THF, −90 °C, 1 h; 2) **21**, −70 °C to rt, 16 h; j: DIBAlH, DCM, −80 °C to rt, 2.5 h.

To establish a *syn*,*syn*-configuration of the three chiral centers in our target compound, we followed the way used by Mori and Kuwahara for the synthesis of the mite pheromone lardolure [19], which we had successfully adopted in the synthesis of vittatalactone [20]. Commercially available 2,4,6-trimethylphenol was hydrogenated to form all-*cis*-2,4,6-trimethylcyclohexanone. Subsequent Baeyer–Villiger oxidation, followed by reduction of the obtained lactone, yielded *syn*,*syn*-2,4-dimethylheptan-1,6-diol **16** (Figure 4). Protection of the primary hydroxy group gave **17** followed by a Mitsunobu sequence involving the secondary hydroxy group, afforded **18**. Chain elongation of **18** by applying cuprate chemistry [14] to its mesylate gave **19** which was deprotected to give **20**, which, in turn was oxidized to *syn*,*syn*-2,4,6-trimethylnonanal (**21**). As described for the transformation of **13** into **15** (Figure 2), **21** was chain-elongated to a 1:3-mixture of (2*E*,4*Z*)-*syn*,*syn*- and (2*E*,4*E*)-*syn*,*syn*-4,6,8,10-tetramethyltrideca-2,4-diene (**22**). The stereochemical composition of this defined mixture of stereoisomers of **15** (see Supporting Information File 1, Figure S2) was assigned by NMR spectroscopy, matching respective data reported in the literature [21–22].

The mass spectrum of (2*E*,4*E*)-*syn*,*syn*-4,6,8,10-tetramethyltrideca-2,4-diene (Figure 1 and Figure 3) and its gas-chromatographic properties perfectly matched the corresponding data of the natural product **A** (linear retention index on DB1 = 1511 and on Stabilwax = 1579) [12]. The structure closely resembles those of the oligomethylpolyenes known as pheromones of nitidulid beetles [24]. The 4-methyl-2*E*,4*E*-diene motif has been known as a substructure of pheromones of beetles, cockroaches and scale insects [16]. Whether these compounds are insect-produced or products of (endo)symbionts will need further investigations.

In analogy to the structure of **A**, compound **B** was assigned to be (2*E*,4*E*,6*S*,8*S*,10*S*)-4,6,8,10-tetramethyltrideca-2,4-dien-1-ol (**23**) or its enantiomer. According to the procedure described by Markiewicz et al. [23], racemic **23** was prepared by vinylogous Horner–Wadsworth–Emmons reaction of the aldehyde **21** with the anion of diethyl [(2*E*)-3-methoxycarbonyl-1-methyl-2-en-1-yl]phosphonate, followed by reduction of the resulting methyl ester (Figure 4). The obtained product proved to be a mixture of **23** and its (2*E*,4*Z*)-isomer (**23a**) in a ratio of about 1:1. For an unambiguous gas-chromatographic discrimination of **23** and **23a** we also synthesized a mixture of the two series of stereoisomers by applying “salt-free” conditions. As expected, the blend was strongly biased by the (*Z*)-series **23a** (see Supporting Information File 1, Figure S3A). The mass spectrum of the (2*E*,4*E*)-configured 2,4-dien-1-ol **23** (Figure 1), and its gas-chromatographic properties perfectly matched the corresponding data of the natural product **B** (linear retention index on DB1 = 1766 and a Stabilwax = 2317) [12].

Since the preparation of enantiomerically pure *syn*,*syn*-2,4,6-trimethylnonanal has been described [25–28], our approach includes a formal synthesis of optically active **A** and **B**. Until now, we did not find suitable conditions to separate the enantiomers of the tetramethyldiene **22** and the respective allyl alcohol **23** (or corresponding derivatives thereof) in order to assign the absolute configuration of the natural products by enantioselective gas chromatography. However, ozonolysis of a natural sample [29] containing the two compounds, followed by reductive work-up, would yield *syn*,*syn*-2,4,6-trimethylnonanol, the enantiomers of which can be easily separated by using heptakis-[2,3-di-*O*-methyl-6-*O*-(*tert*-butyldimethylsilyl)]-β-cyclodextrin (50% in OV1701) as the stationary GC phase, operated at 100 °C. Under these conditions, the (2*S*,4*S*,6*S*)-enantiomer [30] is the later eluting stereoisomer, giving an α-value of (*t*r_2_:*t*r_1_) = 1.019 (see Supporting Information File 1, Figure S4).

As a result of our investigations we corrected the structures of the initially published *Trichogramma* compounds and showed a way to determine the enantiomeric composition of the natural products. Details including the assignment of the absolute configuration of the compounds will be published separately. As yet, the biological significance of the two female-produced compounds is unclear, however, Pompanon et al. [31] showed the presence of a sex specific pheromone released by females of *Trichogramma brassicae*. Future bioassays with the two new polydeoxypropionates will scrutinize whether they induce courtship behavior in males of *Trichogramma turkestanica*, as did the extract of females [12].

## Supporting Information

File 1 Experimental details and characterization data for synthesized compounds.
