# Supplementary material for: Structure elucidation of female-specific volatiles released by the parasitoid wasp Trichogramma turkestanica (Hymenoptera: Trichogrammatidae)
Source: Beilstein J Org Chem. 2014 Apr 2;10:767–73. doi: 10.3762/bjoc.10.72 (PMC3999836; doi:10.3762/bjoc.10.72)
Supplement: File 1 — Experimental details and characterization data for synthesized compounds. [file Beilstein_J_Org_Chem-10-767-s001.pdf]

Supporting Information

for

Structure elucidation of female-specific volatiles released by the parasitoid wasp *Trichogramma turkestanica* (Hymenoptera: Trichogrammatidae)

Armin Tröger[1], Teris A. van Beek[2], Martinus E. Huigens[3], Isabel M.M.S. Silva[3], Maarten A. Posthumus[2], and Wittko Francke[1 *]

Address: [1]Institute of Organic Chemistry, University of Hamburg, Martin-Luther-King Platz 6, D-20146 Hamburg, Germany, [2]Laboratory of Organic Chemistry, Wageningen University, Dreijenplein 8, 6703 HB Wageningen, The Netherlands and [3]Laboratory of Entomology, Wageningen University, P.O. Box 8031, 6700 EH Wageningen, The Netherlands

Email: Wittko Francke - francke@chemie.uni-hamburg.de
*Corresponding author

Experimental details and characterization data for synthesized compounds

Table of contents

1. General information S2
2. Syntheses and analytical data S3
3. Gas chromatograms S28
4. ^{1}H and ^{13}C NMR spectra S32

General Information

All reactions were carried out under an argon atmosphere in flame-dried glassware with magnetic stirring. Dichloromethane was dried by distillation over calcium hydride, THF was dried by distillation over NaK. All reagents were purchased from Sigma–Aldrich Chemie GmbH (Munich, Germany) and Merck KGaA (Darmstadt, Germany) and were used without further purification. Reaction products were purified by column chromatography on Silica 60 (40–63 µm mesh, Merck) under 0.5 bar pressure. TLC analysis was performed on Merck TLC aluminium sheets Silica gel 60 F_{254} using phosphomolybdic acid 10% in ethanol as the visualization reagent.

^{1}H NMR spectra, proton-decoupled ^{13}C NMR spectra, and 2D H,H-COSY, HSQC and HMBC experiments were recorded on Bruker instruments AV400 and DRX500 (Bruker Biospin GmbH, Rheinstetten, Germany) of the field strength indicated in the experimentals at 25 °C. $CDCl_3$ served as the internal standard (7.26 ppm and 77.16 ppm, respectively). Data are reported as follows: δ in ppm (s = singlet, d = doublet, t = triplet, q = quartet, m = multiplet; integration; coupling constant(s) *J* in Hz).

Gas chromatography coupled with mass spectrometry (GC–MS) at low resolution was carried out by using a Fisons Instruments NV (Manchester, UK) GC 8060 linked to a Fisons Instrument mass spectrometer MD 800. Helium served as the carrier gas. Separations were achieved with a fused silica capillary DB-5ms (30 m, 0.25 mm i.d., 0.25 µm film, Agilent GmbH, Böblingen, Germany), run for 3 min at 50 °C then programmed to 300 °C at a rate of 10 °C/min.

Enantioselective gas chromatography was carried out by using a Fisons Instrument AS GC800 equipped with an FID. Hydrogen served as the carrier gas, and separations were achieved at 100 °C with a homemade fused silica capillary coated with heptakis-[2,3-di-*O*-methyl-6-*O*-(*tert*-butyldimethylsilyl)]-β-cyclodextrin (50% in OV-1701, 25 m, 0.25 mm i.d., 0.25 µm film) as the stationary phase, applying temperature programs indicated in the experimentals.

Syntheses and analytical data

***anti*-5-(*tert*-Butyldimethylsilyloxy)-2,4-dimethylpentan-1-ol (3)**

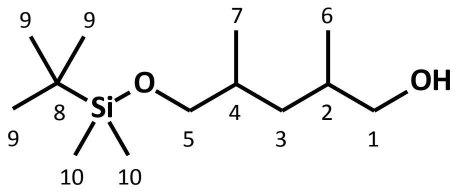

To a suspension of 341 mg (14.20 mmol) of sodium hydride in 30 mL dry THF a solution of 1.874 g (14.18 mmol) *anti*-2,4-dimethylpentan-1,5-diol in 10 mL dry THF was added dropswise at −20 °C. The mixture was warmed to room temperature and stirred until the formation of hydrogen ceased. Subsequently, the mixture was cooled to 0 °C, and a solution of 2.130 g (14.13 mmol) TBDMSCl in 4 mL dry THF were added slowly. After stirring over night at room temperature, the solvent was removed in vacuo, and the residue was partitioned between 40 mL diethyl ether and 10 mL saturated aqueous sodium hydrogencarbonate solution. The organic layer was separated and washed with two 10 mL portions of hydrogencarbonate solution and brine. After drying with anhydrous magnesium sulfate, the solvent was removed in vacuo. Column chromatography on silica (hexane:ethyl acetate = 10:1 to 2:1) yielded 2.960 g (12.01 mmol, 85%) of **3** as a colorless oil.

^{1}H-NMR, ^{1}H-^{1}H-COSY, HSQC, HMBC (400 MHz, $CDCl_3$): δ [ppm] =0.04 (s, 6H, 2× C10), 0.84 (d, $^3J_{H\text{-}H}$=6.8 Hz, 3H, CH_3 C6), 0.87 (d, $^3J_{H\text{-}H}$=7.1 Hz, 3H, CH_3 C7), 0.89 (s, 9H, 3×CH_3 C9), 1.13/1.21 (2ddd, $^2J_{H\text{-}H}$=13.64, $^3J_{H\text{-}H}$=9.1/9.1, 4.8/4.3 Hz, 2H, CH_2 C3), 1.69-1.81 (m, 2H, 2×CH C2 C4), 3.39/3.42 (2dd, $^2J_{H\text{-}H}$=9.9, $^3J_{H\text{-}H}$=6.3/6.3 Hz, 2H, CH_2 C5), 3.46/3.49 (2dd, $^2J_{H\text{-}H}$=10.4, $^3J_{H\text{-}H}$=6.3/6.3 Hz, 2H, CH_2 C1).

^{13}C-NMR, HSQC, HMBC (101 MHz, $CDCl_3$): δ [ppm] = -5.20 (q, C10), 16.59/16.77 (2q, C6 C7), 18.50 (s, C8), 26.10 (q, C9), 33.08/33.23 (2d, C2 C4), 36.98 (t, C3), 69.14/69.17 (2t, C1 C5).

MS (EI, 70 eV, GC/MS DB-5ms 30 m): m/z (%) = 39 (3), 40 (1), 41 (10), 42 (2), 43 (7), 44 (1), 45 (4), 47 (3), 53 (1), 55 (100), 56 (6), 57 (7), 58 (4), 59 (8), 60 (1), 61 (3), 67 (1), 69 (18), 70 (1), 71 (1), 72 (1), 73 (24), 74 (4), 75 (78), 76 (6), 77 (5), 79 (1), 81 (1), 83 (3), 85 (2), 86 (1), 87 (1), 88 (2), 89 (8), 90 (1), 91 (1), 93 (4), 95 (1), 97 (90), 98 (7), 99 (3), 100 (1), 101 (3), 105 (22), 106 (2), 107 (1), 113 (1), 115 (7), 116 (1), 117 (1), 119 (2), 129 (1), 133 (1), 143 (3), 159 (2), 171 (1), 189 (1, M^+-*t*Bu), 215 (0.12, M^+-CH_2OH), 228 (0.01, M^+-H_2O), 246 (0.003, M^+.

***anti*-5-(*tert*-Butyldimethylsilyloxy)-1-(*p*-toluenesulfonyloxy)-2,4-dimethylpentane (4)**

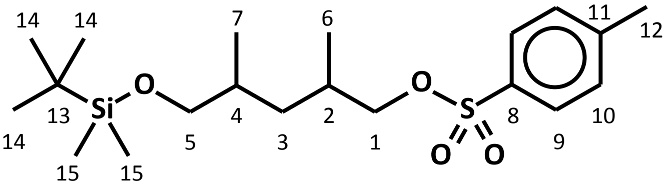

A solution of 1.521 g (6.17 mmol) **3**, 38 mg (0.31 mmol) DMAP and 1.3 mL (9.38 mmol) triethylamine in 50 mL dry dichloromethane was cooled to −20 °C. After the addition of 1.482 g (7.77 mmol) *p*-toluenesulfonyl chloride in small portions, the mixture was stirred at room temperature overnight. Subsequently, the solvent was largely removed in vacuo, and the residue was partitioned between 40 mL diethylether and 10 mL of a saturated aqueous solution of sodium hydrogen carbonate, work-up of the organic solution was carried out as described for the isolation of **3**. Column chromatography on silica (hexane:ethyl acetate = 15:1 to 6:1) yielded 2.398 g (5.98 mmol, 97%) of **4** as a colorless oil.

^{1}H-NMR, ^{1}H-^{1}H-COSY, HSQC, HMBC (400 MHz, $CDCl_3$): δ [ppm] = 0.01 (s, 6H, 2×CH_3 C15), 0.79 (d, 3H, $^3J_{H\text{-}H}$=6.6 Hz, CH_3 C7), 0.86 (d, 3H, $^3J_{H\text{-}H}$=6.6 Hz, CH_3 C6), 0.87 (s, 9H, 3×CH_3 C14), 1.05/1.17 (2ddd, 2H, $^2J_{H\text{-}H}$=13.2, $^3J_{H\text{-}H}$=9.4, 5.1 Hz, CH_2 C3), 1.55-1.65 (m, 1H, CH C4), 1.82-1.94 (m, 1H, CH C2), 2.45 (s, 3H, CH_3 C12), 3.34 (d, 2H, $^3J_{H\text{-}H}$=6.1 Hz, CH_2 C5), 3.82/3.87 (2dd, 2H, $^2J_{H\text{-}H}$=9.2, $^3J_{H\text{-}H}$=6.4/5.6 Hz, 1H, CH_2 C1), 7.34 (br.d, 2H, $^3J_{H\text{-}H}$=8.4 Hz, CH C10), 7.79 (br.d, 2H, $^3J_{H\text{-}H}$=8.4, Hz, CH C9).

^{13}C-NMR, HSQC, HMBC (101 MHz, $CDCl_3$): δ [ppm] = -5.26 (q, C15), 16.39/16.45 (2q, C6 C7), 18.45 (s, C13), 21.76 (q, C12), 26.07 (q, C14), 30.53 (d, C2), 33.04 (d, C4), 36.51 (t, C3), 68.71 (t, C5), 75.88 (t, C1), 128.05 (d, C9), 129.93 (d, C10), 133.52 (s, C11), 144.70 (s, C8).

MS (EI, 70 eV, GC/MS DB-5ms 30 m): m/z (%) = 39 (2), 41 (7), 42 (1), 43 (4), 45 (2), 47 (1), 53 (1), 55 (45), 56 (3), 57 (5), 58 (2), 59 (5), 60 (1), 61 (2), 63 (1), 65 (6), 67 (1), 69 (9), 70 (1), 73 (19), 74 (2), 75 (22), 76 (2), 77 (3), 79 (1), 81 (2), 83 (2), 85 (2), 87 (1), 88 (2), 89 (9), 90 (3), 91 (24), 92 (2), 95 (1), 97 (100), 98 (8), 99 (2), 101 (2), 105 (1), 107 (1), 113 (1), 115 (5), 116 (1), 122 (1), 129 (1), 139 (1), 143 (2), 149 (7), 150 (1), 151 (1), 155 (4), 165 (2), 171 (2), 172 (1), 229 (96), 230 (15), 231 (11), 232 (1), 247 (3), 271 (2), 285 (0.07), 343 (0.01, M^+-*t*Bu).

1-(*tert*-Butyldimethylsilyloxy)-2,4,8-trimethylnonane (5)

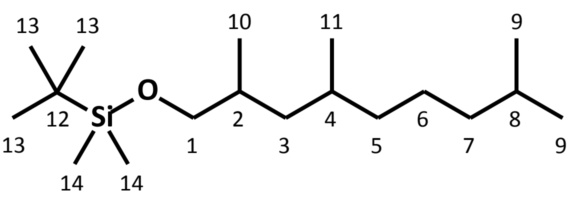

To a stirred solution of 1.150 g (2.87 mmol) of **4** and 25 mg (0.14 mmol) of P_2O_5-dried CuI in 20 mL dry THF, cooled to −90 °C, was dropwise added a solution of (3-methylbutyl)magnesium bromide, freshly prepared from 1.10 g (9.18 mmol) 1-bromo-3-methylbutane and 498 mg (20.49 mmol) magnesium in 20 mL dry THF. The stirred solution was warmed to room temperature and quenched at 0 °C by the addition of 10 mL saturated aqueous solution of ammonium chloride. After the addition of 30 mL diethyl ether, the organic layer was separated. Work-up as described for the isolation of **3**, followed by column chromatography on silica (hexane) yielded 7.67 mg (2.55 mmol, 89%) of crude **5**. After check by GC–MS the product was used in the next step without further purification.

MS (EI, 70 eV, GC/MS DB-5ms 30 m): m/z (%) = 39 (1), 41 (9), 42 (1), 43 (13), 45 (2), 47 (2), 55 (14), 56 (3), 57 (24), 58 (4), 59 (6), 61 (4), 67 (1), 69 (40), 70 (3), 71 (22), 72 (2), 73 (21), 74 (3), 75 (73), 76 (5), 77 (3), 81 (2), 83 (24), 84 (2), 85 (18), 86 (1), 87 (1), 88 (2), 89 (12), 90 (1), 95 (3), 97 (50), 98 (4), 99 (3), 101 (3), 103 (1), 109 (3), 111 (100), 112 (9), 113 (2), 115 (6), 116 (1), 125 (4), 129 (2), 167 (3), 185 (2), 243 (32), 244 (6), 245 (2), 285 (1), 286 (0.1), 300 (0.04, M^+).

***anti*-2,4,8-Trimethylnonan-1-ol (6)**

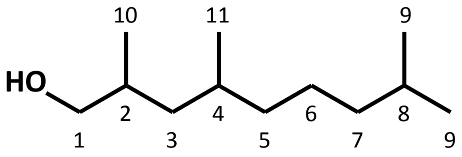

To a solution of 747 mg (2.48 mmol) of crude **5** in 20 mL THF was added 1.61 g (5.1 mmol) tetrabutylammonium fluoride. After stirring over night at room temperature, the solvent was removed in vacuo, and the residue was dissolved in 30 mL diethyl ether. After drying with magnesium sulfate and filtration over silica, column chromatography on silica (hexane:ethylacetate = 8:1) yielded 371 mg (1.99 mmol, 80%) of **6** as a colorless oil.

^{1}H-NMR, ^{1}H-^{1}H-COSY, HSQC, HMBC (400 MHz, $CDCl_3$): δ [ppm] = 0.84 (d, $^3J_{H\text{-}H}$=6.6 Hz, 3H, CH_3 C11), 0.87 (d, $^3J_{H\text{-}H}$=6.6 Hz, 6H, 2×CH_3 C9), 0.89 (d, $^3J_{H\text{-}H}$=6.6 Hz, 3H, CH_3 C10), 1.09-1.15/1.18-1.25 (2m, 2H, CH_2 C5), 1.07-1.17 (m, 2H, CH_2 C3), 1.11-1.17 (m, 2H, CH_2 C7), 1.25-1.32 (m, 2H, CH_2 C6), 1.44-1.55 (m, 1H, CH C4), 1.52 (tspt, $^3J_{H\text{-}H}$=6.6, 6.6 Hz, 1H, CH C8), 1.65-1.78 (m, 1H, CH C2), 3.40/3.48 (2dd, $^2J_{H\text{-}H}$=10.36, $^3J_{H\text{-}H}$=6.57, 4.80 Hz, 2H, CH_2 C1).

^{13}C-NMR, HSQC, HMBC (101 MHz, $CDCl_3$): δ [ppm] = 16.49 (q, C10), 19.54 (q, C11), 22.77/22.85 (2q, C9), 24.91 (t, C6), 28.13 (d, C8), 30.08 (d, C4), 33.41 (d, C2), 38.36 (t, C5), 39.47 (t, C7), 40.83 (t, C3), 69.27 (t, C1).
MS (EI, 70 eV, GC/MS): m/z (%) = 39 (7), 40 (2), 41 (42), 42 (10), 43 (52), 44 (2), 45 (6), 53 (4), 54 (2), 55 (65), 56 (58), 57 (100), 58 (6), 59 (8), 67 (5), 68 (4), 69 (36), 70 (48), 71 (38), 72 (2), 73 (5), 81 (2), 82 (4), 83 (65), 84 (18), 85 (15), 86 (1), 95 (2), 96 (1), 97 (14), 98 (20), 99 (7), 101 (7), 110 (2), 111 (14), 112 (7), 113 (2), 125 (1), 126 (20), 127 (2), 140 (2), 168 (2, M^+-H_2O).

2,4,8-Trimethylnonanal (7)

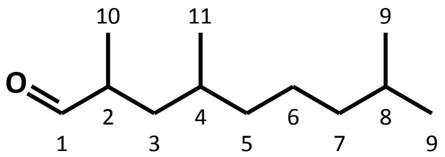

A suspension of 100 mg dry, finely ground molecular sieves 4 Å and 231 mg (1.24 mmol) **6** in 15 mL dry dichloromethane was cooled to −20 °C, and 745 mg (1.98 mmol) pyridinium dichromate were added in small portions. After stirring at room temperature for 90 min, the solvent was mostly removed, and the residue was taken up with 30 mL of a 1:1 mixture of pentane and diethyl ether. The dark brown solid material was filtered off over a silica pad, and the crude product was purified by column chromatography on silica (hexane:ethyl acetate = 20:1) yielding 169 mg (0.92 mmol, 74%) of **7** as a colorless oil.

Isomeric ratio by ^{1}H-NMR: *anti* : *syn* = 94 : 6.

^{1}H-NMR, ^{1}H-^{1}H-COSY, HSQC, HMBC (400 MHz, $CDCl_3$): δ [ppm] = 0.86 (d, $^3J_{H\text{-}H}$=6.6 Hz, 6H, 2×CH_3 C9), 0.87 (d, $^3J_{H\text{-}H}$=6.4 Hz, 3H, CH_3 C11), 1.07 (d, $^3J_{H\text{-}H}$=6.9 Hz, 3H, CH_3 C10), 1.10-1.19 (m, 2H, CH_2 C7), 1.13-1.20/1.48-1.58 (2m, 2H, CH_2 C5), 1.19-1.27 (m, 1H, CH C4), 1.21-1.33 (m, 2H, CH_2 C6), 1.23-1.30/1.47-1.56 (2m, 2H, CH_2 C3), 1.44-1.55 (m, 1H, CH C8), 2.41 (dqdd, $^3J_{H\text{-}H}$=8.7, 6.9, 5.1, 2.0 Hz, 1H, CH C2), 9.61 (d, $^3J_{H\text{-}H}$=2.0 Hz, 1H, CH C1).
^{13}C-NMR, HSQC, HMBC (101 MHz, $CDCl_3$): δ [ppm] = 14.78 (q, C10), 20.37 (q, C11), 22.84 (q, C9), 24.82 (t, C6), 28.11 (d, C8), 30.34 (d, C4), 37.74 (t, C3), 37.81 (t, C5), 39.34 (t, C7), 44.42 (d, C2), 205.71 (d, C1).
MS (EI, 70 eV, GC/MS DB-5ms 30 m): m/z (%) = 39 (8), 40 (2), 41 (44), 42 (9), 43 (81), 44 (3), 45 (1), 53 (4), 54 (1), 55 (30), 56 (27), 57 (87), 58 (61), 59 (3), 67 (6), 68 (4), 69 (31), 70

(21), 71 (100), 72 (11), 79 (2), 81 (10), 82 (3), 83 (7), 84 (3), 85 (30), 86 (2), 95 (15), 96 (6), 97 (4), 98 (6), 99 (6), 109 (8), 110 (3), 111 (5), 113 (1), 123 (3), 126 (43), 127 (11), 151 (1), 166 (0.1), 184 (0.2, M^+).

2,6,8,12-Tetramethyltrideca-2,4-diene (8)

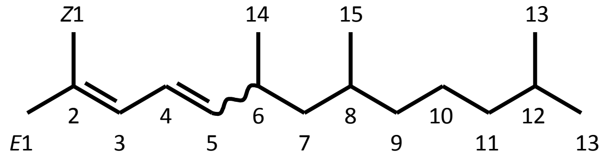

To a suspension of 675 mg (1.64 mmol) (3-methylbut-2-en-1-yl)triphenylphosphonium bromide [1] in 20 mL dry THF, cooled to −40 °C, was dropwise added 0.92 mL (1.47 mmol) of a 1.6 M *n*-BuLi solution in hexane. After slowly warming to room temperature, the bright orange solution was stirred for 2 h and then cooled again to −40 °C. After dropwise addition of 151 mg (0.82 mmol) of **7**, dissolved in 5 mL dry THF, the stirred solution was warmed to room temperature overnight. Subsequently, 1 mL of a saturated aqueous solution of ammonium chloride was added and the solvent removed in vacuo. After addition of magnesium sulfate, the slurry was extracted with three 20 mL portions of pentane. After filtration over celite, the extract was concentrated and submitted to column chromatography. Elution with pentane yielded 128 mg (0.54 mmol, 66%) of **8** as a colorless oil.

Isomeric ratios: (4*Z*) : (4*E*) = 1 : 2 (assignment by ^{1}H-NMR, order of elution by GC/MS); *anti* : *syn* = 97 : 3 (by GC/MS).

(4*E*)-*anti*-8

^{1}H-NMR, ^{1}H-^{1}H-COSY, HSQC, HMBC (500 MHz, $CDCl_3$): δ [ppm] = 0.84 (d, $^3J_{H-H}$=6.9 Hz, 3H, CH_3 C15), 0.86 (d, $^3J_{H-H}$=6.6 Hz, 6H, 2×CH_3 C13), 0.96 (d, $^3J_{H-H}$=6.9 Hz, 3H, CH_3 C14), 0.98-1.11/1.21-1.33 (2m, 2H, CH_2 C9), 1.07-1.15/1.16-1.25 (2m, 2H, CH_2 C7), 1.08-1.19 (m, 2H, CH_2 C11), 1.26-1.35 (m, 2H, CH_2 C10), 1.38-1.49 (m, 1H, CH C8), 1.48-1.56 (m, 1H, CH C12), 1.74 (s, 3H, CH_3 C*Z*1), 1.76 (s, 3H, CH_3 C*E*1), 2.26 (ddqd, $^3J_{H-H}$=7.9, 7.2, 6.9, 6.6 Hz, 1H, CH C6), 5.43 (dd, $^3J_{(E)H-H}$=15.1, $^3J_{H-H}$=7.9 Hz, 1H, CH C5), 5.78 (d, $^3J_{H-H}$=10.8 Hz, 1H, CH C3), 6.18 (dd, $^3J_{(E)H-H}$=15.1, $^3J_{H-H}$=10.8 Hz, 1H, CH C4).

^{13}C-NMR, HSQC, HMBC (101 MHz, $CDCl_3$): δ [ppm] = 18.37 (q, C*Z*1), 20.08 (q, C15), 20.78 (q, C14), 22.76 (c, C13), 24.71 (t, C10), 26.07 (q, C*E*1), 28.11 (d, C12), 30.31 (d, C8), 34.58 (d, C6), 37.27 (t, C9), 39.48 (t, C11), 45.05 (t, C7), 124.58 (d, C4), 125.36 (d, C3), 132.89 (s, C2), 138.82 (d, C5).

MS (EI, 70 eV, GC/MS DB-5ms 30 m): m/z (%) = 39 (2), 41 (15), 42 (1), 43 (15), 53 (3), 55 (12), 56 (2), 57 (7), 65 (2), 67 (26), 68 (3), 69 (12), 70 (1), 71 (3), 77 (4), 79 (6), 80 (2), 81 (17), 82 (35), 83 (6), 91 (6), 93 (7), 94 (2), 95 (29), 96 (8), 97 (4), 105 (1), 107 (7), 108 (2), 109 (100), 110 (18), 111 (3), 122 (2), 123 (14), 124 (3), 137 (1), 151 (9), 152 (2), 179 (1), 180 (5), 193 (1), 236 (15, M^+).

(4*Z*)-*anti*-8

^{1}H-NMR, ^{1}H-^{1}H-COSY, HSQC, HMBC (500 MHz, $CDCl_3$): δ [ppm] = 0.86 (d, $^3J_{H-H}$=6.9 Hz, 3H, CH_3 C15), 0.86 (d, $^3J_{H-H}$=6.3 Hz, 6H, 2×CH_3 C13), 0.93 (d, $^3J_{H-H}$=6.6 Hz, 3H, CH_3 C14), 0.98-1.11/1.21-1.33 (2m, 2H, CH_2 C9), 1.04-1.12/1.18-1.27 (2m, 2H, CH_2 C7), 1.08-1.19 (m, 2H, CH_2 C11), 1.19-1.26 (m, 2H, CH_2 C10), 1.38-1.49 (m., 1H, CH C8), 1.48-1.56 (m, 1H, CH C12), 1.74 (s, 3H, CH_3 C*Z*1), 1.80 (s, 3H, CH_3 C*E*1), 2.71 (dddq, $^3J_{H-H}$=9.5, 7.2, 7.2, 6.6 Hz, 1H, CH C6), 5.09 (dd, $^3J_{(Z)H-H}$=9.5, $^3J_{H-H}$=9,5 Hz, 1H, CH C5), 6.05 (d, *J*=11.7 Hz, 1H, CH C3), 6.08 (dd, $^3J_{H-H}$=11.7, $^3J_{(Z)H-H}$=9.5 Hz, 1H, CH C4).

^{13}C-NMR, HSQC, HMBC (101 MHz, $CDCl_3$): δ [ppm] = 18.18 (q, C*Z*1), 20.27 (q, C15), 21.29 (q, C14), 22.87 (q, C13), 24.83 (t, C10), 26.52 (q, C*E*1), 28.08 (d, C12), 29.57 (d, C6), 30.53 (d, C8), 37.21 (t, C9), 39.48 (t, C11), 45.49 (t, C7), 120.66 (d, C3), 122.95 (d, C4), 134.90 (s, C2), 136.71 (d, C5).

MS (EI, 70 eV, GC/MS DB-5ms 30 m): m/z (%) = 39 (3), 41 (18), 42 (2), 43 (18), 53 (4), 55 (15), 56 (2), 57 (8), 65 (2), 67 (29), 68 (3), 69 (13), 70 (1), 71 (3), 77 (5), 79 (7), 80 (2), 81 (21), 82 (42), 83 (7), 91 (7), 93 (8), 94 (2), 95 (34), 96 (9), 97 (5), 105 (1), 107 (7), 108 (2), 109 (100), 110 (19), 111 (3), 122 (2), 123 (15), 124 (4), 137 (2), 151 (11), 152 (2), 179 (1), 180 (6), 193 (1), 236 (12), 237 (2).

***anti*-5-Benzyloxy-2,4-dimethylpentan-1-ol (9)**

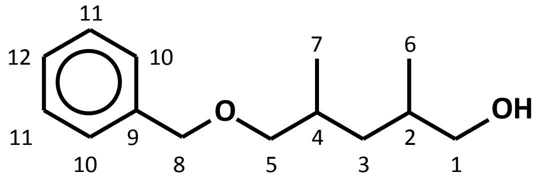

The synthesis of **9** was carried out similar to that of **3**. To a suspension of 188 mg (7.82 mmol) of sodium hydride in 30 mL dry THF, a solution of 1.034 g (7.82 mmol) *anti*-2,4-dimethylpentan-1,5-diol in 10 mL dry THF was added dropwise at −20 °C. After 1 h stirring at room temperature the mixture was cooled to 0 °C and 1.238 g (7.82 mmol) and 92 mg (0.25 mmol) tetra-*n*-butylammonium iodide. After work-up as described for the

synthesis of **3** and Kugelrohr distillation at 5 mbar, 1.234 g (5.55 mmol, 71%) of **9** was obtained as colorless oil.

^{1}H-NMR, ^{1}H-^{1}H-COSY, HSQC, HMBC (400 MHz, $CDCl_3$): δ [ppm] = 0.90 (d, $^3J_{H\text{-}H}$=6.8 Hz, 3H, CH_3 C6), 0.92 (m, $^3J_{H\text{-}H}$=6.8 Hz, 3H, CH_3 C7), 1.23 (ddd, $^2J_{H\text{-}H}$=13.7, $^3J_{H\text{-}H}$=8.9, 4.8 Hz, 2H, CH_2 C3), 1.71-1.82 (m, 1H, CH C2), 1.84-1.97 (m, 1H, CH C4), 3.28/3.30 (2dd, $^2J_{H\text{-}H}$=12.3, $^3J_{H\text{-}H}$=6.5/6.5 Hz, 2H, CH_2 C5), 3.43/3.48 (2dd, $^3J_{H\text{-}H}$=10.5, $^3J_{H\text{-}H}$=6.3/6.3 Hz, 1H, CH_2 C1), 4.51 (s, 2H, CH_2 C8), 7.26-7.31 (m, 1H, CH C12), 7.31-7.36 (m, 4H, 2× CH C10 C11).

^{13}C-NMR, HSQC, HMBC (101 MHz, $CDCl_3$): δ [ppm] = 16.47 (q, C6), 17.15 (q, C7), 30.76 (d, C4), 33.21 (d, C2), 37.41 (t, C3), 69.04 (t, C1), 73.20 (t, C8), 76.75 (t, C5), 127.64 (d, C12), 127.71 (d, C10), 128.49 (d, C11), 138.78 (s, C9).

MS (EI, 70 eV, GC/MS DB-5ms 30 m): m/z (%) = 39 (7), 41 (14), 42 (4), 43 (18), 45 (6), 51 (3), 53 (1), 55 (28), 56 (5), 57 (8), 58 (2), 59 (6), 63 (2), 65 (14), 67 (1), 69 (5), 70 (2), 71 (2), 77 (4), 78 (1), 79 (4), 81 (1), 83 (11), 85 (1), 89 (3), 90 (1), 91 (100), 92 (27), 93 (2), 95 (7), 96 (1), 97 (3), 99 (1), 105 (2), 106 (1), 107 (37), 108 (13), 109 (1), 113 (7), 131 (3), 147 (3), 204 (0.3), 207 (0.2), 222 (1, M^+).

***anti*-5-Benzyloxy-2,4-dimethylpentanal (10)**

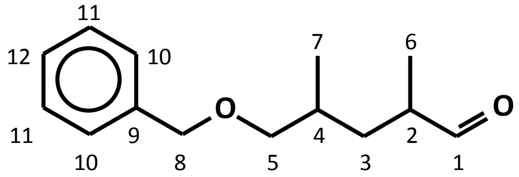

A suspension of 500 mg finely ground molecular sieves 4 Å and 1.201 g (5.40 mmol) **9** in 20 mL dry dichloromethane was cooled to −20 °C, and 1.382 g (6.41 mmol) pyridinium chlorochromate were added in small portions. After stirring at room temperature for 90 min the reaction mixture was worked-up as described for the synthesis of **7**. Column chromatography on silica (hexane:ethyl acetate = 25:1) yielded 868 mg (3.94 mmol, 73%) of **10** as a colorless oil.

Isomeric ratio by ^{1}H-NMR: *anti* : *syn* = 9 : 1.

^{1}H-NMR, ^{1}H-^{1}H-COSY, HSQC, HMBC (500 MHz, $CDCl_3$): δ [ppm] = 0.94 (d, $^3J_{H\text{-}H}$=6.6 Hz, 3H, CH_3 C7), 1.08 (d, $^3J_{H\text{-}H}$=6.9 Hz, 3H, CH_3 C6), 1.46/1.57 (2ddd, $^2J_{H\text{-}H}$=14.0, $^3J_{H\text{-}H}$=8.2, 5.4/8.4, 6.0 Hz, 2H, CH_2 C3), 1.83-1.94 (m, 1H, CH C4), 2.40-2.48 (m, 1H, CH C2),

3.31/3.32 (2dd, ${}^{3}J_{H\text{-}H}$=10.9, ${}^{3}J_{H\text{-}H}$=6.1/6.1 Hz, 2H, CH_2 C5), 4.49 (s, 2H, CH_2 C8), 7.26-7.30 (m, 1H, CH C12), 7.31-7.37 (m, 4H, 2× CH C10 C11), 9.61 (d, ${}^{3}J_{H\text{-}H}$=1.9 Hz, 1H, CHO C1).
^{13}C-NMR, HSQC, HMBC (126 MHz, $CDCl_3$): δ [ppm] = 13.61 (q, C6), 17.00 (q, C7), 31.17 (d, C4), 34.51 (t, C3), 44.18 (d, C2), 73.20 (t, C8), 75.81 (t, C5), 127.67 (d, C12), 127.68 (d, C10), 128.50 (d, C11), 138.69 (s, C9), 205.38 (d, C1).
MS (EI, 70 eV, GC/MS DB-5ms 30 m): m/z (%) = 39 (6), 41 (11), 42 (2), 43 (28), 45 (3), 51 (3), 53 (1), 55 (12), 56 (2), 57 (5), 58 (2), 59 (3), 63 (2), 65 (14), 69 (3), 70 (1), 71 (13), 77 (4), 78 (1), 79 (4), 81 (2), 83 (11), 89 (2), 90 (1), 91 (100), 92 (25), 93 (2), 95 (2), 96 (2), 99 (1), 101 (1), 105 (1), 107 (12), 108 (4), 111 (2), 112 (5), 113 (4), 114 (2), 119 (1), 129 (3), 148 (0.3), 161 (0.3), 187 (1), 202 (0.2), 220 (1, M^+).

(5*E*/*Z*,7*E*)-*anti*-1-Benzyloxy-2,4,6-trimethylnona-5,7-diene (11)

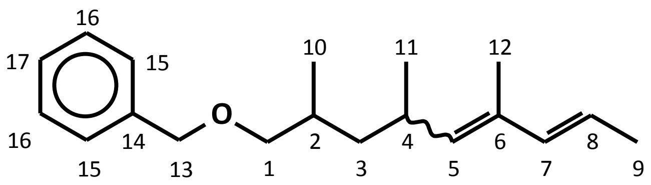

To a suspension of 3.167 g (7.70 mmol) [(2*E*)-1-methylbut-2-enyl]triphenylphosphonium bromide [2] in 20 mL dry THF, cooled to −40 °C was dropwise added 4.35 mL (6.96 mmol) of a 1.6 M *n*-BuLi solution in hexane. After slowly warming to room temperature, the bright orange solution was stirred for 2 h and then cooled again to −40 °C. After dropwise addition of 849 mg (3.85 mmol) **10** dissolved in 10 mL dry THF, the stirred solution was warmed to room temperature overnight. Work-up of the mixture was carried out as described for the synthesis of **8**. Column chromatography on silica (hexane:ethyl acetate = 40:1) yielded 604 mg (2.22 mmol, 58%) of **11** as a colorless oil.

Isomeric ratios by GC/MS: (5*Z*) : (5*E*) = 1 : 2 (order of elution); *anti* : *syn* = 4 : 1.

(5*E*)-*anti*-11
^{1}H-NMR, ^{1}H-^{1}H-COSY, HSQC, HMBC (400 MHz, $CDCl_3$): δ [ppm] = 0.91 (d, ${}^{3}J_{H\text{-}H}$=6.6 Hz, 3H, CH_3 C11), 0.94 (d, ${}^{3}J_{H\text{-}H}$=6.9 Hz, 3H, CH_3 C10), 1.03-1.18/1.30-1.45 (2m, 2H, CH_2 C3), 1.68 (d, ${}^{4}J_{H\text{-}H}$=1.3 Hz, 3H, CH_3 C12), 1.75 (dd, ${}^{3}J_{H\text{-}H}$=6.6, ${}^{4}J_{H\text{-}H}$=1.2 Hz, 3H, CH_3 C9), 2.45-2.60 (m, 1H, CH C4), 3.31/3.33 (2dd, ${}^{2}J_{H\text{-}H}$=9.1/9.1, ${}^{3}J_{H\text{-}H}$=5.1/5.1 Hz, 2H, CH_2 C1), 4.48 (s, 2H, CH_2 C13), 5.13 (br.d, ${}^{3}J_{H\text{-}H}$=9.7 Hz, 1H, HC C5), 5.55 (dq, ${}^{2}J_{H\text{-}H}$=15.5, ${}^{3}J_{H\text{-}H}$=6.6 Hz, 1H, CH C8), 6.04 (dq, ${}^{3}J_{H\text{-}H}$=15.5, 1.2 Hz, 1H, HC C7), 7.26-7.30 (m, 1H, CH C17), 7.31-7.35 (m, 4H, 2× CH C15 C16).

^{13}C-NMR, HSQC, HMBC (101 MHz, $CDCl_3$): δ [ppm] = 12.71 (q, C12), 17.95 (q, C10), 18.36 (q, C9), 21.07 (q, C11), 30.06 (d, C4), 31.50 (d, C2), 41.70 (t, C3), 73.08 (t, C13), 75.81 (t, C1), 122.22 (d, C8), 127.53 (d, C17), 127.65 (d, C15), 128.44 (d, C16), 131.86 (s, C6), 136.36 (d, C5), 137.21 (d, C5), 138.99 (s, C14).

MS (EI, 70 eV, GC/MS DB-5ms 30 m): m/z (%) = 39 (8), 41 (30), 43 (36), 51 (3), 53 (6), 55 (24), 57 (8), 65 (17), 67 (31), 68 (2), 69 (84), 70 (5), 71 (3), 77 (11), 78 (2), 79 (15), 81 (16), 82 (4), 83 (11), 85 (4), 91 (100), 92 (9), 93 (11), 94 (3), 95 (15), 96 (4), 97 (7), 99 (9), 105 (6), 107 (27), 108 (4), 109 (28), 110 (4), 111 (9), 119 (5), 121 (25), 122 (4), 123 (22), 124 (5), 125 (3), 135 (4), 137 (2), 140 (7), 148 (7), 149 (2), 163 (10), 181 (3), 188 (0.9), 272 (0.2, M^+).

(5*Z*)-*anti*-11

^{1}H-NMR, ^{1}H-^{1}H-COSY, HSQC, HMBC (400 MHz, $CDCl_3$): δ [ppm] = 0.91 (d, $^3J_{H-H}$=6.6 Hz, 3H, CH_3 C11), 0.95 (d, $^3J_{H-H}$=6.6 Hz, 3H, CH_3 C10), 1.03-1.18/1.30-1.45 (2m, 2H, CH_2 C3), 1.77 (d, $^4J_{H-H}$=1.3 Hz, 3H, CH_3 C12), 1.78 (dd, $^3J_{H-H}$=6.6, $^4J_{H-H}$=1.3 Hz, 3H, CH_3 C9), 2.61-2.75 (m, 1H, CH C4), 3.20/3.22 (2dd, $^2J_{H-H}$=7.1/7.1, $^3J_{H-H}$=7.1/7.1 Hz, 2H, CH_2 C1), 4.49 (s, 2H, CH_2 C13), 5.00 (br.d, $^3J_{H-H}$=9.7 Hz, 1H, HC C5), 5.67 (dq, $^2J_{H-H}$=15.5, $^2J_{H-H}$=6.6 Hz, 1H, CH C8), 6.39 (br.d, $^3J_{H-H}$=15.5 Hz, 1H, HC C7), 7.26-7.30 (m, 1H, CH C17), 7.31-7.35 (m, 4H, 2× CH C15 C16).

^{13}C-NMR, HSQC, HMBC (101 MHz, $CDCl_3$): δ [ppm] = 17.98 (q, C10), 18.83 (q, C9), 20.89 (q, C12), 21.34 (q, C11), 29.10 (q, C4), 31.38 (d, C2), 41.84 (t, C3), 73.08 (t, C13), 75.93 (t, C1), 125.23 (d, C8), 127.53 (d, C17), 127.65 (d, C15), 128.44 (d, C16), 128.78 (d, C7), 130.17 (s, C6), 135.52 (d, C7), 138.98 (s, C14).

2,4,6-Trimethylnonan-1-ol (12)

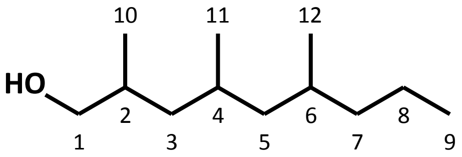

To a solution of 555 mg (2.15 mmol) **11** in 50 mL pentane was added 117 mg Pd/C 10%. The suspension was stirred under 20 bar hydrogen at room temperature for 48 h. Subsequently, the catalyst was removed by filtration over silica, and the crude product was purified by column chromatography on silica (hexane:ethyl acetate = 45:1) yielding 377 mg (2.02 mmol, 94%) of **12** as a colorless oil.

Isomeric ratios by GC/MS: *syn,syn* : *syn,anti* = *anti,syn* : *anti,anti* = 58 : 42; *syn,syn* : *anti,syn* = *syn,anti* : *anti,anti* = 17:83 (order of elution).

NMR-data of *syn,syn*-2,4,6-trimethylnonan-1-ol are provided with **20.**

MS (EI, 70 eV, GC/MS DB-5ms 30 m): m/z (%) = 39 (9), 40 (2), 41 (52), 42 (15), 43 (91), 44 (3), 45 (8), 53 (4), 54 (2), 55 (81), 56 (27), 57 (100), 58 (7), 59 (9), 67 (5), 68 (22), 69 (64), 70 (60), 71 (53), 72 (3), 81 (4), 82 (4), 83 (79), 84 (27), 85 (24), 86 (2), 95 (1), 96 (2), 97 (14), 98 (5), 99 (8), 101 (6), 110 (1), 111 (24), 112 (5), 113 (1), 124 (1), 125 (24), 126 (10), 127 (1), 139 (1), 153 (1), 168 (0.3), 185 (0.1), 186 (0.04, M^+).

2,4,6-Trimethylnonanal (13)

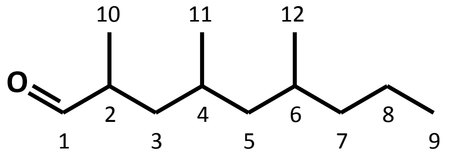

The oxidation of **12** to **13** was carried out the same way as that of **6** to **7**, using 352 mg (1.89 mmol) of **12** and 1.138 g (3.02 mmol) pyridinium dichromate and 200 mg finely ground molecular sieves 4 Å in 30 mL dichloromethane. After work-up and column chromatography, 324 mg (1.76 mmol, 93%) of **13** were obtained as a colorless oil.

NMR data of *syn,syn*-2,4,6-trimethylnonan-1-ol are provided with **21.**

***anti,anti*-13**

MS (EI, 70 eV, GC/MS DB-5ms 30 m): m/z (%) = 39 (12), 40 (1), 41 (48), 42 (12), 43 (100), 44 (3), 53 (5), 55 (37), 56 (9), 57 (68), 58 (45), 59 (2), 65 (2), 67 (4), 68 (2), 69 (21), 70 (16), 71 (80), 72 (8), 77 (1), 79 (2), 81 (9), 82 (4), 83 (17), 84 (4), 85 (19), 86 (1), 93 (1), 95 (8), 96 (5), 97 (3), 99 (4), 109 (2), 111 (5), 113 (2), 123 (7), 124 (1), 126 (18), 127 (8), 141 (1), 184 (0.3, M^+).

2,6,8,10-Tetramethyltrideca-2,4-diene (14)

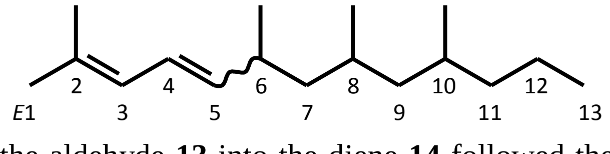

The transformation of the aldehyde **13** into the diene **14** followed the same way as that of **7** into **8**. The Wittig reaction was carried out in 20 mL dry THF, using 781 mg (1.90 mmol) (3-methylbut-2-en-1-yl)triphenylphosphonium bromide [1] and 175 mg (0.95 mmol) of **13**, yielding 155 mg (0.66 mmol, 69%) of **14** as a colorless oil.

Isomeric ratios by GC/MS: (4*Z*) : (4*E*) = 25 : 75; *syn*,*syn* : *syn*,anti : *anti*,*syn* : *anti*,*anti* = 14 : 13 : 43 : 30 (order of elution).

(4*E*)-*anti*,*syn*-14 + (4*E*)-*anti*,*anti*-14

^{1}H-NMR, ^{1}H-^{1}H-COSY, HSQC, HMBC (400 MHz, $CDCl_3$): δ [ppm] = 0.80/0.81/0.83/0.84 (4d, $^3J_{H-H}$=6.6/6.6/6.6/6.6 Hz, 6H, 2×CH_3 C15 C16), 0.87/0.88 (2t, $^3J_{H-H}$=7.2/7.2 Hz, 3H, CH_3 C13), 0.87-0.95/1.01-1.11 (2m, 2H, CH_2 C9), 0.96/0.96 (2d, $^3J_{H-H}$=6.6 Hz, 3H, CH_3 C14), 1.04-1.14 (m 2H, CH_2 C11), 1.07-1.17/1.18-1.26 (2m, 2H, CH_2 C7), 1.12-1.36 (m, 2H, CH_2 C12), 1.41-1.51 (m, 1H, C10), 1.46-1.59 (m, 1H, C8), 1.74 (br.s, 3H, CH_3 C*Z*1), 1.75 (br.s, 3H, CH_3 C*E*1), 2.27 (dddq, $^3J_{H-H}$=7.8, 7.2, 7.2, 6.6 Hz, 1H, CH C6), 5.37/5.43 (2dd, $^3J_{(E)H-H}$=15.2/14.9, $^3J_{H-H}$=7.8/7.8 Hz, 1H, CH C5), 5.78 (br.d, $^3J_{H-H}$=10.6 Hz, 1H, CH C3), 6.18 (dd, $^3J_{(E)H-H}$=15.1, $^3J_{H-H}$=10.6 Hz, 1H, CH C4).

^{13}C-NMR, HSQC, HMBC (126 MHz, $CDCl_3$): δ [ppm] = 14.53/14.57 (2q, C13), 18.35 (q, C*Z*1), 19.67/19.74 (2q, C16), 20.12/20.25 (2q, C12), 20.56/20.62 (2q, C15), 20.70/20.94 (q, C14), 26.05 (q, C*E*1), 27.78/27.81 (2d, C8), 29.90/29.98 (2d, C10), 34.56 (d, C6), 39.18/39.51 (2t, C11), 44.84/45.42 (2t, C9), 45.12/45.90 (2t, C7), 124.62/124.77 (2d, C4), 125.34/125.41 (2d, C3), 132.88/133.14 (2s, C2), 138.61/138.84 (2d, C5).

(4*E*)-*syn*,*syn*-14

MS (EI, 70 eV, GC/MS DB-5ms 30 m): m/z (%) = 39 (5), 41 (25), 42 (2), 43 (31), 44 (1), 53 (4), 55 (19), 56 (2), 57 (8), 65 (3), 67 (34), 68 (3), 69 (23), 70 (2), 71 (6), 77 (6), 78 (1), 79 (9), 80 (3), 81 (23), 82 (45), 83 (8), 91 (6), 93 (7), 94 (2), 95 (19), 96 (6), 97 (4), 105 (1), 107 (6), 108 (2), 109 (100), 110 (16), 111 (6), 122 (1), 123 (13), 124 (4), 125 (1), 137 (3), 151 (10), 152 (3), 180 (1), 193 (3), 236 (9, M^+)

(4*Z*)-*anti*,*syn*-14 + (4*Z*)-*anti*,*anti*-14

^{1}H-NMR, ^{1}H-^{1}H-COSY, HSQC, HMBC (400 MHz, $CDCl_3$): δ [ppm] = 0.81/0.81/0.83/0.86 (4d, $^3J_{H-H}$=6.6/6.6/6.6/6.6 Hz, 6H, 2×CH_3 C15 C16), 0.86/0.87 (2t, $^3J_{H-H}$=7.2/7.2 Hz, 3H, CH_3 C13), 0.87-0.95/1.01-1.11 (2m, 2H, CH_2 C9), 0.93/0.93 (2d, $^3J_{H-H}$=6.6 Hz, 3H, CH_3 C14), 1.07-1.17/1.18-1.26 (2m, 2H, CH_2 C7), 1.09-1.21 (m 2H, CH_2 C11), 1.12-1.36 (m, 2H, CH_2 C12), 1.42-1.56 (m, 1H, C10), 1.48-1.62 (m, 1H, C8), 1.74 (br.s, 3H, CH_3 C*Z*1), 1.80 (br.s, 3H, CH_3 C*E*1), 2.65-2.77 (m, 1H, CH C6), 5.00- 5.14 (m, 1H, CH C5), 6.03-6.12 (m, 2H, 2×CH C3 CH C4).

^{13}C-NMR, HSQC, HMBC (126 MHz, $CDCl_3$): δ [ppm] = 14.51/14.55 (2q, C13), 18.04 (q, C*Z*1), 20.02/20.07 (2q, C16), 20.17/20.27 (2q, C12), 20.62/20.79 (2q, C15), 21.40/21.77 (2q, C14), 26.50 (q, C*E*1), 27.85/27.90 (2d, C8), 29.52 (d, C6), 29.82/29.93 (2d, C10), 40.35/40.61

(2t, C11), 44.80/45.55 (2t, C9), 45.05/45.82 (2t, C7), 120.16/120.24 (2d, C3), 120.67/120.74 (2d, C4), 134.59/134.72 (2s, C2), 136.66/136.80 (2d, C5).

(4*Z*)-*syn,syn*-14

MS (EI, 70 eV, GC/MS DB-5ms 30 m): m/z (%) = 39 (5), 40 (1), 41 (28), 42 (3), 43 (37), 44 (1), 53 (5), 55 (18), 56 (2), 57 (9), 65 (3), 66 (1), 67 (38), 68 (4), 69 (28), 70 (3), 71 (8), 77 (7), 78 (1), 79 (10), 80 (2), 81 (24), 82 (51), 83 (10), 84 (1), 85 (1), 91 (8), 93 (8), 94 (2), 95 (25), 96 (8), 97 (5), 105 (1), 107 (6), 108 (2), 109 (100), 110 (18), 111 (8), 112 (1), 122 (2), 123 (17), 124 (5), 125 (1), 137 (4), 151 (15), 152 (4), 165 (1), 180 (2), 193 (6), 194 (1), 221 (1), 236 (13, M^+).

(2*E*,4*E*/*Z*)-4,6,8,10-Tetramethyltrideca-2,4-diene (15)

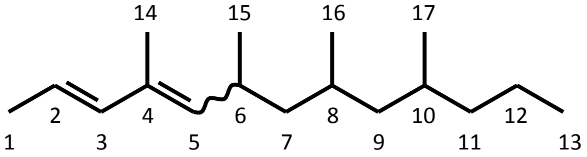

The transformation of the aldehyde **13** into the diene **15** was equivalent to that yielding **8** from **7**. The Wittig reaction was carried out in 20 mL dry THF using 719 mg (1.75 mmol) [(*E*)-1-methylbut-2-en-1-yl]triphenylphosphonium bromide [2] and 161 mg (0.87 mmol) of **13** and yielding 111 mg (0.47 mmol, 54%) of **15** as a colorless oil.

Isomeric ratios by GC/MS: (4*Z*) : (4*E*) = 38 : 62; (2*E*,4*Z*)-*syn,syn* : (2*E*,4*Z*)-*syn,anti* + (2*E*,4*Z*)-*anti,syn* : (2*E*,4*Z*)-*anti,anti* : (2*E*,4*E*)-*syn,syn* : (2*E*,4*E*)-*syn,anti* : (2*E*,4*E*)-*anti,syn* : (2*E*,4*E*)-*anti,anti* = 6 : 22 : 11 : 8 : 8 : 28 : 17 (order of elution).

NMR-spectroskopic data of (2*E*,4*E*)-*syn,syn*- and (2*E*,4*Z*)-*syn,syn*-**15** are provided with **22**.

(4*E*)-*anti,syn*-15

MS (EI, 70 eV, GC/MS DB-5*ms* 30 m): m/z (%) = 39 (4), 41 (26), 42 (2), 43 (31), 53 (4), 55 (16), 56 (2), 57 (8), 65 (3), 67 (42), 68 (3), 69 (37), 70 (4), 71 (7), 77 (6), 78 (1), 79 (10), 80 (1), 81 (20), 82 (49), 83 (12), 84 (2), 85 (2), 91 (8), 93 (8), 94 (3), 95 (32), 96 (6), 97 (8), 105 (1), 107 (5), 108 (2), 109 (100), 110 (15), 111 (8), 112 (1), 123 (12), 124 (3), 125 (1), 137 (2), 151 (6), 152 (3), 153 (4), 193 (1), 236 (7, M^+).

***syn,syn*-4,6-Dimethyl-7-(triphenylmethyloxy)heptan-2-ol (17)**

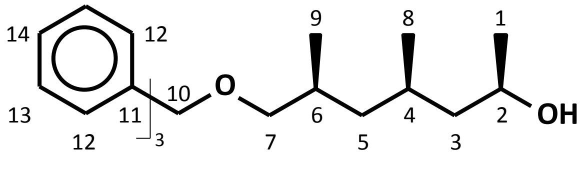

+ ent

To a solution of 2.715 g (16.94 mmol) of *syn,syn*-2,4-dimethylheptan-1,6-diol (**16**) [3,4], 108 mg (0.88 mol) DMAP, and 3.5 mL (25.25 mmol) triethylamine in 50 mL dry dichloromethane was portion-wise added 5.19 g (18.6 mmol) triphenylmethyl chloride at room temperature. After stirring overnight, the solvent was mostly removed in vacuo, and the residue was taken up in diethyl ether. Work-up as described for the isolation of **3** and column chromatography on silica (hexane : ethyl acetate = 10 : 1 to 4 : 1) yielded 5.338 g (13.26 mmol, 78%) of **17** as a colorless oil.

^{1}H-NMR, ^{1}H-^{1}H-COSY, HSQC, HMBC (400 MHz, $CDCl_3$): δ [ppm] = 0.85 (d, $^3J_{H\text{-}H}$=6.5 Hz, 3H, CH_3 C8), 0.96/1.33 (2ddd, $^2J_{H\text{-}H}$=13.6, $^3J_{H\text{-}H}$=7.0, 7.0/6.9, 6.9 Hz, 2H, CH_2 C5), 0.98 (d, $^3J_{H\text{-}H}$=6.8 Hz, 3H, CH_3 C9), 1.07/1.43 (2ddd, $^2J_{H\text{-}H}$=13.8, $^3J_{H\text{-}H}$=9.8, 3.5/9.3, 4.0 Hz, 2H, CH_2 C3), 1.16 (d, $^3J_{H\text{-}H}$=6.27 Hz, 3H, CH_3 C1), 1.53-1.64 (m, 1H, CH C4), 1.83 (tqdd, $^3J_{H\text{-}H}$=7.0, 6.8, 6.5, 5.3 Hz, 1H, CH C6), 2.85/2.98 (2dd, $^2J_{H\text{-}H}$=8.8, $^3J_{H\text{-}H}$=6.5/5.3 Hz, 2H, CH_2 C7), 3.86 (dqd, $^3J_{H\text{-}H}$=10.0, 5.8, 3.6 Hz, 1H, CH C2), 7.20-7.25 (m, 3H, CH C14), 7.27-7.32 (m, 6H, CH C13), 7.43-7.47 (m, 6H, CH C12).

^{13}C-NMR, HSQC, HMBC (126 MHz, $CDCl_3$): δ [ppm] = 18.45 (q, C9), 20.18 (q, C8), 24.51 (q, C1), 26.90 (d, C4), 31.41 (d, C6), 42.37 (t, C5), 46.80 (t, C3), 65.83 (d, C2), 68.46 (t, C7), 86.25 (s, C10), 126.93 (d, C14), 127.80 (d, C13), 128.91 (d, C12), 144.66 (s, C11).

MS (EI, 70 eV, GC/MS DB-5ms 30 m): m/z (%) = 39 (1), 41 (7), 42 (1), 43 (6), 45 (16), 51 (1), 55 (7), 56 (2), 57 (4), 69 (9), 71 (1), 77 (7), 78 (1), 83 (6), 91 (2), 99 (1), 105 (22), 106 (2), 115 (3), 123 (2), 125 (1), 141 (3), 143 (2), 152 (1), 154 (2), 155 (1), 159 (4), 164 (2), 165 (38), 166 (7), 167 (3), 181 (1), 183 (26), 184 (4), 202 (2), 215 (3), 226 (2), 227 (1), 228 (5), 229 (1), 239 (4), 240 (2), 241 (5), 242 (3), 243 (100, Ph_3C^+), 244 (34), 245 (5), 258 (3), 259 (12), 260 (3), 279 (0.1), 280 (0.1), 281 (0.2), 307 (0.1), 325 (2, M^+-Ph), 326 (0.5), 327 (0.1), 354 (0.1), 383 (0.1), 384 (0.2, M^+-H_2O), 385 (0.1), 402 (0.05, M^+).

***anti,syn*-4,6-Dimethyl-2-(3’,5’-dinitrobenzoyloxy)-7-(triphenylmethyloxy)heptane**

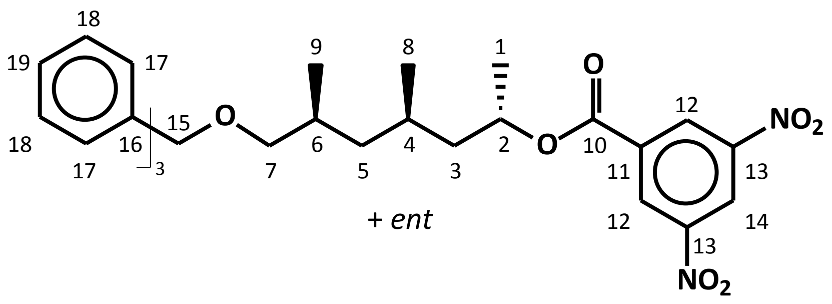

To a solution of 3.490 g (13.30 mmol) triphenylphosphane in 175 mL dry THF, cooled to −20 °C, was dropwise added 2.48 mL (12.6 mmol) diisopropyl azodicarboxylate. The yellow solution decolorized and became turbid after 30 min. Subsequently, 2.614 g (12.33 mmol) of 3,5-dinitrobenzoic acid were added in small portions. After stirring for 40 min at room temperature, a solution of 2.612 g (6.49 mmol) **17** in 5 mL THF was slowly added, and stirring was continued for 17 h. Thereafter, the solvent was removed in vacuo, and the residue was purified by column chromatography on silica (hexane:ethyl acetate = 8:1) yielding 3.846 g of crude *anti,syn*-4,6-dimethyl-2-(3',5'-dinitrobenzoyloxy)-7-(triphenylmethyl-oxy)heptane. After characterization by its mass spectrum, the product was used in the next step without further purification.

MS (EI, 70 eV, direct): m/z (%) = 40 (6), 41 (6), 43 (6), 55 (10), 57 (5), 69 (19), 70 (7), 71 (3), 77 (2), 82 (3), 83 (9), 84 (3), 85 (3), 95 (2), 97 (2), 105 (21), 111 (3), 112 (3), 149 (2), 165 (24), 166 (4), 183 (23), 184 (2), 195 (5), 228 (2), 239 (2), 241 (5), 242 (5), 243 (100, Ph_3C^+), 244 (34), 245 (4), 259 (8), 520 (4, M^+-Ph), 597 (3, M^+).

anti,syn-4,6-Dimethyl-7-(triphenylmethyloxy)heptan-2-ol (18)

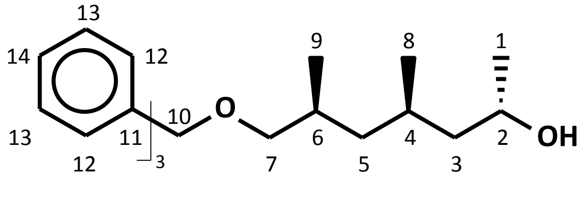

To 3.846 g (6.43 mmol) of *anti,syn*-4,6-dimethyl-2-(3',5'-dinitrobenzoyloxy)-7-(triphenylmethyloxy)heptane, dissolved in a mixture of 30 mL methanol and 30 mL THF, was added 6.5 mL of a 2 M aqueous solution of sodium hydroxide. After stirring for 45 min at room temperature, the volume of the solution was reduced by half (in vacuo), and 60 mL diethyl ether were added. The organic solution was washed with five 20 mL portions of saturated aqueous sodium hydrogencarbonate solution and brine. After drying with magnesium sulfate and concentration in vacuo, the crude product was purified by column chromatography on silica (hexane:ethyl acetate = 10:1 to 4:1) yielding 2.572 g (6.39 mmol, 98% over 2 steps) **18** as a colorless oil.

^{1}H-NMR, ^{1}H-^{1}H-COSY, HSQC, HMBC (400 MHz, $CDCl_3$): δ [ppm] = 0.85 (d, $^3J_{H\text{-}H}$=6.6 Hz, 3H, CH_3 C8), 0.94 (d, $^3J_{H\text{-}H}$=6.6 Hz, 3H, CH_3 C9), 1.01/1.35 (2ddd, $^2J_{H\text{-}H}$=13.6, $^3J_{H\text{-}H}$=7.0, 7.0/6.9, 6.9 Hz, 2H, CH_2 C5), 1.12/1.48 (2ddd, $^3J_{H\text{-}H}$=13.9, $^3J_{H\text{-}H}$=9.7, 3.5/9.5, 4.1 Hz, 2H, CH_2 C3), 1.20 (d, $^3J_{H\text{-}H}$=6.3 Hz, 3H, CH_3 C1), 1.53-1.63 (m, 1H, CH C4), 1.74 (tqdd, $^3J_{H\text{-}}$

$_{H}$=7.0, 6.6, 6.3, 5.0 Hz, 1H, CH C6), 3.43/3.51 (2dd, $^{2}J_{H\text{-}H}$=10.7, $^{3}J_{H\text{-}H}$=6.3/5.0 Hz, 2H, CH_2 C7), 3.92 (dqd, $^{3}J_{H\text{-}H}$=10.0, 6.0, 3.5 Hz, 1H, CH C2), 7.20-7.25 (m, 3H, CH C14), 7.27-7.31 (m, 12H, 2×CH C 12 C13).

^{13}C-NMR, HSQC, HMBC (126 MHz, $CDCl_3$): δ [ppm] = 17.50 (q, C9) 20.44 (q, C8) 24.70 (q, C1) 27.05 (d, C4) 33.12 (d, C6) 41.77 (t, C5) 46.65 (t, C3) 66.05 (d, C2) 68.29 (t, C7) 82.15 (s, C10) 127.40 (d, C14) 128.07/147.01 (2d, C12 C13), 147.01 (s, C11).

MS (EI, 70 eV, GC/MS DB-5ms 30 m): m/z (%) = 39 (1), 41 (7), 42 (1), 43 (7), 45 (16), 51 (1), 55 (8), 56 (2), 57 (4), 69 (10), 70 (1), 71 (1), 77 (9), 78 (1), 83 (8), 91 (2), 99 (2), 105 (27), 106 (2), 115 (3), 123 (2), 125 (2), 139 (1), 141 (3), 143 (2), 152 (2), 154 (3), 155 (1), 159 (5), 163 (1), 164 (2), 165 (44), 166 (8), 167 (3), 181 (1), 183 (27), 184 (4), 202 (2), 215 (3), 226 (2), 227 (1), 228 (6), 229 (1), 239 (4), 240 (2), 241 (5), 242 (3), 243 (100, Ph_3C^+), 244 (33), 245 (5), 258 (3), 259 (12), 260 (3), 279 (0.1), 280 (0.1), 325 (2), 326 (0.4), 354 (0.1), 384 (0.2, M^+-H_2O), 402 (0.04, M^+).

***anti,syn*-2-(Methanesulfonyloxy)-4,6-dimethyl-7-(triphenylmethyloxy)heptane**

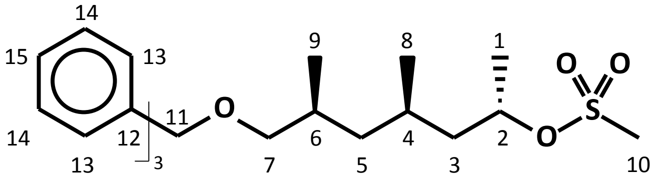

+ *ent*

A solution of 2.527 g (6.28 mmol) **18** and 3.0 mL (21.6 mmol) triethylamine in 65 mL dry dichloromethane was cooled to −80 °C. A solution of 0.73 mL (9.39 mmol) methanesulfonic acid chloride in 10 mL dry dichloromethane was added dropwise and stirred at −80 °C for 2 h. The reaction was quenched by addition of wet solid sodium hydrogencarbonate, and subsequently, the mixture was warmed to room temperature and filtered over silica. The solvent was removed in vacuo, and the residue was purified by column chromatography on silica (hexane:ethyl acetate = 8:1 to 4:1) yielding 2.741 g (5.70 mmol, 90%) of *anti,syn*-2-(methanesulfonyloxy)-4,6-dimethyl-7-(triphenylmethyloxy)heptane as a slightly yellowish, viscose oil. After characterization by its mass spectrum the compound was immediately used in the next step.

MS (EI, 70 eV, direct): m/z (%) = 41 (4), 42 (1), 43 (3), 45 (1), 55 (6), 56 (1), 57 (1), 59 (1), 67 (1), 69 (12), 70 (2), 77 (3), 79 (2), 82 (1), 83 (6), 84 (1), 91 (1), 99 (1), 105 (15), 106 (1), 115 (1), 123 (1), 125 (2), 141 (2), 154 (2), 165 (21), 166 (4), 167 (1), 181 (1), 183 (19), 184 (3), 202 (1), 215 (1), 226 (1), 228 (2), 229 (1), 237 (2), 239 (2), 240 (1), 241 (4), 242 (3), 243

(100, Ph_3C^+), 244 (33), 245 (4), 258 (4), 259 (9), 260 (2), 403 (2, M^+-Ph), 404 (1), 480 (1, M^+).

***syn,syn*-2,4,6-Trimethyl-1-(triphenylmethyloxy)nonane (19)**

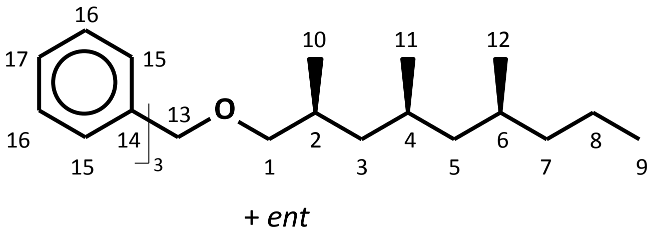

To a stirred mixture of 2.650 g (5.33 mmol) *anti,syn*-2-(methanesulfonyloxy)-4,6-dimethyl-7-(triphenylmethyloxy)heptane and 50 mg (0.26 mmol) P_2O_5-dried CuI in 30 mL dry THF, cooled to −90 °C, was dropwise added a solution of *n*-propylmagnesium bromide, freshly prepared from 291 mg (15.8 mmol) *n*-propyl bromide and 615 mg (25.3 mmol) magnesium. After stirring for 48 h at room temperature, the reaction mixture was worked-up the same way as described for **5**. Column chromatography on silica (hexane:ethyl acetate = 40:1 to 4:1) yielded 845 mg (1.97 mmol, 37%) of **19** as a colorless oil.

^{1}H-NMR, ^{1}H-^{1}H-COSY, HSQC, HMBC (400 MHz, $CDCl_3$): δ [ppm] = 0.78 (d, $^3J_{H-H}$=6.6 Hz, 3H, CH_3 C12), 0.79 (d, $^3J_{H-H}$=6.6 Hz, 3H, CH_3 C11), 0.84-0.92/1.16-1.24 (2m, 2H, CH_2 C5), 0.85-0.91/1.27-1.33 (2m, 2H, CH_2 C3), 0.88 (t, $^3J_{H-H}$=6.4 Hz, 3H, CH_3 C9), 0.94-1.00/1.25-1.32 (2m, 2H, CH_2 C5), 0.97 (d, $^3J_{H-H}$=6.8 Hz, 3H, CH_3 C10), 1.46-1.57 (m, 1H, CH C6), 1.54-1.64 (m, 1H, CH C4), 1.77-1.87 (m, 1H, CH C2), 2.82/2.97 (2dd, $^2J_{H-H}$=8.7, $^3J_{H-H}$=6.69/5.05 Hz, 2H, CH_2 C1), 7.21-7.26 (m, 3H, CH C17), 7.26-7.30 (m, 6H, CH C16), 7.45 (br.d, $^3J_{H-H}$=7.6 Hz, 6H, CH C15).

^{13}C-NMR, HSQC, HMBC (101 MHz, $CDCl_3$): δ [ppm] = 14.57 (q, C9), 18.83 (q, C10), 20.11 (t, C8), 20.63 (q, C12), 20.97 (q, C11), 27.79 (d, C4), 29.94 (d, C6), 31.61 (d, C2), 39.05 (t, C7), 42.24 (t, C3), 45.55 (t, C5), 68.62 (t, C1), 85.94 (s, C13), 126.90 (d, C17), 128.09 (d, C16), 128.96 (d, C15), 144.76 (s, C14).

MS (EI, 70 eV, GC/MS DB-5ms 30 m): m/z (%) = 41 (7), 43 (21), 55 (5), 57 (9), 69 (3), 71 (7), 77 (5), 85 (3), 105 (23), 111 (2), 154 (3), 165 (35), 166 (7), 167 (2), 183 (30), 184 (4), 185 (8), 215 (3), 228 (5), 239 (3), 241 (4), 242 (2), 243 (100), 244 (35), 245 (5), 259 (1), 260 (1), 351 (2), 352 (1), 428 (0.3, M^+).

***syn,syn*-2,4,6-Trimethylnonan-1-ol (20)**

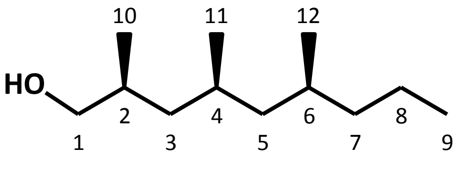

+ *ent*

Deprotection of 362 mg (1.97 mmol) of **19** was carried out with 76 mg (0.4 mmol) *p*-toluenesulfonic acid in 15 mL of a 15:20:1 mixture of MeOH, THF, and water under stirring at room temperature. After two hours, 4 mL of saturated aqueous sodium hydrogencarbonate was added. The mixture was concentrated in vacuo, and the residue was taken up with 20 mL diethyl ether. Work-up was carried out as described for the synthesis of **3**. Column chromatography on silica (hexane:ethyl acetate = 10:1) yielded 319 mg (1.71 mmol, 87%) of **20** as a colorless oil.

^{1}H-NMR, ^{1}H-^{1}H-COSY, HSQC, HMBC (400 MHz, $CDCl_3$): δ [ppm] = 0.84 (d, $^3J_{H\text{-}H}$=6.6 Hz, 3H, CH_3 C12), 0.85-0.94/1.17-1.26 (2m, 2H, CH_2 C5), 0.86-0.95/1.26-1.35 (2m, 2H, CH_2 C3), 0.87 (d, $^3J_{H\text{-}H}$=6.8 Hz, 3H, CH_3 C11), 0.88 (t, $^3J_{H\text{-}H}$=6.2 Hz, 3H, CH_3 C9), 0.93 (d, $^3J_{H\text{-}H}$=6.6 Hz, 3H, CH_3 C10), 0.97-1.06/1.24-1.34 (2m, 2H, CH_2 C7), 1.22-1.41 (m, 2H, CH_2 C8), 1.45-1.56 (m, 1H, CH C6), 1.53-1.61 (m, 1H, CH C4), 1.67-1.78 (m, 1H, CH C2), 3.38/3.54 (2dd, $^2J_{H\text{-}H}$=10.5, $^3J_{H\text{-}H}$=7.0/5.1 Hz, 2H, CH_2 C1).

^{13}C-NMR, HSQC, HMBC (101 MHz, $CDCl_3$): δ [ppm] = 14.42 (q, C9) 17.54 (q, C10) 19.94 (t, C8) 20.43 (q, C12) 20.92 (q, C11) 27.54 (d, C4) 29.75 (d, C6) 33.10 (d, C2) 38.84 (t, C7) 41.27 (t, C3) 45.18 (t, C5) 68.29 (t, C1).

MS (EI, 70 eV, GC/MS DB-5ms 30 m): m/z (%) = 39 (12), 40 (2), 41 (57), 42 (17), 43 (100), 44 (4), 45 (9), 53 (5), 54 (2), 55 (79), 56 (27), 57 (92), 58 (7), 59 (8), 65 (1), 67 (5), 68 (21), 69 (64), 70 (63), 71 (51), 72 (3), 79 (1), 81 (4), 82 (4), 83 (76), 84 (27), 85 (23), 86 (2), 95 (1), 96 (2), 97 (13), 98 (5), 99 (8), 101 (5), 110 (2), 111 (26), 112 (6), 113 (2), 124 (1), 125 (24), 126 (11), 127 (1), 139 (0.5), 153 (1), 168 (0.4), 169 (0.1), 186 (0.1, M^+).

***syn*,*syn*-2,4,6-Trimethylnonanal (21)**

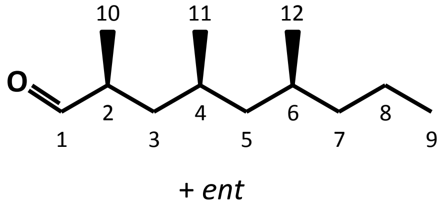

+ *ent*

The oxidation of **20** to the corresponding aldehyde followed the same procedure as that of **12** to **13** by treatment of 307 mg (1.65 mmol) of **20** in 15 mL dichloromethane with 993 mg (2.4 mmol) pyridinium dichromate. Work-up and column chromatography on silica yielded 219 mg (1.19 mmol, 72%) of **21** as a colorless oil.

^{1}H-NMR, ^{1}H-^{1}H-COSY, HSQC, HMBC (400 MHz, $CDCl_3$): δ [ppm] = 0.84 (d, $^3J_{H\text{-}H}$=6.5 Hz, 3H, CH_3 C12), 0.88 (t, $^3J_{H\text{-}H}$=7.2 Hz, 3H, CH_3 C9), 0.88 (d, $^3J_{H\text{-}H}$=6.5 Hz, 3H, CH_3 C11),

0.98-1.05 (m, 2H, CH_2 C5), 1.08 (d, $^{3}J_{H-H}$=6.8 Hz, 3H, CH_3 C10), 1.08/1.72 (2ddd, $^{2}J_{H-H}$=13.8, $^{3}J_{H-H}$=8.3, 6.0/8.3, 5.5 Hz, 2H, CH_2 C3), 1.16-1.39 (m, 2H, CH_2 C7), 1.22-1.32 (m, 2H, CH_2 C5), 1.30-1.39 (m, 2H, CH_2 C8), 1.46-1.55 (m, 1H, CH C6), 1.53-1.62 (m, 1H, CH C4), 2.45 (dqdd, $^{3}J_{H-H}$=8.3, 6.8, 6.0, 2.5 Hz, 1H, CH C2), 9.58 (d, $^{3}J_{H-H}$=2.5 Hz, 1H, CHO C1).

^{13}C-NMR, HSQC, HMBC (101 MHz, $CDCl_3$): δ [ppm] = 13.39 (q, C9), 14.54 (q, C10), 20.03 (q, C12), 20.12 (t, C8), 20.18 (q, C11), 27.62 (d, C4), 29.81 (d, C6), 38.65 (t, C3), 39.28 (t, C7), 44.39 (d, C2), 45.20 (t, C5), 205.61 (d, C1).

MS (EI, 70 eV, GC/MS DB-5ms 30 m): m/z (%) = 39 (16), 40 (3), 41 (56), 42 (13), 43 (100), 44 (3), 45 (1), 53 (6), 54 (2), 55 (39), 56 (10), 57 (65), 58 (51), 59 (3), 65 (1), 67 (6), 68 (3), 69 (25), 70 (21), 71 (84), 72 (9), 77 (1), 79 (2), 81 (12), 82 (5), 83 (24), 84 (6), 85 (26), 86 (2), 95 (10), 96 (6), 97 (4), 99 (4), 109 (3), 111 (7), 112 (1), 113 (1), 123 (9), 124 (2), 126 (22), 127 (10), 128 (1), 141 (2), 151 (1), 153 (0.4), 166 (0.2), 167 (0.2), 184 (0.2, M+).

(2*E*,4*E*/*Z*)-*syn*,*syn*-4,6,8,10-Tetramethyltrideca-2,4-diene (22)

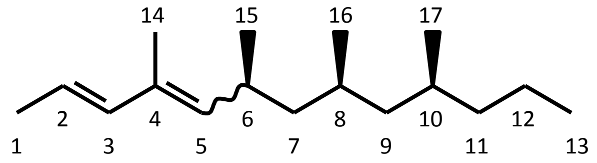

+ *ent*

The transformation of **21** into a mixture of the (2*E*,4*E*/4*Z*)-isomers of *syn*,*syn*-4,6,8,10-tetramethyltrideca-2,4-diene followed the way similar to the chain elongation of **7** to **8**. The Wittig olefination was carried out in 20 mL dry THF, using 719 mg (1.75 mmol) (*E*)-1-methylbut-2-enyltriphenylphosphonium bromide [2] and 160 mg (1.84 mmol) LiBr. After deprotonation with 0.50 mL (0.8 mmol) 1.6 M *n*-BuLi solution in hexane, 81 mg (0.44 mmol) of **21**, dissolved in 5 mL dry THF were dropwise added at −40 °C. The reaction mixture was stirred overnight and warmed up to room temperature. Work-up and column chromatography on silica using pentane as the eluent yielded 73 mg (0.31 mmol, 70%) of (2*E*,4*Z*/*E*)-*syn*,*syn*-4,6,8,10-tetramethyltrideca-2,4-diene (**22**) as a colorless oil.

Isomeric ratio by GC/MS: (4*Z*) : (4*E*) = 1 : 3 (order of elution).

(4*E*)-22

^{1}H-NMR, ^{1}H-^{1}H-COSY, HSQC, HMBC (400 MHz, $CDCl_3$): δ [ppm] = 0.81 (d, $^{3}J_{H-H}$=6.6 Hz, 6H, 2× CH_3 C16 C17), 0.86-0.96/1.10-1.21 (2m, 2H, CH_2 C9), 0.87 (t, $^{3}J_{H-H}$=7.1 Hz, 3H, CH_3 C13), 0.93 (d, $^{3}J_{H-H}$=6.6 Hz, 3H, CH_3 C15), 0.95-1.07/1.20-1.32 (2m, 2H, CH_2 C7), 0.98-

1.07/1.19-1.28 (2m, 2H, CH_2 C11), 1.21-1.40 (m, 2H, CH_2 C12), 1.39-1.50 (m, 1H, CH C8), 1.43-1.55 (m, 1H, CH C10), 1.74 (d, $^4J_{H\text{-}H}$=1.3 Hz, 3H, CH_3 C14), 1.76 (dd, $^3J_{H\text{-}H}$=6.6, $^4J_{H\text{-}H}$=1.5 Hz, 3H, CH_3 C1), 2.50-2.64 (m, 1H, CH C6), 5.07 (br.d, $^3J_{H\text{-}H}$=9.6 Hz, 1H, CH C5), 5.56 (dq, $^3J_{(E)H\text{-}H}$=15.4, $^3J_{H\text{-}H}$=6.5 Hz, 1H, CH C2), 6.07 (dq, $^3J_{(E)H\text{-}H}$=15.4, $^4J_{H\text{-}H}$=1.8 Hz, 1H, CH C3).

^{13}C-NMR, HSQC, HMBC (101 MHz, $CDCl_3$): δ [ppm] = 12.82 (q, C14), 14.56 (q, C13), 18.34 (q, C1), 20.18 (t, C12), 20.19 (q, C17), 20.63 (q, C16), 22.02 (q, C15), 28.13 (d, C8), 29.81 (d, C10), 30.19 (d, C6), 39.60 (t, C11), 45.26 (t, C7), 45.93 (t, C9), 121.94 (d, C2), 132.08 (s, C4), 136.51 (d, C3), 137.23 (d, C5).

MS (EI, 70 eV, GC/MS DB-5ms 30 m): m/z (%) = 39 (7), 41 (30), 42 (4), 43 (32), 53 (5), 55 (18), 56 (3), 57 (10), 65 (4), 67 (34), 68 (3), 69 (33), 70 (4), 71 (8), 77 (7), 79 (11), 81 (18), 82 (43), 83 (11), 85 (2), 91 (8), 93 (8), 94 (3), 95 (28), 96 (5), 97 (8), 105 (2), 107 (6), 108 (2), 109 (100), 110 (15), 111 (8), 123 (10), 124 (3), 151 (4), 152 (2), 153 (3), 177 (1), 193 (1), 220 (1), 236 (5, M^+).

(4Z)-22

^{1}H-NMR, ^{1}H-^{1}H-COSY, HSQC, HMBC (400 MHz, $CDCl_3$): δ [ppm] = 0.81/0.82 (2d, $^3J_{H\text{-}H}$=6.6/6.6 Hz, 6H, 2× CH_3 C16 C17), 0.86-0.96/1.10-1.21 (2m, 2H, CH_2 C9), 0.87 (t, $^3J_{H\text{-}H}$=7.2 Hz, 3H, CH_3 C13), 0.93 (d, $^3J_{H\text{-}H}$=6.6 Hz, 3H, CH_3 C15), 0.95-1.07/1.20-1.32 (2m, 2H, CH_2 C7), 0.98-1.07/1.19-1.28 (2m, 2H, CH_2 C11), 1.21-1.40 (m, 2H, CH_2 C12), 1.40-1.50 (m, 1H, CH C8), 1.43-1.54 (m, 1H, CH C10), 1.78 (d, $^4J_{H\text{-}H}$=1.3 Hz, 3H, CH_3 C14), 1.80 (dd, $^3J_{H\text{-}H}$=6.6, $^4J_{H\text{-}H}$=1.3 Hz, 3H, CH_3 C1), 2.64-2.78 (m, 1H, CH C6), 4.94 (br.d, $^3J_{H\text{-}H}$=9.6 Hz, 1H, CH C5), 5.68 (dq, $^3J_{(E)H\text{-}H}$=15.4, $^3J_{H\text{-}H}$=6.53 Hz, 1H, CH C2), 6.45 (br.d, $^3J_{(E)H\text{-}H}$=15.4 Hz, 1H, CH C3).

^{13}C-NMR, HSQC, HMBC (101 MHz, $CDCl_3$): δ [ppm] = 14.56 (q, C13), 18.82 (q, C1), 20.18 (t, C12), 20.57 (q, C14), 20.77 (q, C17), 20.89 (q, C16), 22.36 (q, C15), 28.03 (d, C8), 29.27 (d, C6), 29.82 (d, C10), 39.60 (t, C11), 45.40 (t, C7), 45.87 (t, C9), 124.96 (d, C2), 128.99 (d, C3), 131.53 (s, C4), 135.57 (d, C5).

MS (EI, 70 eV): m/z (%) = 39 (8), 41 (35), 42 (5), 43 (37), 53 (7), 55 (22), 56 (4), 57 (10), 65 (5), 67 (42), 68 (4), 69 (39), 70 (6), 71 (9), 77 (8), 78 (2), 79 (13), 80 (2), 81 (21), 82 (48), 83 (12), 84 (2), 85 (2), 91 (10), 93 (9), 94 (3), 95 (31), 96 (6), 97 (9), 105 (3), 107 (7), 108 (2), 109 (100), 110 (17), 111 (9), 123 (12), 124 (4), 137 (3), 151 (6), 152 (4), 153 (5), 193 (2), 194 (2), 221 (1), 236 (5, M^+).

Methyl (2*E*,4*E*/*Z*)-*syn*,*syn*-4,6,8,10-tetramethyltrideca-2,4-dienoate

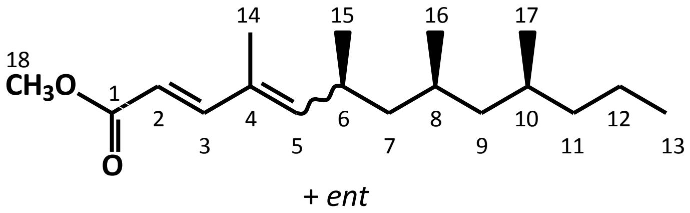

A solution of 254 mg (1.02 mmol) diethyl [(1/2*E*/*Z*)-3-methoxycarbonyl-1-methylprop-1/2-en-1-yl] phosphonate [5] and 443 mg (5.1 mmol) LiBr in 15 ml dry THF was cooled to −90 °C. After the addition of 0.8 mL (0.80 mmol) 1 M sodium hexamethyldisilazide in dry THF, the mixture was continued to stir for 1 h at −70 °C. Subsequently, 98 mg (053 mmol) of **21,** dissolved in 5 mL in dry THF, was added and slowly warmed to room temperature. After stirring overnight, 6 mL of a saturated aqueous solution of ammonium chloride was added. After the addition of 20 mL diethyl ether, the organic layer was separated, and the aqueous phase was extracted with another 5 mL of diethyl ether. The combined organic solutions were worked-up as described for **3**. Column chromatography on silica (hexane:ethyl acetate = 30:1) afforded 64 mg (0.23 mmol, 48%) of methyl (2*E*,4*E*/*Z*)-*syn*,*syn*-4,6,8,10-tetramethyltrideca-2,4-dienoate as a colorless oil.

Isomeric ratio by GC/MS: (4*Z*) : (4*E*) = 55 : 45 (order of elution).

(4*E*)

^{1}H-NMR, ^{1}H-^{1}H-COSY, HSQC, HMBC (400 MHz, $CDCl_3$): δ [ppm] = 0.80 (d, $^{3}J_{H\text{-}H}$=6.5 Hz, 3H, CH_3 C17), 0.87 (t, $^{3}J_{H\text{-}H}$=7.3 Hz, 3H, CH_3 C13), 0.88 (d, $^{3}J_{H\text{-}H}$=6.5 Hz, 3H, CH_3 C16), 0.90-0.97/1.14-1.23 (2m, 2H, CH_2 C9), 0.97 (d, $^{3}J_{H\text{-}H}$=6.8 Hz, 3H, CH_3 C15), 0.99-1.08/1.18-1.28 (2m, 2H, CH_2 C11), 1.01-1.09/1.28-1.37 (2m, 2H, CH_2 C7), 1.24-1.36 (m, 2H, CH_2 C12), 1.37-1.43 (m, 1H, CH C8), 1.43-1.53 (m, 1H, CH C10),1.79 (d, $^{4}J_{H\text{-}H}$=1.3 Hz, 3H, CH_3 C14), 2.59-2.72 (m, 1H, CH C6), 3.75 (s, 3H, CH_3 C18), 5.63 (br.d, $^{3}J_{H\text{-}H}$=10.5 Hz, 1H, CH C5), 5.78 (br.d, $^{3}J_{(E)H\text{-}H}$=15.6 Hz, 1H, CH C2), 7.32 (br.d, $^{3}J_{H\text{-}H}$=15.6, $^{4}J_{(E)H\text{-}H}$=0.63 Hz, 1H, CH C3).

^{13}C-NMR, HSQC, HMBC (101 MHz, $CDCl_3$): δ [ppm] = 12.15 (q, C14), 14.54 (q, C13), 20.19 (q, C16), 20.37 (t, C12), 20.54 (q, C17), 21.35 (q, C15), 28.28 (d, C8), 29.78 (d, C10), 31.04 (d, C6), 39.51 (t, C11), 44.79 (t, C7), 45.85 (t, C9), 51.58 (q, C18), 115.18 (d, C2), 131.70 (s, C4), 146.57 (d, C3), 150.36 (d, C5), 167.98 (s, C1).

MS (EI, 70 eV, GC/MS DB-5ms 30 m): m/z (%) = 39 (11), 40 (3), 41 (59), 42 (10), 43 (104), 44 (4), 45 (2), 51 (3), 52 (2), 53 (13), 54 (3), 55 (42), 56 (14), 57 (35), 58 (2), 59 (22), 65 (8), 66 (3), 67 (19), 68 (4), 69 (67), 70 (19), 71 (29), 72 (2), 77 (27), 78 (5), 79 (34), 80 (5),

81 (11), 82 (6), 83 (15), 84 (12), 85 (15), 87 (1), 91 (30), 92 (5), 93 (79), 94 (20), 95 (44), 96 (5), 97 (15), 98 (3), 103 (1), 105 (6), 106 (2), 107 (29), 108 (10), 109 (26), 110 (7), 111 (33), 112 (18), 113 (3), 114 (11), 115 (2), 119 (3), 120 (1), 121 (22), 122 (11), 123 (12), 124 (4), 125 (64), 126 (8), 127 (100), 128 (9), 129 (1), 133 (1), 135 (9), 136 (5), 137 (7), 138 (1), 139 (8), 140 (2), 141 (2), 149 (3), 151 (6), 152 (3), 153 (30), 154 (12), 155 (3), 156 (2), 163 (3), 164 (1), 165 (2), 166 (1), 167 (8), 168 (3), 169 (2), 177 (3), 181 (1), 195 (3), 196 (2), 197 (2), 205 (1), 206 (2), 209 (2), 237 (1), 249 (4), 280 (2, M^+).

(4*Z*)

^{1}H-NMR, ^{1}H-^{1}H-COSY, HSQC, HMBC (400 MHz, $CDCl_3$): δ [ppm] = 0.81 (d, $^3J_{H\text{-}H}$=6.5 Hz, 3H, CH_3 C17), 0.82 (d, $^3J_{H\text{-}H}$=6.5 Hz, 3H, CH_3 C16), 0.87 (t, $^3J_{H\text{-}H}$=7.3 Hz, 3H, CH_3 C13), 0.90-0.97/1.14-1.23 (2m, 2H, CH_2 C9), 0.97 (d, $^3J_{H\text{-}H}$=6.8 Hz, 3H, CH_3 C15), 0.99-1.08/1.18-1.28 (2m, 2H, CH_2 C11), 1.00-1.08/1.23-1.32 (2m, 2H, CH_2 C7), 1.24-1.36 (m, 2H, CH_2 C12), 1.37-1.43 (m, 1H, CH C8), 1.43-1.53 (m, 1H, CH C10),1.85 (d, $^4J_{H\text{-}H}$=1.3 Hz, 3H, CH_3 C14), 2.80-2.93 (m, 1H, CH C6), 3.76 (s, 3H, CH_3 C18), 5.46 (d, $^3J_{H\text{-}H}$=10.8 Hz, 1H, CH C5), 5.88 (br.d, $^3J_{(E)H\text{-}H}$=15.6 Hz, 1H, CH C2), 7.76 (br.d, $^3J_{(E)H\text{-}H}$=15.6 Hz, 1H, CH C3)

^{13}C-NMR, HSQC, HMBC (101 MHz, $CDCl_3$): δ [ppm] = 14.54 (q, C13), 20.12 (q, C14), 20.21 (q, C16), 20.37 (t, C12), 20.53 (q, C17), 21.58 (q, C15), 27.66 (d, C8), 29.78 (d, C10), 30.18 (d, C6), 39.51 (t, C11), 44.93 (t, C7), 45.80 (t, C9), 51.65 (q, C18), 117.79 (d, C2), 129.83 (s, C4), 141.85 (d, C3), 149.02 (d, C5), 167.83 (s, C1).

MS (EI, 70 eV, GC/MS DB-5ms 30 m): m/z (%) = 39 (9), 40 (3), 41 (47), 42 (8), 43 (83), 44 (3), 45 (2), 51 (2), 52 (2), 53 (11), 54 (2), 55 (39), 56 (14), 57 (32), 58 (2), 59 (19), 65 (7), 66 (3), 67 (18), 68 (4), 69 (63), 70 (19), 71 (26), 72 (2), 77 (26), 78 (5), 79 (31), 80 (5), 81 (11), 82 (6), 83 (16), 84 (14), 85 (16), 86 (1), 87 (1), 91 (29), 92 (5), 93 (82), 94 (21), 95 (47), 96 (5), 97 (15), 98 (3), 99 (1), 103 (1), 105 (6), 106 (2), 107 (28), 108 (10), 109 (25), 110 (8), 111 (32), 112 (17), 113 (3), 114 (11), 115 (2), 119 (3), 120 (1), 121 (21), 122 (11), 123 (12), 124 (4), 125 (54), 126 (7), 127 (100), 128 (9), 129 (1), 133 (1), 135 (8), 136 (5), 137 (7), 138 (1), 139 (7), 140 (2), 141 (2), 149 (2), 151 (5), 152 (3), 153 (25), 154 (11), 155 (4), 156 (2), 163 (3), 164 (1), 165 (2), 166 (1), 167 (8), 168 (3), 169 (2), 177 (3), 195 (2), 196 (2), 197 (2), 205 (1), 206 (2), 209 (1), 237 (1), 249 (2), 280 (1, M^+).

(2*E*,4*E*/*Z*)-*syn,syn*-4,6,8,10-Tetramethyltrideca-2,4-dien-1-ol (23)

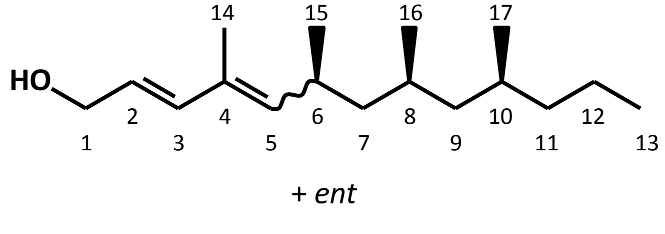

A solution of 52 mg (0.19 mmol) of methyl (2*E*,4*E/Z*)-*syn,syn*-4,6,8,10-tetramethyltrideca-2,4-dienoate in 6 mL dry dichloromethane was cooled to −80 °C. After dropwise addition of 0.5 mL (0.5 mmol) 1 M DIBAlH in heptane, the mixture was continued to stir for 1.5 h at −30 °C. Subsequently, 0.2 mL (84.9 mmol) methanol in 2 mL dichloromethane was added, and the solvent was removed in vacuo. The residue was taken up in 20 mL diethyl ether. The organic solution was stirred with 5 mL of a 1:1 mixture of saturated, aqueous solution of ammonium chloride and 2 N HCl. The organic layer was separated and worked-up as for **3**. Colum chromatography on silica (hexane:ethyl acetate = 10:1) afforded 44 mg (0.174 mmol, 92%) of (2*E*,4*E*)- and (2*E*,4*Z*)-*syn,syn*-4,6,8,10-tetramethyltrideca-2,4-dien-1-ol (**23**) as a colorless oil.

Isomeric ratios by GC/MS: (2*Z*) : (2*E*) = 48 : 52; (2*E*,4*Z*)-*syn,syn* : (2*E*,4*Z*)-*syn,anti* + (2*E*,4*Z*)-*anti,syn* : (2*E*,4*Z*)-*anti,anti* : (2*E*,4*E*)-*syn,syn* + (2*E*,4*E*)-*syn,anti* : (2*E*,4*E*)-*anti,syn* : (2*E*,4*E*)-*anti,anti* = 44 : 2 : 2 : 49 : 2 : 1 (order of elution).

(4*E*)

^{1}H-NMR, ^{1}H-^{1}H-COSY, HSQC, HMBC (400 MHz, $CDCl_3$): δ [ppm] = 0.80 (d, $^3J_{H\text{-}H}$=6.7 Hz, 3H, CH_3 C17), 0.86-0.95/1.11-1.21 (2m, 2H, CH_2 C9), 0.87 (t, $^3J_{H\text{-}H}$=7.1 Hz, 3H, CH_3 C13), 0.92 (d, $^3J_{H\text{-}H}$=6.7 Hz, 3H, CH_3 C16), 0.93 (d, $^3J_{H\text{-}H}$=6.7 Hz, 3H, CH_3 C15), 0.98-1.08/1.18-1.28 (2m, 2H, CH_2 C11), 1.19-1.42 (m, 2H, CH_2 C12), 1.23-1.27/1.28-1.33 (2m, 2H, CH_2 C7), 1.35-1.49 (m, 1H, CH C8), 1.42-1.57 (m, 1H, CH C10), 1.77 (d, $^4J_{H\text{-}H}$=0.9 Hz, 3H, CH_3 C14), 2.52-2.65 (m, 1H, CH C6), 4.20 (dd, $^3J_{H\text{-}H}$=6.2, $^4J_{H\text{-}H}$=0.9 Hz, 2H, CH_2 C1), 5.22 (d, $^3J_{H\text{-}H}$=9.7 Hz, 1H, CH C5), 5.71 (dt, $^3J_{(E)H\text{-}H}$=15.7, $^3J_{H\text{-}H}$=6.2 Hz, 1H, CH C2), 6.25 (d, $^3J_{(E)H\text{-}H}$=15.7 Hz, 1H, CH C3).

^{13}C-NMR, HSQC, HMBC (126 MHz, $CDCl_3$): δ [ppm] = 13.02 (q, C14), 14.56 (q, C13), 18.99 (q, C16), 20.16 (t, C12), 20.58 (q, C17), 21.83 (q, C15), 28.15 (d, C8), 29.78 (d, C10), 30.38 (d, C6), 39.56 (t, C11), 45.07 (t, C7), 45.88 (t, C9), 64.39 (t, C1), 125.08 (d, C2), 131.47 (s, C4), 137.35 (d, C3), 140.55 (d, C5).

MS (EI, 70 eV, GC/MS DB-5ms 30 m): m/z (%) = 39 (9), 40 (2), 41 (53), 42 (7), 43 (100), 44 (3), 45 (3), 51 (2), 53 (10), 54 (2), 55 (58), 56 (14), 57 (32), 58 (2), 65 (5), 66 (2), 67 (16), 68 (4), 69 (79), 70 (25), 71 (23), 72 (1), 77 (11), 78 (2), 79 (21), 80 (8), 81 (24), 82 (12), 83 (30), 84 (10), 85 (11), 91 (22), 92 (3), 93 (22), 94 (5), 95 (67), 96 (9), 97 (26), 98 (8), 99 (1), 105 (9), 106 (2), 107 (65), 108 (16), 109 (24), 110 (5), 111 (17), 112 (2), 113 (1), 121 (8), 122 (3), 123 (13), 124 (5), 125 (11), 126 (4), 129 (2), 135 (2), 137 (4), 138 (1), 139 (4), 149 (3), 151 (4), 152 (1), 153 (4), 163 (3), 165 (1), 168 (1), 191 (1), 221 (3), 252 (2, M^+).

(4*Z*)

^{1}H-NMR, ^{1}H-^{1}H-COSY, HSQC, HMBC (400 MHz, $CDCl_3$): δ [ppm] = 0.80 (d, $^3J_{H\text{-}H}$=6.5 Hz, 3H, CH_3 C17), 0.85 (d, $^3J_{H\text{-}H}$=6.5 Hz, 3H, CH_3 C16), 0.86-0.95/1.11-1.21 (2m, 2H, CH_2 C9), 0.88 (t, $^3J_{H\text{-}H}$=7.0 Hz, 3H, CH_3 C13), 0.93 (d, $^3J_{H\text{-}H}$=6.5 Hz, 3H, CH_3 C15), 0.98-1.08/1.18-1.28 (2m, 2H, CH_2 C11), 1.19-1.24/1.25-1.29 (2m, 2H, CH_2 C7), 1.19-1.42 (m, 2H, CH_2 C12), 1.35-1.49 (m, 1H, CH C8), 1.42-1.57 (m, 1H, CH C10), 1.82 (d, $^3J_{H\text{-}H}$=0.9 Hz, 3H, CH_3 C14), 2.66-2.78 (m, 1H, CH C6), 4.24 (d, $^3J_{H\text{-}H}$=6.0 Hz, 2H, CH_2 C1), 5.10 (d, $^3J_{H\text{-}H}$=9.8 Hz, 1H, CH C5), 5.82 (dt, $^3J_{(E)H\text{-}H}$=15.6, $^3J_{H\text{-}H}$=6.0 Hz, 1H, CH C2), 6.66 (d, $^3J_{(E)H\text{-}H}$=15.6 Hz, 1H, CH C3).

^{13}C-NMR, HSQC, HMBC (126 MHz, $CDCl_3$): δ [ppm] = 14.56 (q, C13), 20.16 (t, C12), 20.61 (q, C16), 20.72 (q, C17), 20.76 (q, C14), 22.27 (q, C15), 28.03 (d, C8), 29.48 (d, C6), 29.78 (d, C10), 39.56 (t, C11), 45.19 (t, C7), 45.82 (t, C9), 64.22 (t, C1), 128.00 (d, C2), 129.19 (d, C3), 129.85 (s, C4), 138.67 (d, C5).

MS (EI, 70 eV, GC/MS DB-5ms 30 m): m/z (%) = 39 (10), 40 (2), 41 (53), 42 (7), 43 (100), 44 (3), 45 (3), 51 (2), 53 (10), 54 (2), 55 (59), 56 (14), 57 (31), 58 (2), 65 (5), 66 (2), 67 (17), 68 (4), 69 (81), 70 (27), 71 (21), 72 (1), 77 (11), 78 (2), 79 (21), 80 (7), 81 (24), 82 (13), 83 (28), 84 (11), 85 (11), 91 (23), 92 (3), 93 (23), 94 (5), 95 (68), 96 (9), 97 (24), 98 (8), 99 (1), 105 (9), 106 (2), 107 (58), 108 (13), 109 (25), 110 (5), 111 (16), 112 (2), 121 (8), 122 (3), 123 (13), 124 (6), 125 (11), 126 (4), 129 (2), 135 (2), 137 (4), 138 (1), 139 (5), 140 (1), 149 (3), 150 (1), 151 (4), 152 (1), 153 (4), 163 (3), 165 (1), 167 (1), 168 (2), 191 (1), 221 (3), 221 (3), 222 (0.5), 234 (0.4), 252 (1, M^+).

(2*E*,4*Z*)-4,6,8,10-tetramethyltrideca-2,4-dien-1-ol (23a)

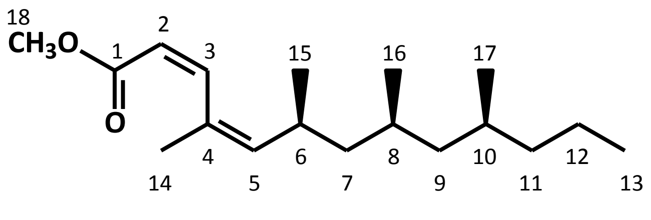

+ *ent*

A (4*Z*)-selective synthesis of an isomeric mixture of methyl (2*E*,4*Z*)-4,6,8,10-tetramethyltrideca-2,4-dienoate was carried out the same way as described for that of the (2*E*,4*E*/*Z*)-*syn*,*syn*-isomers, however, without the addition of lithium bromide. A solution of 193 mg (0.77 mmol) diethyl [(1/2*E*/*Z*)-3-methoxycarbonyl-1-methyl-1/2-en-1-yl]phosphonate in 10 mL dry THF was cooled to −90 °C. After the addition of 520 µL (0.32 mmol) 1 M sodium hexamethyldisilazide in dry THF, the mixture was continued to stir for 1 h at −70 °C.

Subsequently, 64 mg (0.35 mmol) of **13**, dissolved in 5 mL dry THF, was added and slowly warmed to room temperature. Work-up and purification of the reaction mixture as described for the synthesis of (2*E*,4*E*/*Z*)-*syn*,*syn*-4,6,8,10-tetramethyltrideca-2,4-dienoate yielded 36 mg (0.13 mmol, 37%) of the desired mixture of stereoisomers as a colorless oil.

Isomeric ratios by GC/MS: (4*Z*) : (4*E*) = 87 : 13; 4*Z*,*syn*,*syn* : 4*Z*,*anti*,*syn* : 4*Z*,*syn*,*anti* : 4*Z*,*anti*,*anti* = 11 : 51 : 7 : 31 (order of elution).

(2*Z*,4*Z*)-*anti*,*syn*-23

MS (EI, 70 eV, GC/MS DB-5ms 30 m): m/z (%) = 39 (12), 40 (3), 41 (59), 42 (9), 43 (100), 44 (4), 45 (2), 51 (2), 52 (1), 53 (10), 54 (2), 55 (29), 56 (9), 57 (21), 58 (1), 59 (14), 65 (5), 66 (2), 67 (12), 68 (2), 69 (43), 70 (13), 71 (19), 72 (1), 77 (19), 78 (3), 79 (23), 80 (4), 81 (9), 82 (5), 83 (14), 84 (13), 85 (14), 87 (1), 91 (22), 92 (3), 93 (66), 94 (16), 95 (38), 96 (4), 97 (12), 98 (3), 105 (4), 106 (1), 107 (19), 108 (6), 109 (17), 110 (5), 111 (20), 112 (11), 113 (2), 114 (6), 115 (1), 119 (2), 121 (12), 122 (6), 123 (7), 124 (2), 125 (30), 126 (4), 127 (53), 128 (5), 135 (5), 136 (4), 137 (5), 139 (5), 140 (1), 141 (1), 149 (2), 151 (4), 152 (2), 153 (19), 154 (8), 155 (3), 156 (1), 163 (2), 165 (2), 167 (6), 168 (2), 169 (2), 177 (2), 195 (2), 196 (2), 197 (2), 206 (2), 209 (1), 237 (1), 249 (2), 280 (2, M^+).

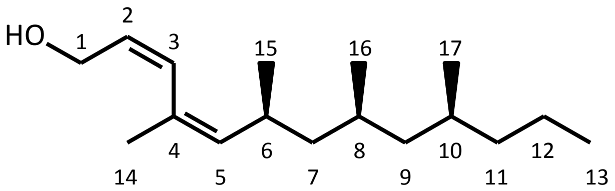

+ *ent*

A (4*Z*)-selective synthesis of an isomeric mixture of (2*E*,4*Z*)-4,6,8,10-tetramethyltrideca-2,4 dien-1-ol (23a) was carried out the same way as described for that of the mixture of the (2*E*,4*E*/*Z*)-*syn*,*syn*-isomers **23**. A solution of 31 mg (0.11 mmol) of methyl (2*E*,4*Z*)-4,6,8,10-tetramethyltrideca-2,4-dienoate in 5 mL dry dichloromethane was cooled to −80 °C. After dropwise addition of 0.28 mL (0.28 mmol) 1 M DIBAlH in heptane, the mixture was continued to stir for 1.5 h at −30 °C. Quenching, work-up, and purification as described for the synthesis of **23** afforded 23 mg (0.09 mmol, 82%) of the desired mixture **23a** as a colorless oil.

Isomeric ratios by GC/MS: (4*Z*) : (4*E*) = 61 : 39; 4*Z*,*syn*,*syn* : 4*Z*,*anti*,*syn* + 4*Z*,*syn*,*anti* : 4*Z*,*anti*,*anti* : 4*E*,*syn*,*syn* : 4*E*,*anti*,*syn* : 4*E*,*syn*,*anti* : 4*E*,*anti*,*anti* = 5 : 37 : 20 : 4 : 5 : 17 : 12 (order of elution).

(2*Z*,4*Z*)-*syn*,*anti*/*anti*,*syn*-23

MS (EI, 70 eV, GC/MS DB-5ms 30 m): m/z (%) = 38 (1), 39 (22), 40 (6), 41 (112), 42 (15), 43 (185), 44 (7), 45 (4), 50 (1), 51 (3), 52 (2), 53 (17), 54 (3), 55 (81), 56 (19), 57 (36), 58 (2), 59 (1), 63 (1), 65 (8), 66 (3), 67 (24), 68 (5), 69 (81), 70 (30), 71 (28), 72 (2), 77 (19), 78 (4), 79 (33), 80 (11), 81 (32), 82 (17), 83 (37), 84 (15), 85 (15), 86 (1), 87 (1), 91 (39), 92 (6), 93 (36), 94 (9), 95 (100), 96 (14), 97 (36), 98 (12), 99 (2), 103 (2), 104 (1), 105 (15), 106 (4), 107 (90), 108 (23), 109 (35), 110 (7), 111 (20), 112 (3), 113 (1), 115 (1), 117 (1), 119 (2), 120 (1), 121 (12), 122 (4), 123 (16), 124 (7), 125 (13), 126 (5), 127 (1), 129 (3), 133 (1), 135 (3), 136 (1), 137 (6), 138 (2), 139 (6), 140 (2), 141 (1), 149 (5), 150 (2), 151 (6), 152 (2), 153 (5), 154 (1), 163 (4), 164 (1), 165 (2), 166 (1), 167 (2), 168 (2), 191 (2), 193 (1), 219 (1), 221 (4), 222 (1), 234 (1), 252 (1, M^+).

Gas chromatograms

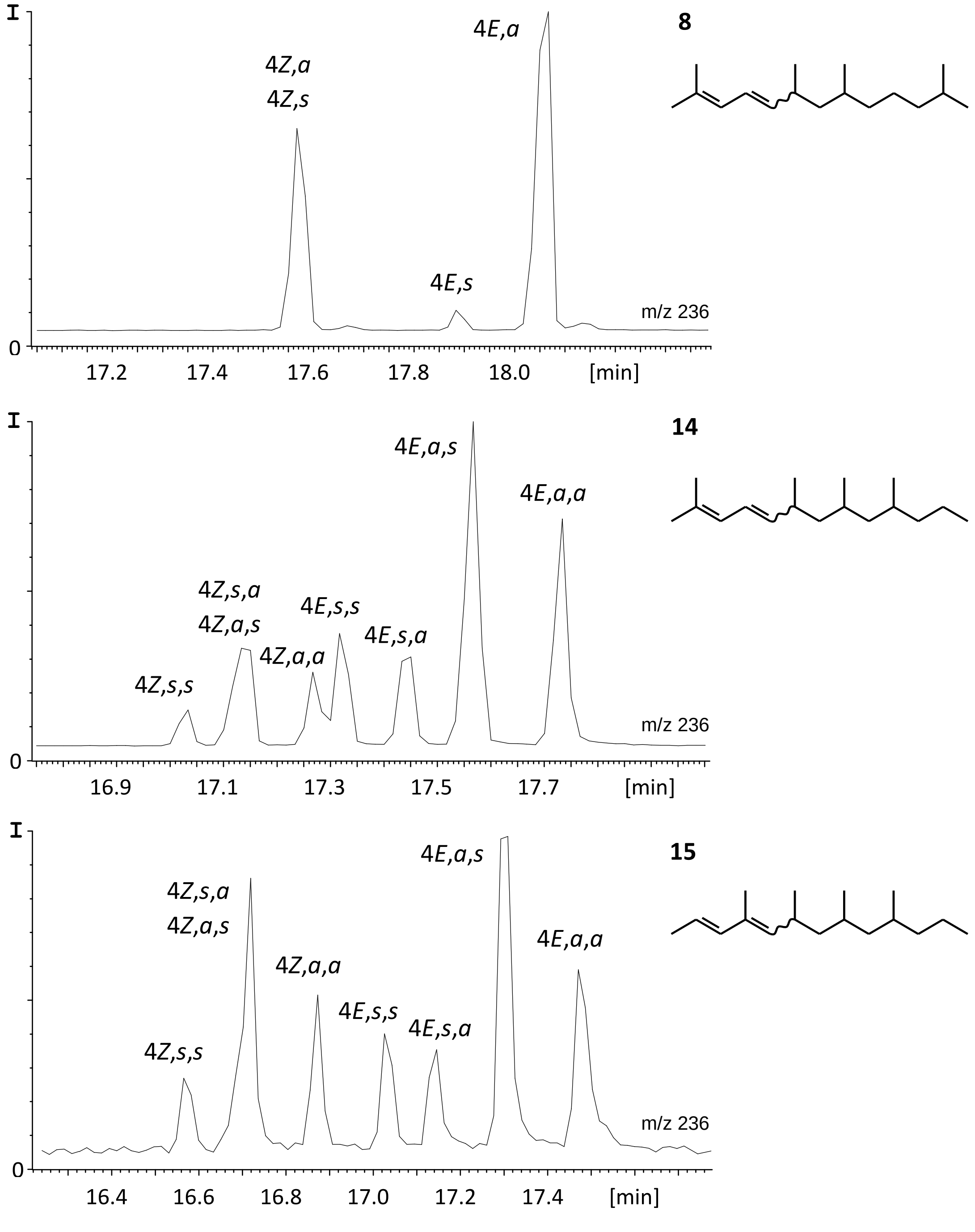

Figure S1: TIC-Chromatograms (m/z 236) of tetramethyltrideca-2,4-dienes **8**, **14,** and **15**; *a* = *anti*, *s* = *syn* (for conditions see general information).

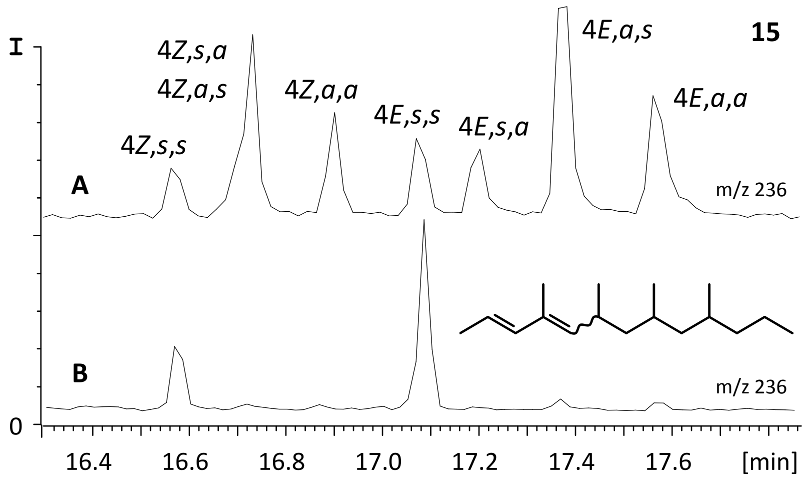

Figure S2: TIC-Chromatograms (m/z 236) of 4,6,8,10-tetramethyltrideca-2,4-diene **15**. **A:** mixture of all isomers of (2*E*,4*EZ*)-**15**, **B:** (2*E*,4*EZ*)-*syn*,*syn*-**15**.

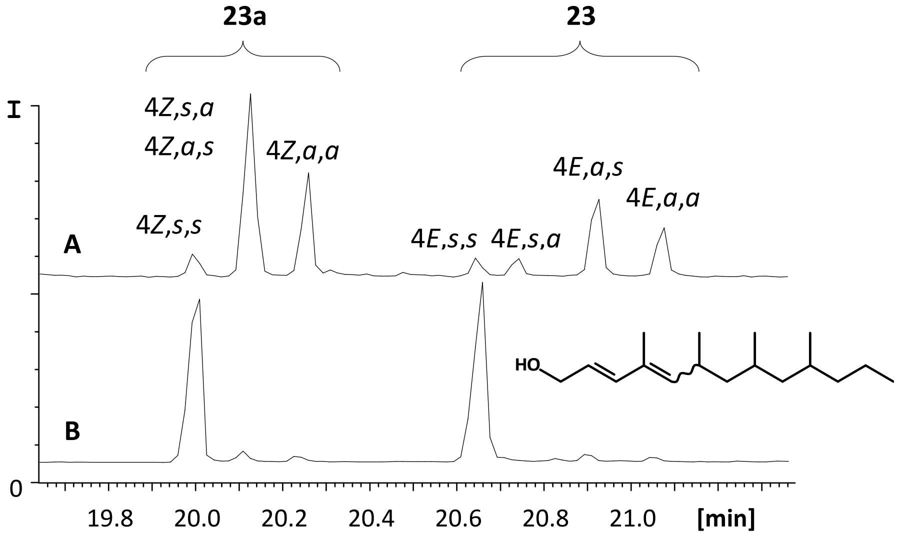

Figure S3: TIC-Chromatograms of 4,6,8,10-tetramethyltrideca-2,4-diene-1-ol. **A**: mixture of all (2*E*,4*EZ*)-isomers (**23** and **23a**), **B**: (2*E*,4*E*)-*syn*,*syn*-**23** and (2*E*,4*Z*)-*syn*,*syn*-**23a**.

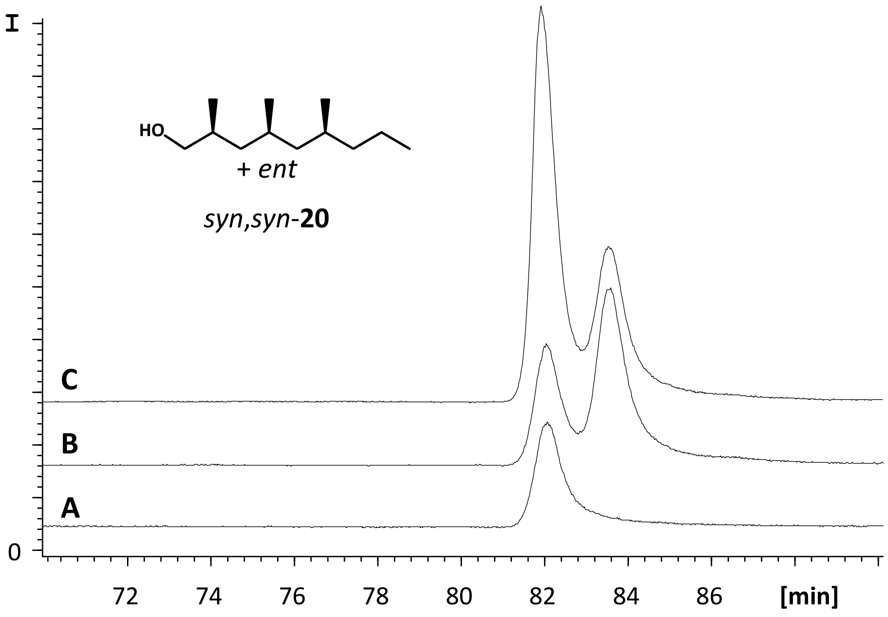

Figure S4: Enantioselective GC/MS-SIM-chromatogram (m/z 111, 125, 126) of syn,syn-2,4,6-trimethylnonan-1-ol (**20**). **A**: (2*R*,4*R*,6*R*)-**20**, **B**: racemate, **C**: mixture of **A** and **B**.

Index of NMR spectra

^{1}H-NMR spectrum (400 MHz, $CDCl_3$) and S34

^{13}C-PENDANT-NMR spectrum (126 MHz, $CDCl_3$) of *anti*-5-(*tert*-butyldimethylsilyloxy)-2,4-dimethylpentan-1-ol (**3**) S35

^{1}H-NMR spectrum (400 MHz, $CDCl_3$) and S36

^{13}C-PENDANT-NMR spectrum (101 MHz, $CDCl_3$) of *anti*-5-(*tert*-butyldimethylsilyloxy)-1-(*p*-toluenesulfonyloxy)-2,4-dimethylpentane (**4**) S37

^{1}H-NMR spectrum (400 MHz, $CDCl_3$) and S38

^{13}C-PENDANT-NMR spectrum (101 MHz, $CDCl_3$) of 2,4,8-trimethylnonan-1-ol (**6**) S39

^{1}H-NMR spectrum (400 MHz, $CDCl_3$) and S40

^{13}C-PENDANT-NMR spectrum (101 MHz, $CDCl_3$) of 2,4,8-trimethylnonanal (**7**) S41

^{1}H-NMR spectrum (500 MHz, $CDCl_3$) and S42

^{13}C-PENDANT-NMR spectrum (101 MHz, $CDCl_3$) of 2,6,8,12-tetramethyltrideca-2,4-diene (**8**) S43

^{1}H-NMR spectrum (400 MHz, $CDCl_3$) and S44

^{13}C-PENDANT-NMR spectrum (101 MHz, $CDCl_3$) of *anti*-5-benzyloxy-2,4-dimethylpentan-1-ol (**9**) S45

^{1}H-NMR spectrum (500 MHz, $CDCl_3$) and S46

^{13}C-PENDANT-NMR spectrum (126 MHz, $CDCl_3$) of *anti*-5-benzyloxy-2,4-dimethylpentanal (**10**) S47

^{1}H-NMR spectrum (400 MHz, $CDCl_3$) and S48

^{13}C-PENDANT-NMR spectrum (101 MHz, $CDCl_3$) of (5*E*/*Z*,7*E*)-*anti*-1-benzyloxy-2,4,6-trimethylnona-5,7-diene (**11**) S49

^{1}H-NMR spectrum (400 MHz, $CDCl_3$) and S50

^{13}C-PENDANT-NMR spectrum (126 MHz, $CDCl_3$) of (4*E*/*Z*)-2,6,8,10-tetramethyltrideca-2,4-diene (**14**) S51

^{1}H-NMR spectrum (400 MHz, $CDCl_3$) and S52

^{13}C-PENDANT-NMR spectrum (126 MHz, $CDCl_3$) of *syn,syn*-4,6-dimethyl-7-(triphenylmethyloxy)heptan-2-ol (**17**) S53

^{1}H-NMR spectrum (400 MHz, $CDCl_3$) and S54

^{13}C-PENDANT-NMR spectrum (101 MHz, $CDCl_3$) of *syn*-*syn*-2,4,6-trimethyl-1-(triphenylmethyloxy)nonane (**19**) S55

^{1}H-NMR spectrum (400 MHz, $CDCl_3$) and S56

^{13}C-PENDANT-NMR spectrum (101 MHz, $CDCl_3$) of *syn,syn*-2,4,6-trimethylnonan-1-ol (**20**) S57

^{1}H-NMR spectrum (400 MHz, $CDCl_3$) and S58

^{13}C-PENDANT-NMR spectrum (101 MHz, $CDCl_3$) of *syn,syn*-2,4,6-trimethylnonanal (**21**) S59

^{1}H-NMR spectrum (400 MHz, $CDCl_3$) and S60

^{13}C-PENDANT-NMR spectrum (101 MHz, $CDCl_3$) of (2*E*,4*E*/*Z*)-*syn*,*syn*-4,6,8,10-tetramethyltrideca-2,4-diene (**22**) S61

^{1}H-NMR spectrum (400 MHz, $CDCl_3$) and S62

^{13}C-PENDANT-NMR spectrum (101 MHz, $CDCl_3$) of methyl (2*E*,4*E*/*Z*)-*syn*,*syn*-4,6,8,10-tetramethyltrideca-2,4-dienoate S63

^{1}H-NMR spectrum (400 MHz, $CDCl_3$) and S64

^{13}C-PENDANT-NMR spectrum (126 MHz, $CDCl_3$) of (2*E*,4*E*/*Z*)-*syn*,*syn*-4,6,8,10-tetramethyltrideca-2,4-dien-1-ol (**23**) S65

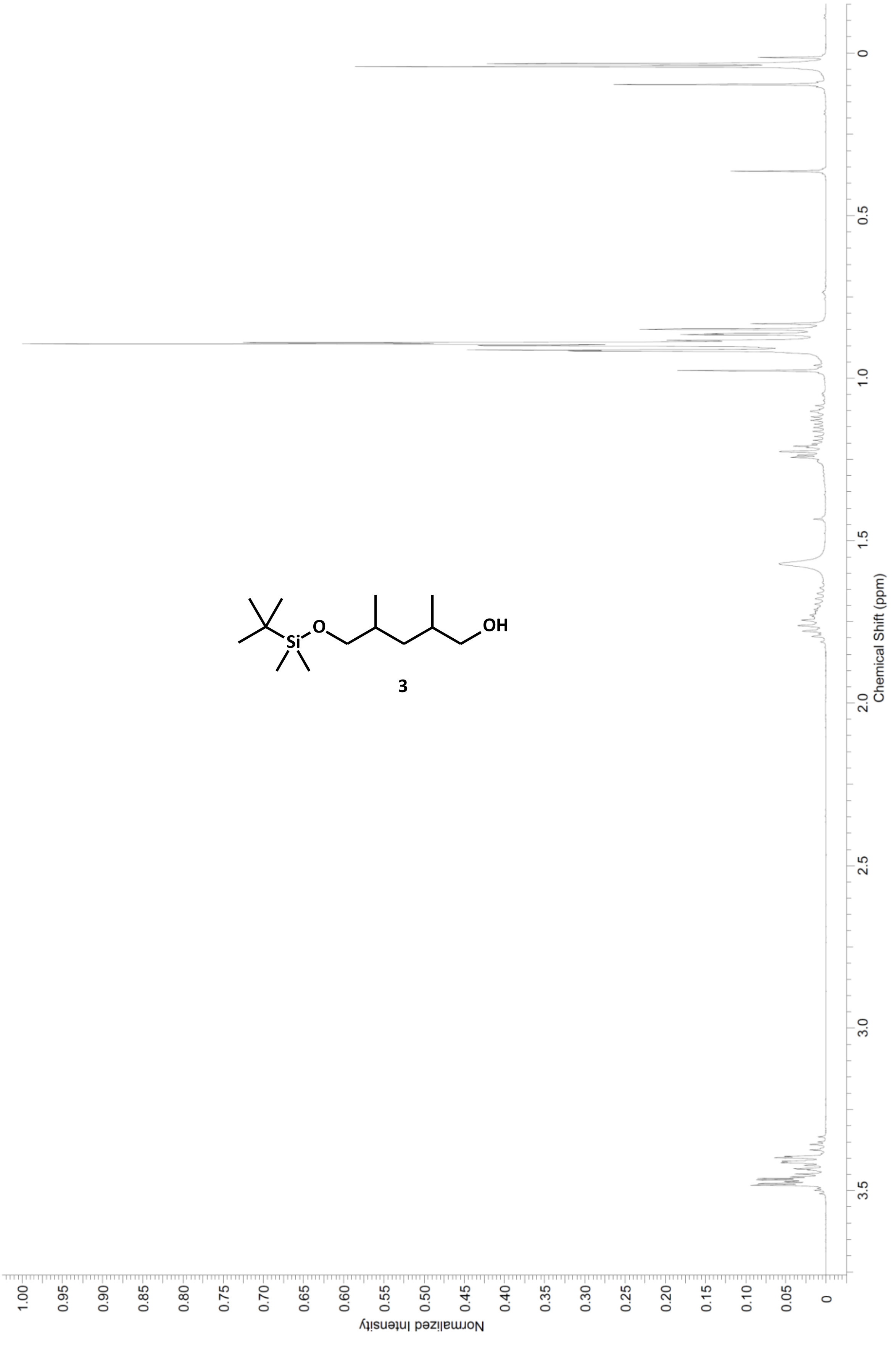

^{1}H-NMR spectrum (400 MHz, $CDCl_3$) of
anti-5-(*tert*-butyldimethylsilyloxy)-2,4-dimethylpentan-1-ol (**3**)

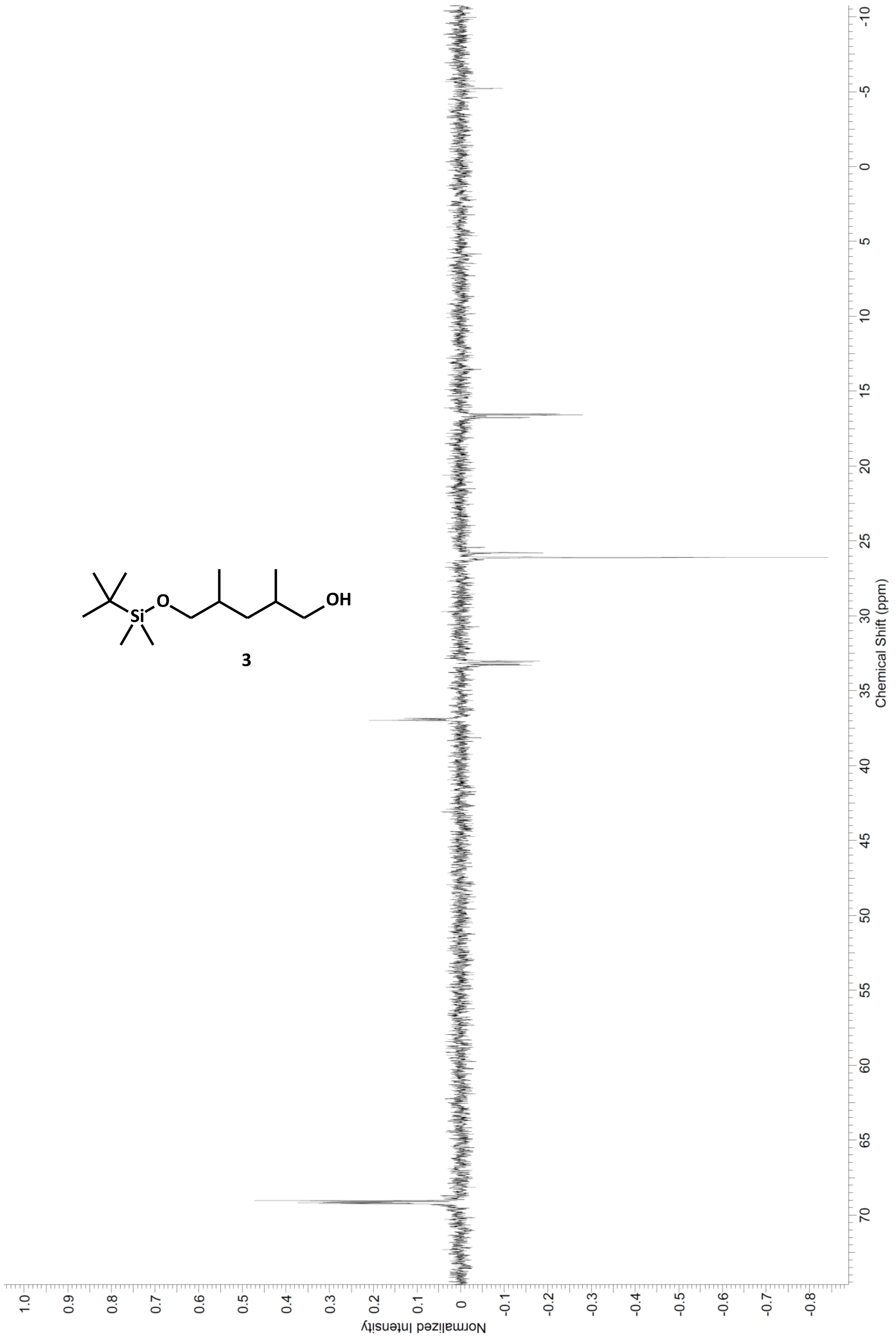

^{13}C-PENDANT-NMR spectrum (126 MHz, $CDCl_3$) of *anti*-5-(*tert*-butyldimethylsilyloxy)-2,4-dimethylpentan-1-ol (**3**)

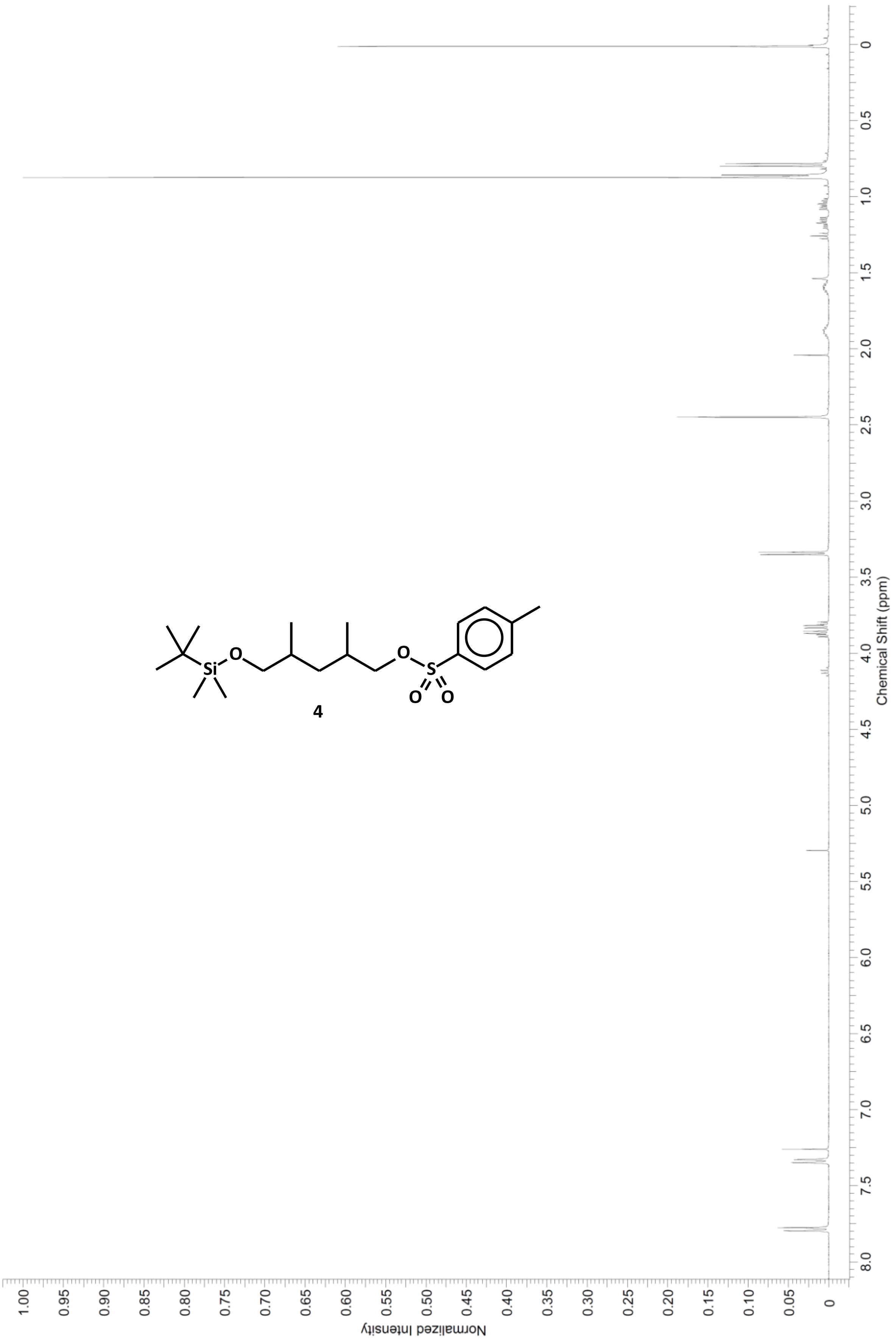

^{1}H-NMR spectrum (400 MHz, $CDCl_3$) of
anti-5-(*tert*-butyldimethylsilyloxy)-1-(*p*-toluenesulfonyloxy)-2,4-dimethylpentane (**4**)

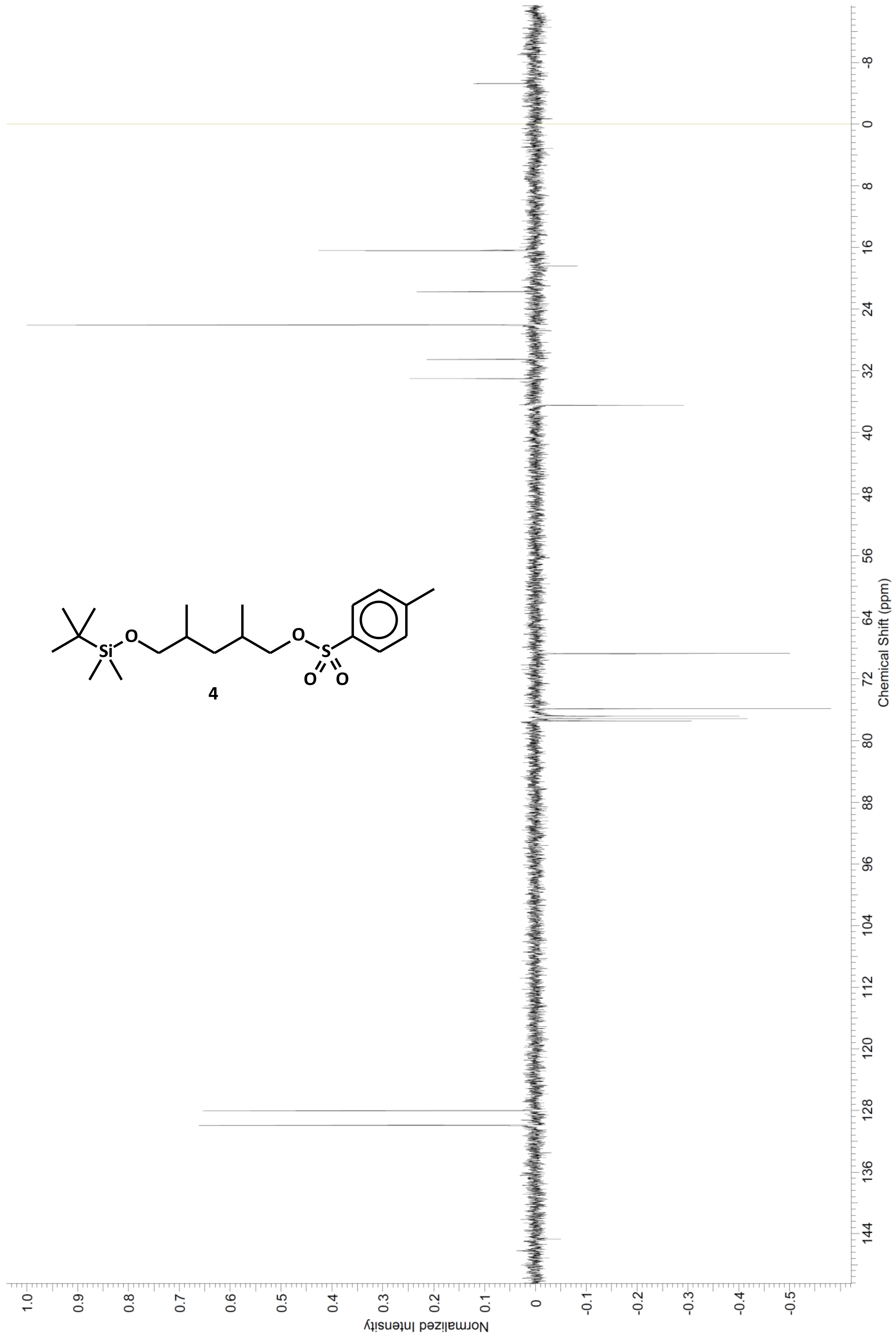

^{13}C-PENDANT-NMR spectrum (101 MHz, $CDCl_3$) of
anti-5-(*tert*-butyldimethylsilyloxy)-1-(*p*-toluenesulfonyloxy)-2,4-dimethylpentane (**4**)

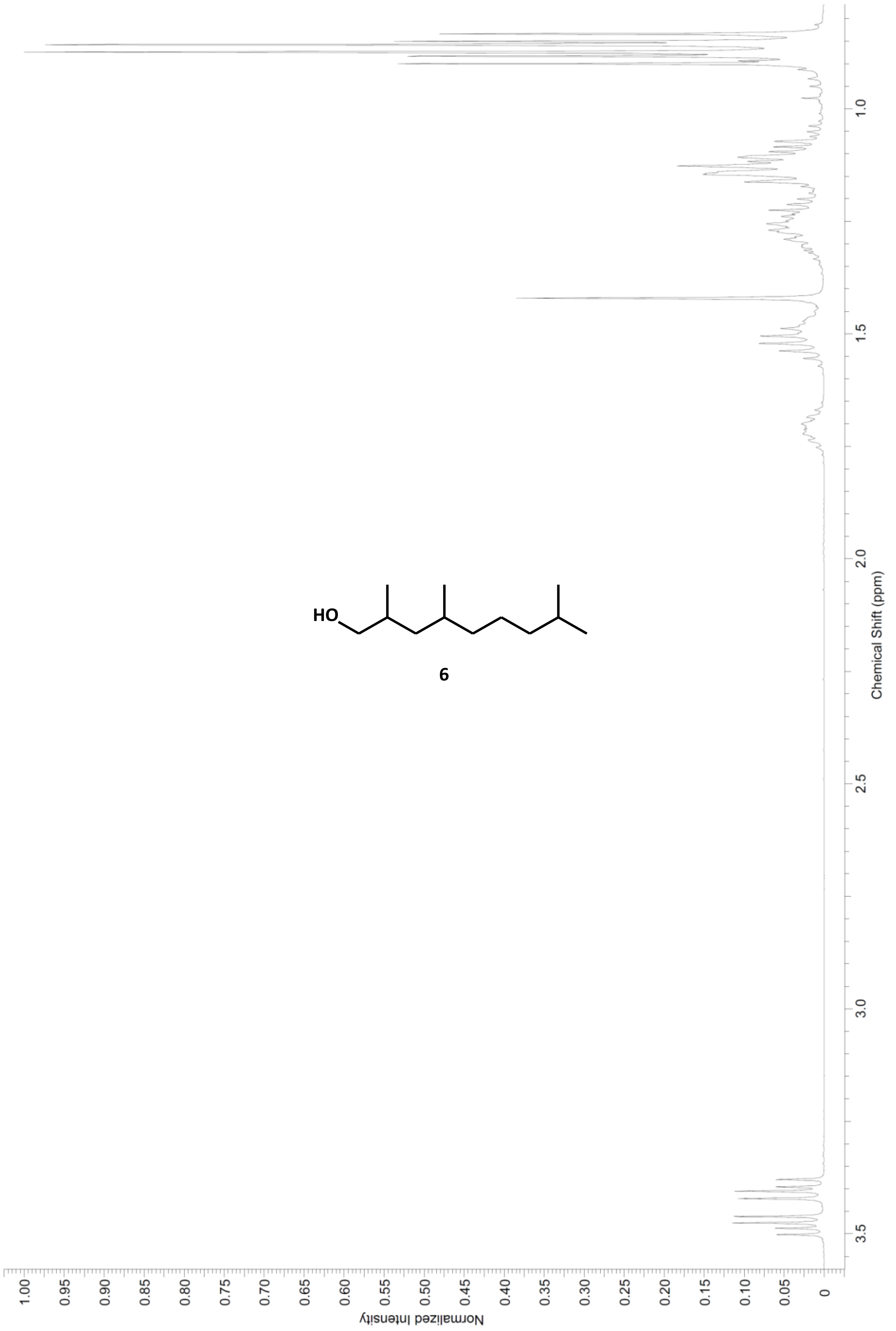

^{1}H-NMR spectrum (400 MHz, $CDCl_3$) of 2,4,8-trimethylnonan-1-ol (**6**)

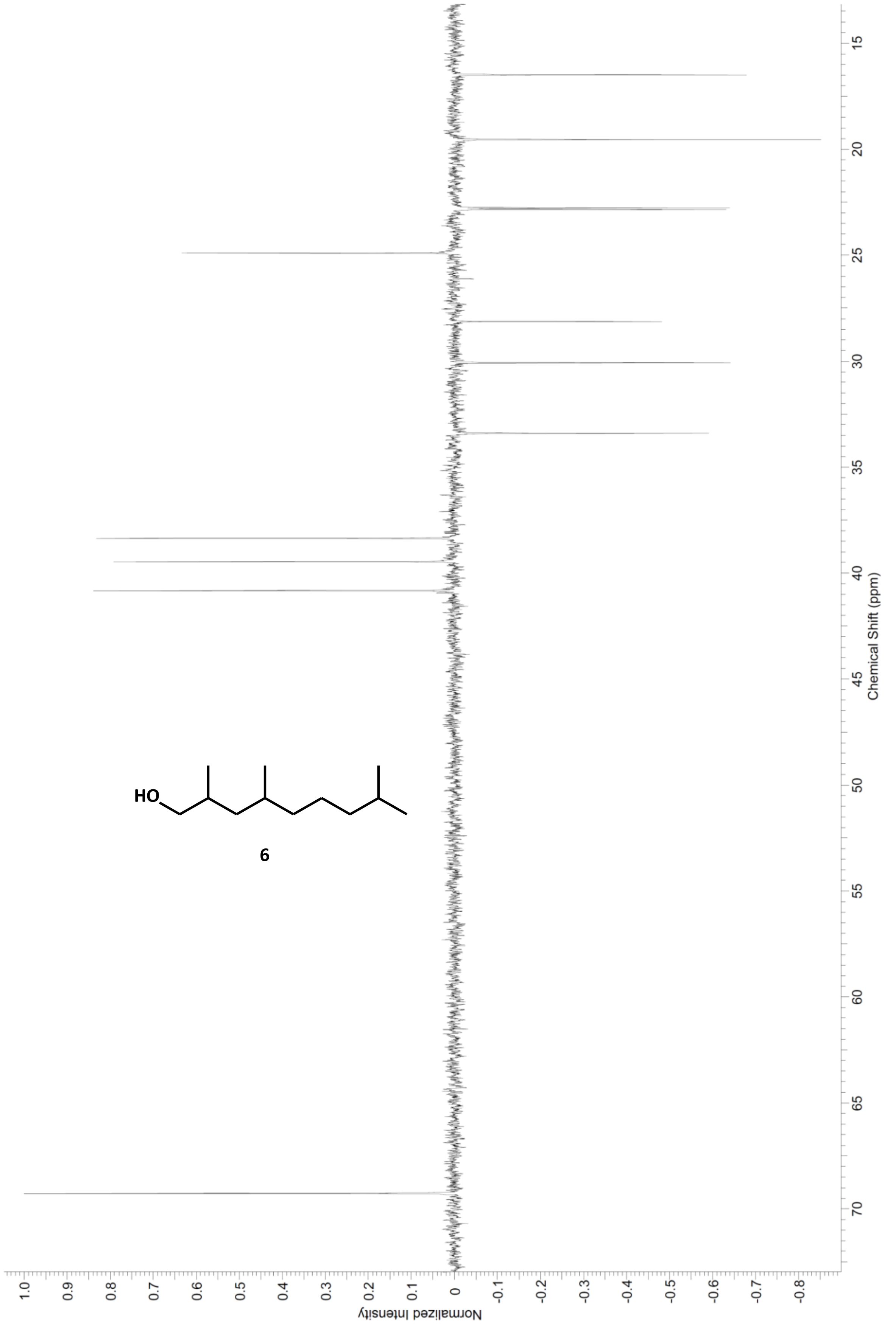

^{13}C-PENDANT-NMR spectrum (101 MHz, $CDCl_3$) of 2,4,8-trimethylnonan-1-ol (**6**)

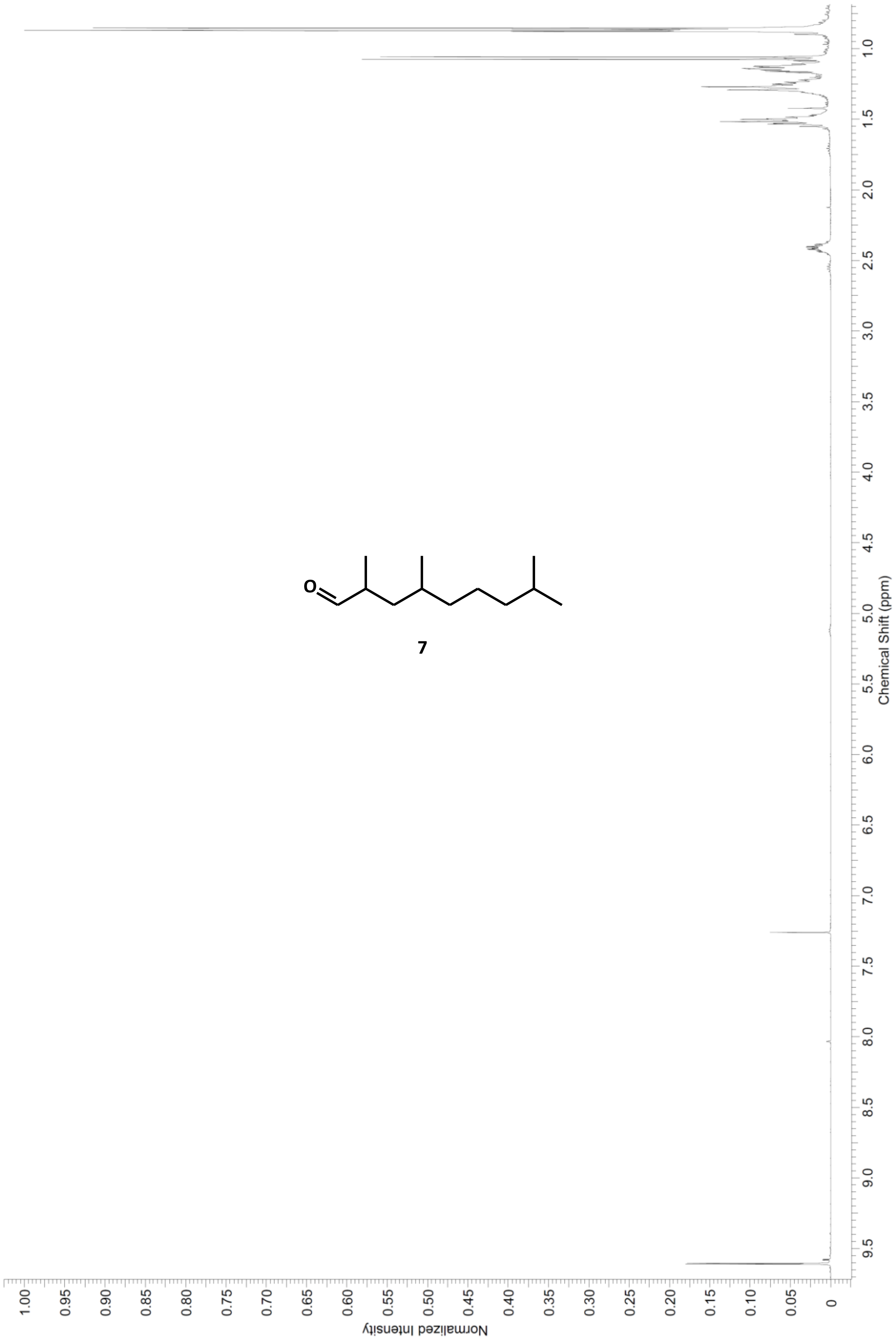

^{1}H-NMR spectrum (400 MHz, $CDCl_3$) of
2,4,8-trimethylnonanal (**7**)

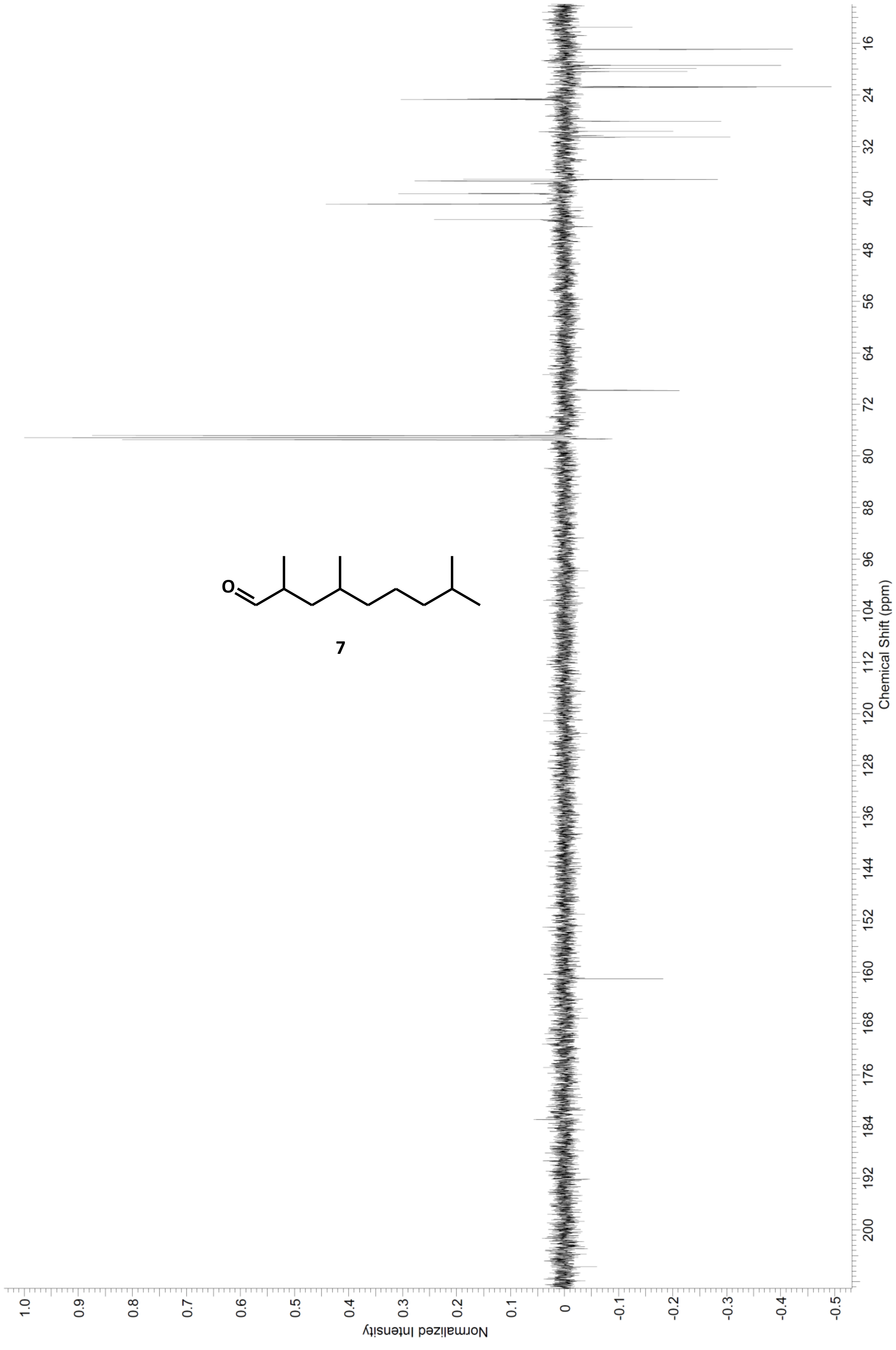

^{13}C-PENDANT-NMR spectrum (101 MHz, $CDCl_3$) of 2,4,8-trimethylnonanal (**7**)

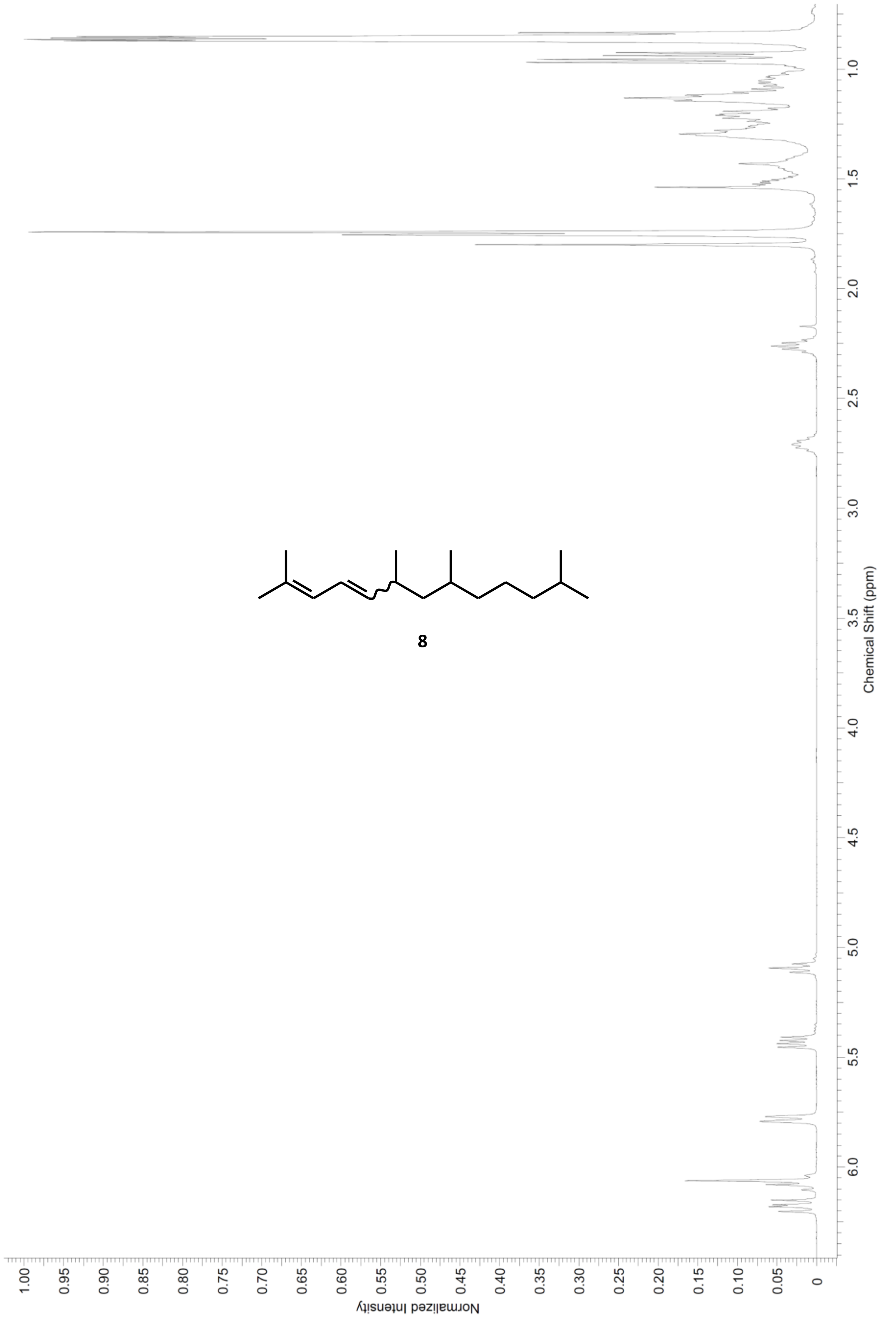

^{1}H-NMR spectrum (500 MHz, $CDCl_3$) of 2,6,8,12-tetramethyltrideca-2,4-diene (**8**)

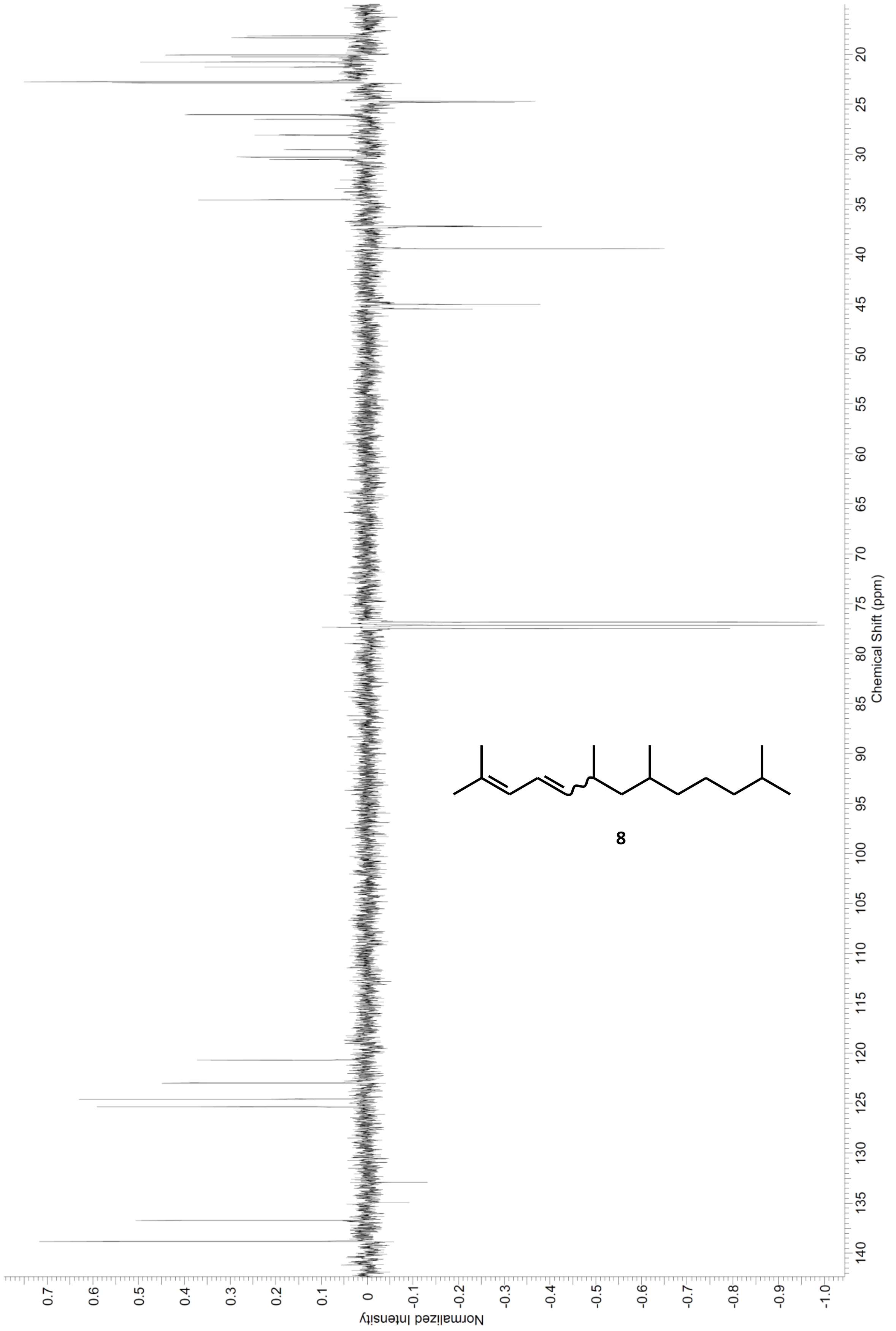

^{13}C-PENDANT-NMR spectrum (101 MHz, $CDCl_3$) of
2,6,8,12-tetramethyltrideca-2,4-diene (**8**)

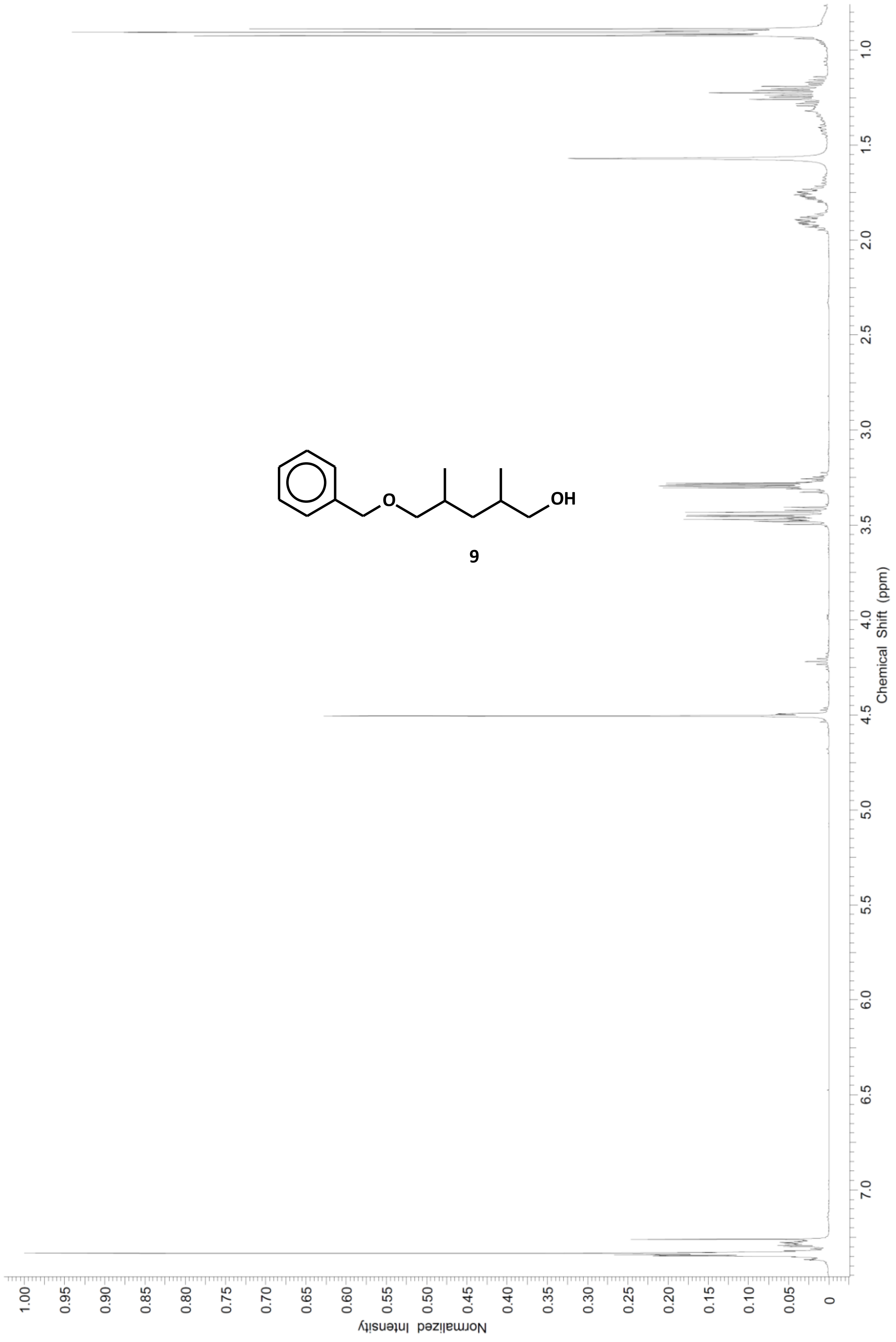

^{1}H-NMR spectrum (400 MHz, $CDCl_3$) of
anti-5-benzyloxy-2,4-dimethylpentan-1-ol (**9**)

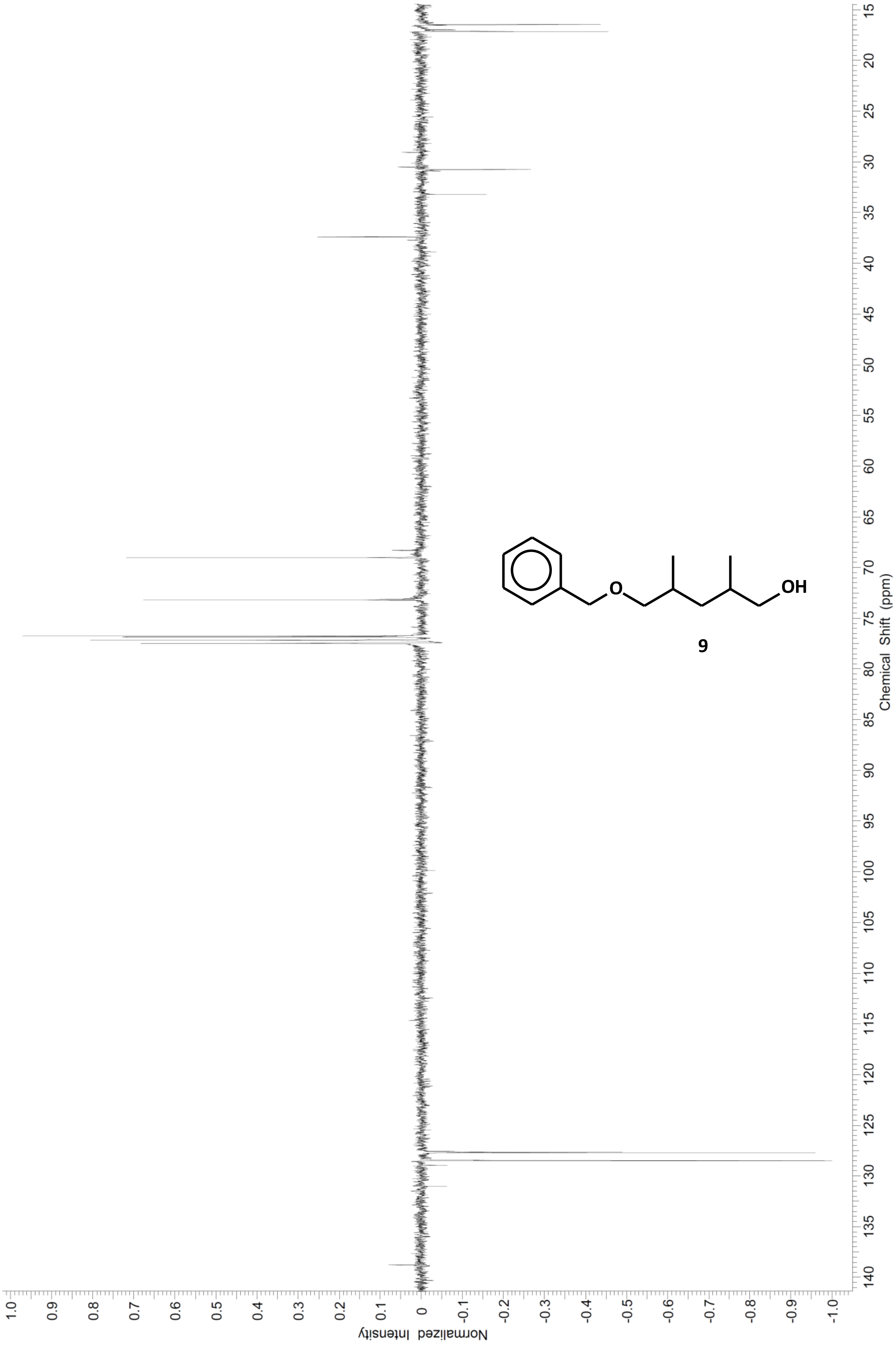

^{13}C-PENDANT-NMR spectrum (101 MHz, $CDCl_3$) of *anti*-5-benzyloxy-2,4-dimethylpentan-1-ol (**9**)

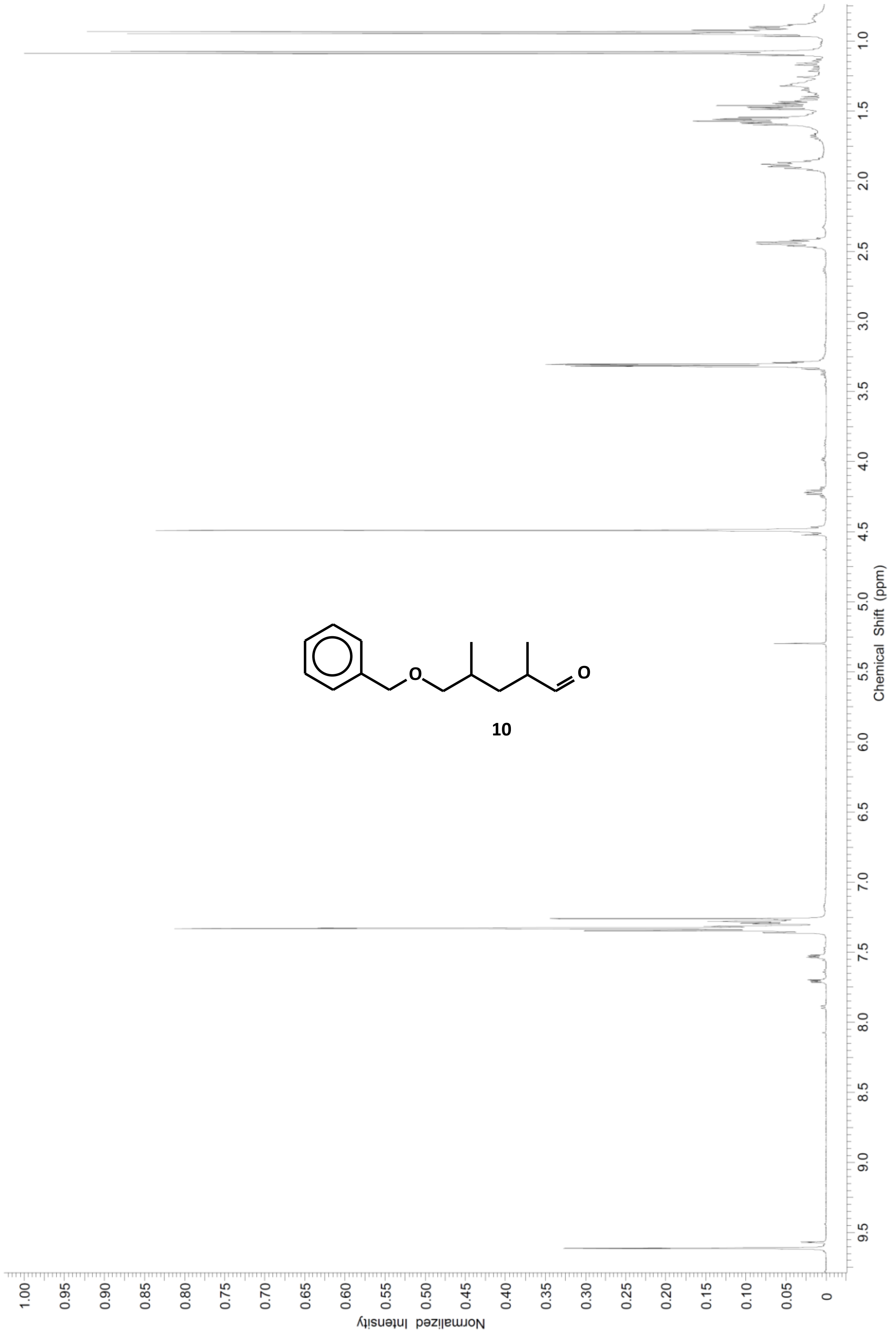

^{1}H-NMR spectrum (500 MHz, $CDCl_3$) of
anti-5-benzyloxy-2,4-dimethylpentanal (**10**)

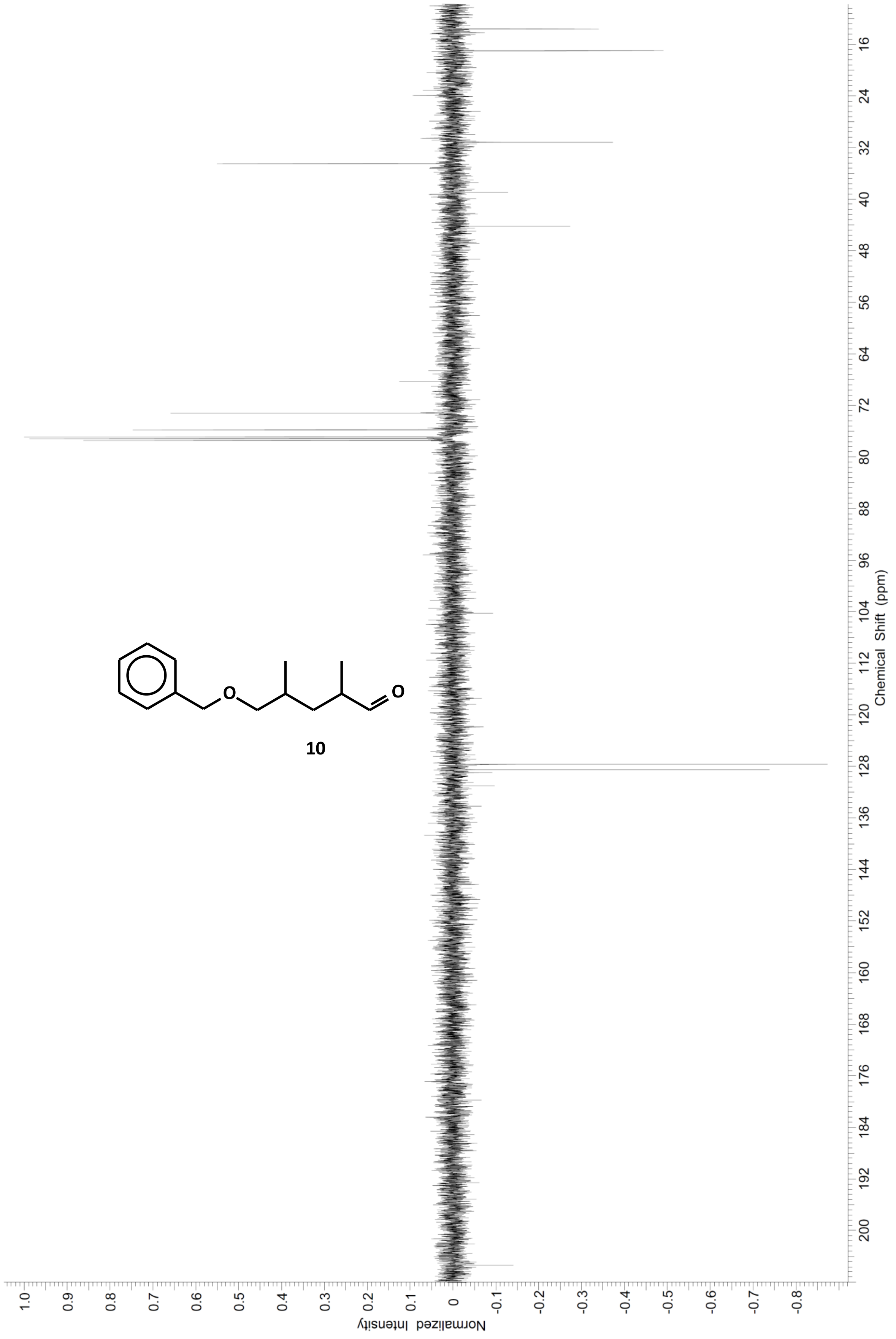

^{13}C-PENDANT-NMR spectrum (126 MHz, $CDCl_3$) of
anti-5-benzyloxy-2,4-dimethylpentanal (**10**)

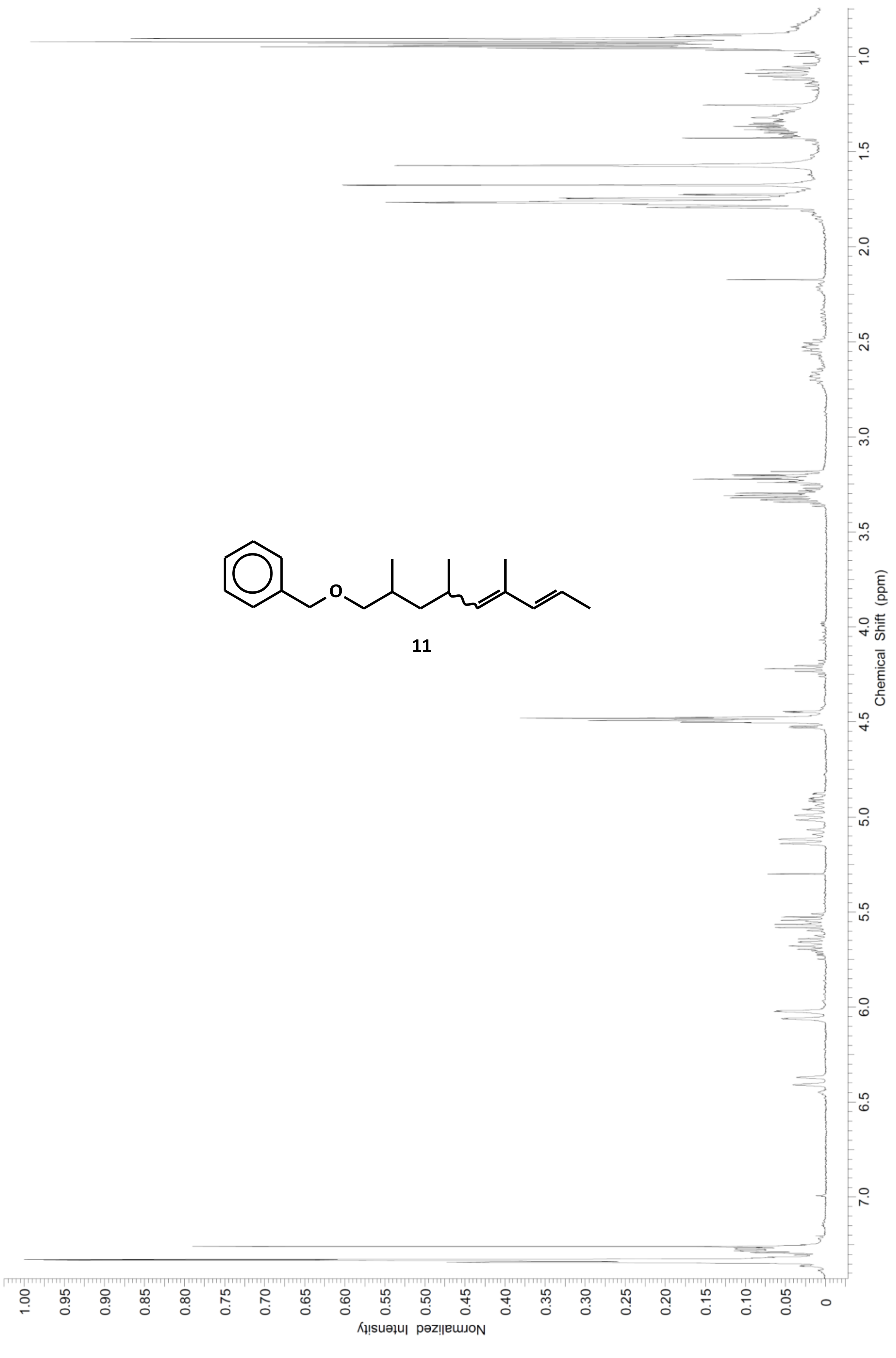

^{1}H-NMR spectrum (400 MHz, $CDCl_3$) of
(5*E*/*Z*,7*E*)-*anti*-1-benzyloxy-2,4,6-trimethylnona-5,7-diene (**11**)

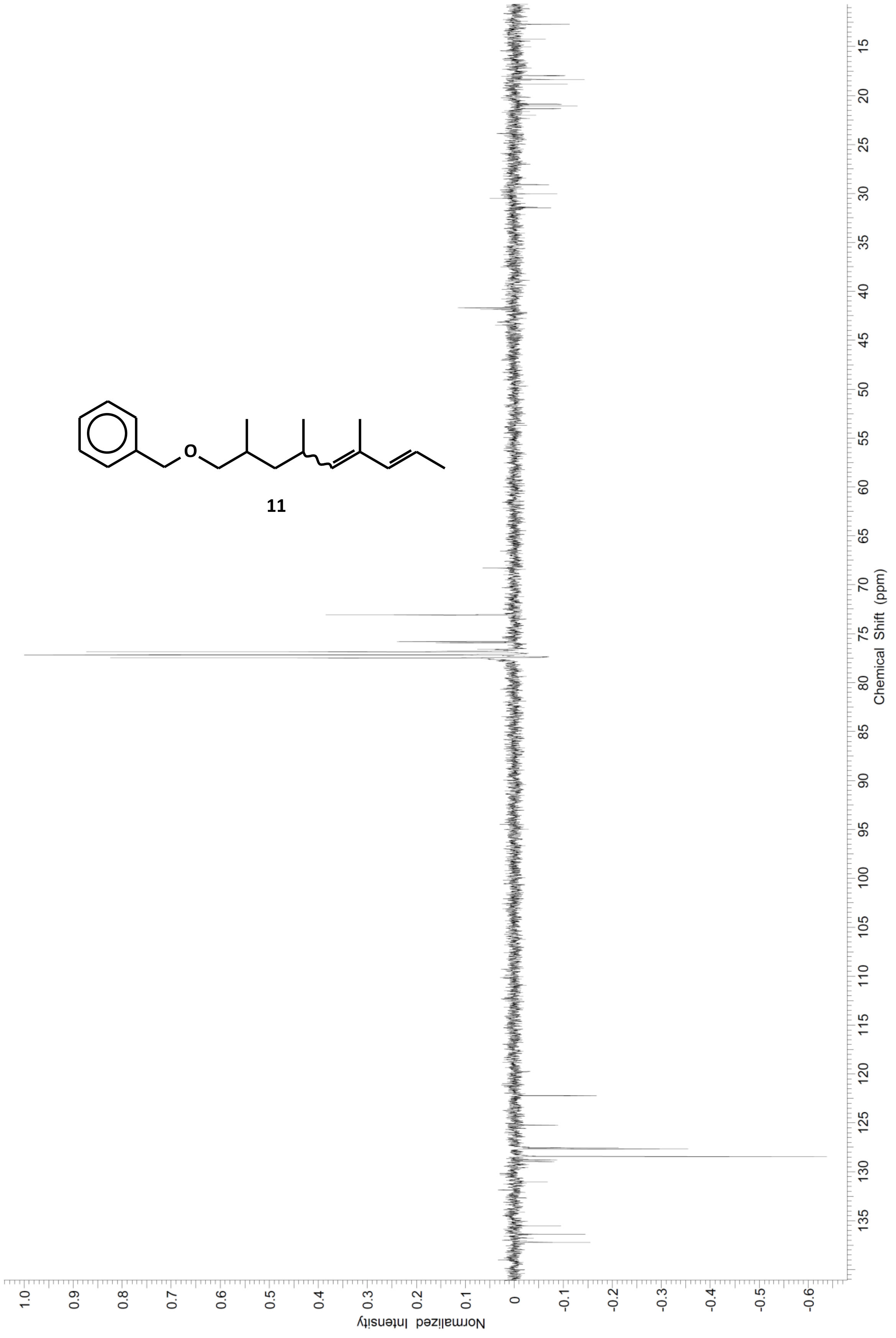

^{13}C-PENDANT-NMR spectrum (101 MHz, $CDCl_3$) of (5*E*/*Z*,7*E*)-*anti*-1-benzyloxy-2,4,6-trimethylnona-5,7-diene (**11**)

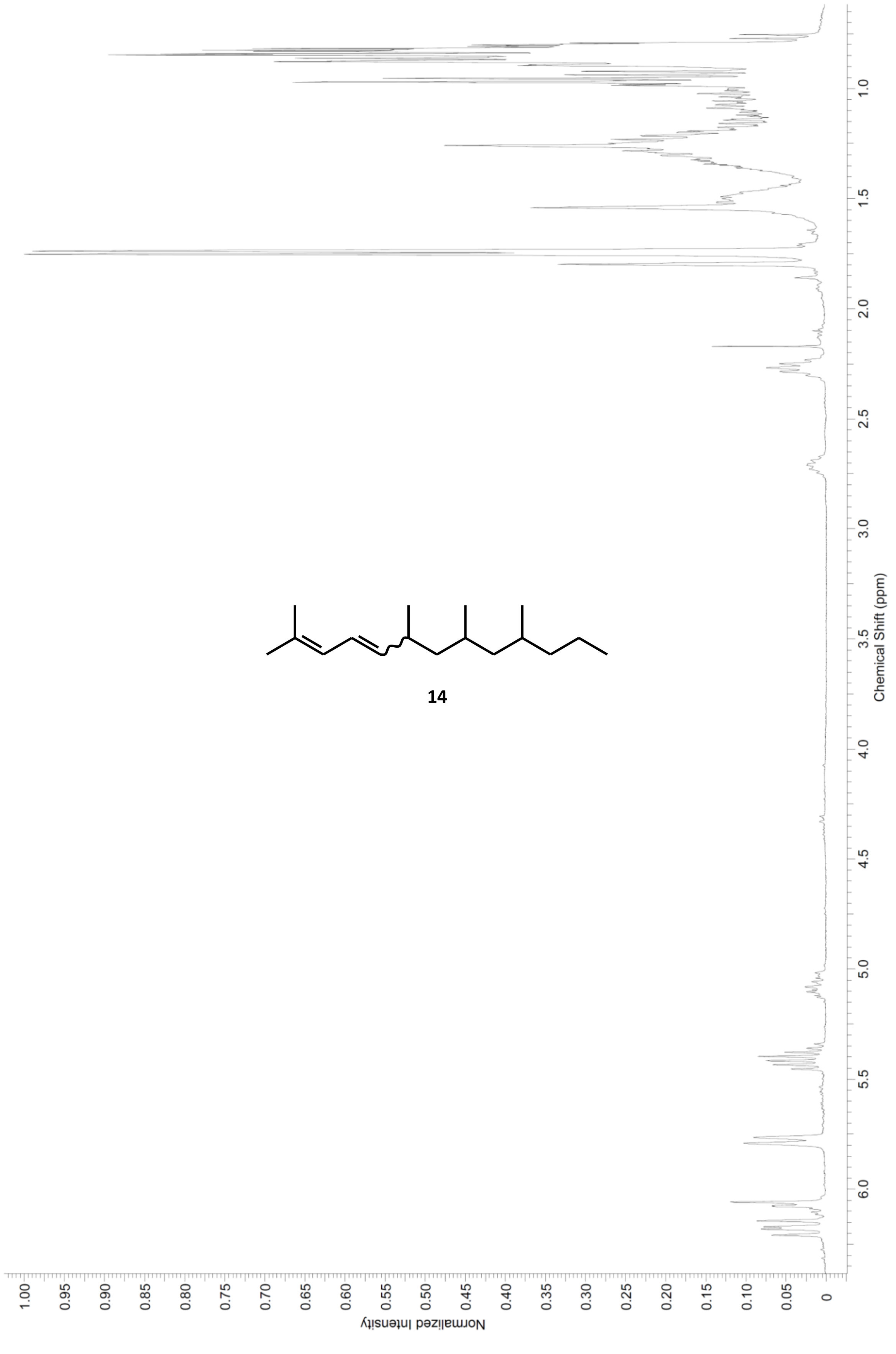

^{1}H-NMR spectrum (400 MHz, $CDCl_3$) of
(4*E*/*Z*)-2,6,8,10-tetramethyltrideca-2,4-diene (**14**)

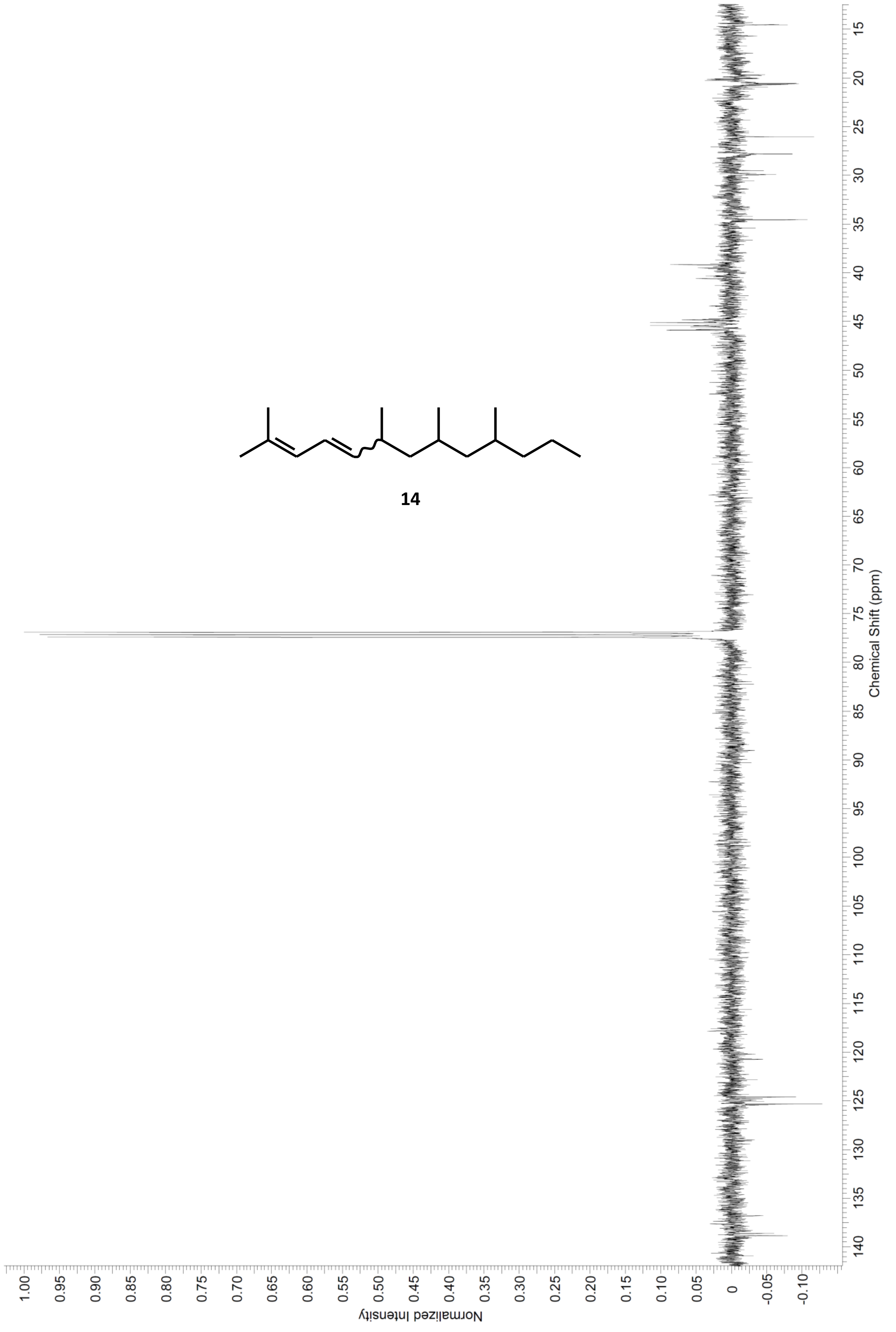

^{13}C-PENDANT-NMR spectrum (126 MHz, $CDCl_3$) of (4*E*/*Z*)-2,6,8,10-tetramethyltrideca-2,4-diene (**14**)

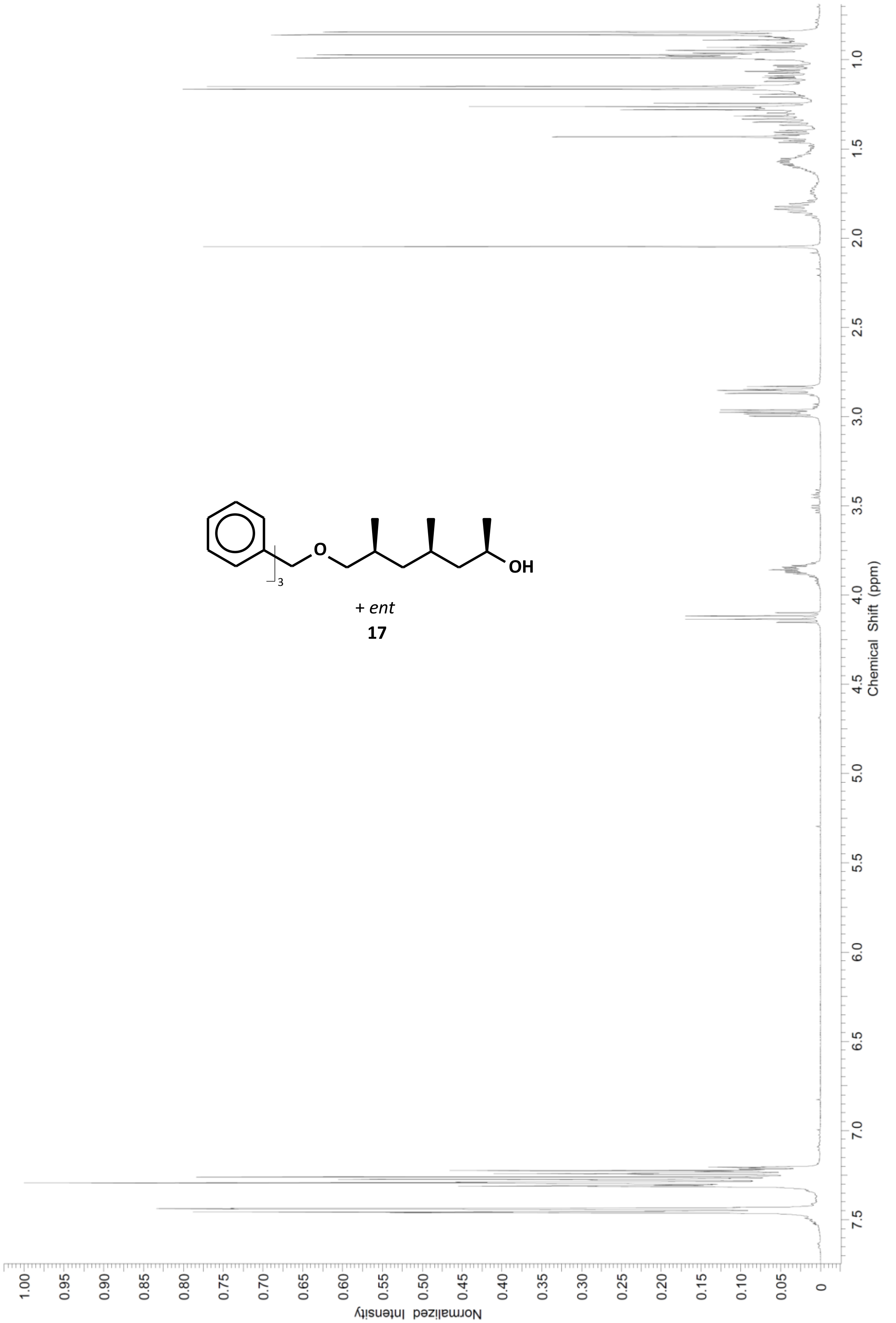

^{1}H-NMR spectrum (400 MHz, $CDCl_3$) of
syn,*syn*-4,6-dimethyl-7-(triphenylmethyloxy)heptan-2-ol (**17**)

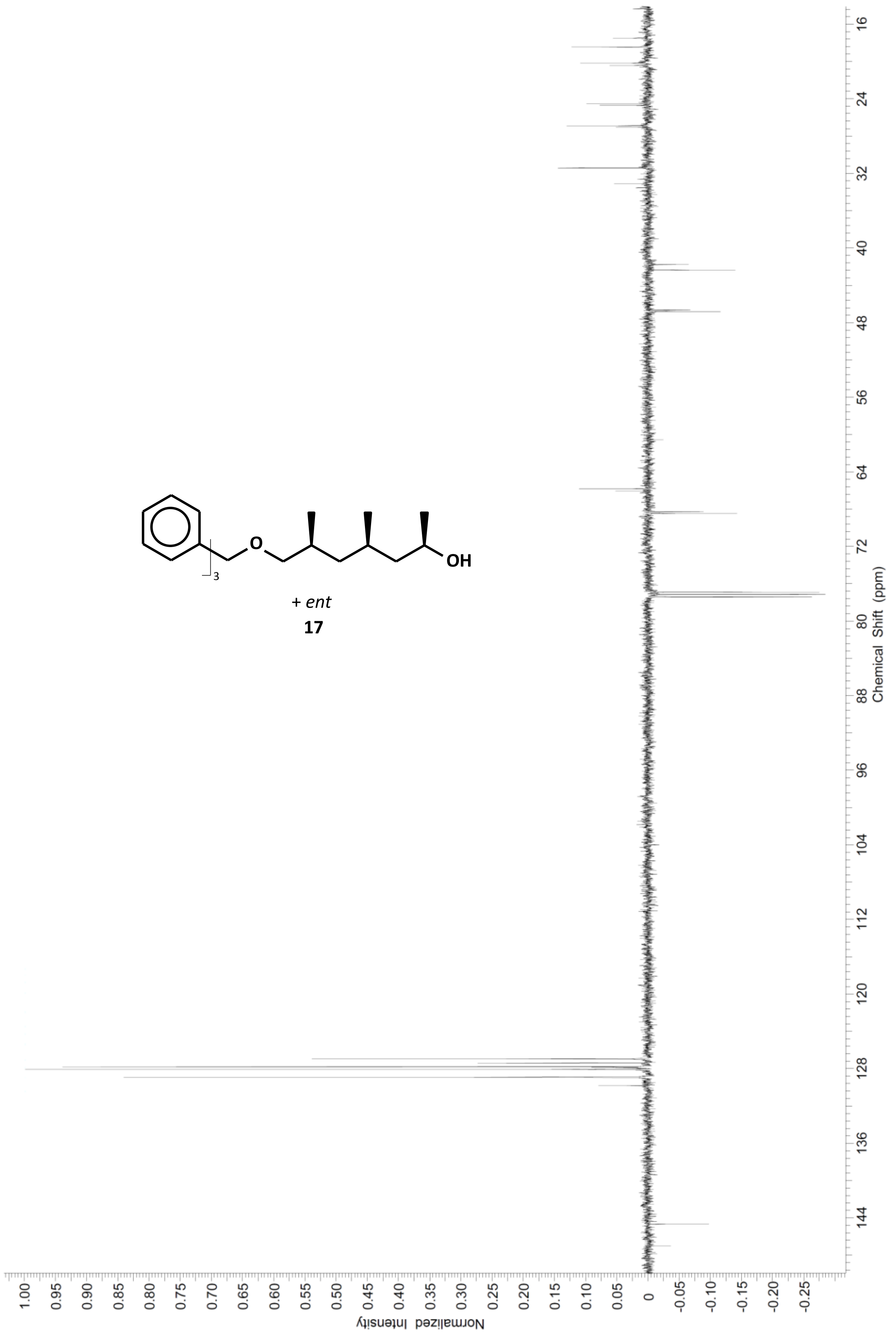

^{13}C-PENDANT-NMR spectrum (126 MHz, $CDCl_3$) of *syn*,*syn*-4,6-dimethyl-7-(triphenylmethyloxy)heptan-2-ol (**17**)

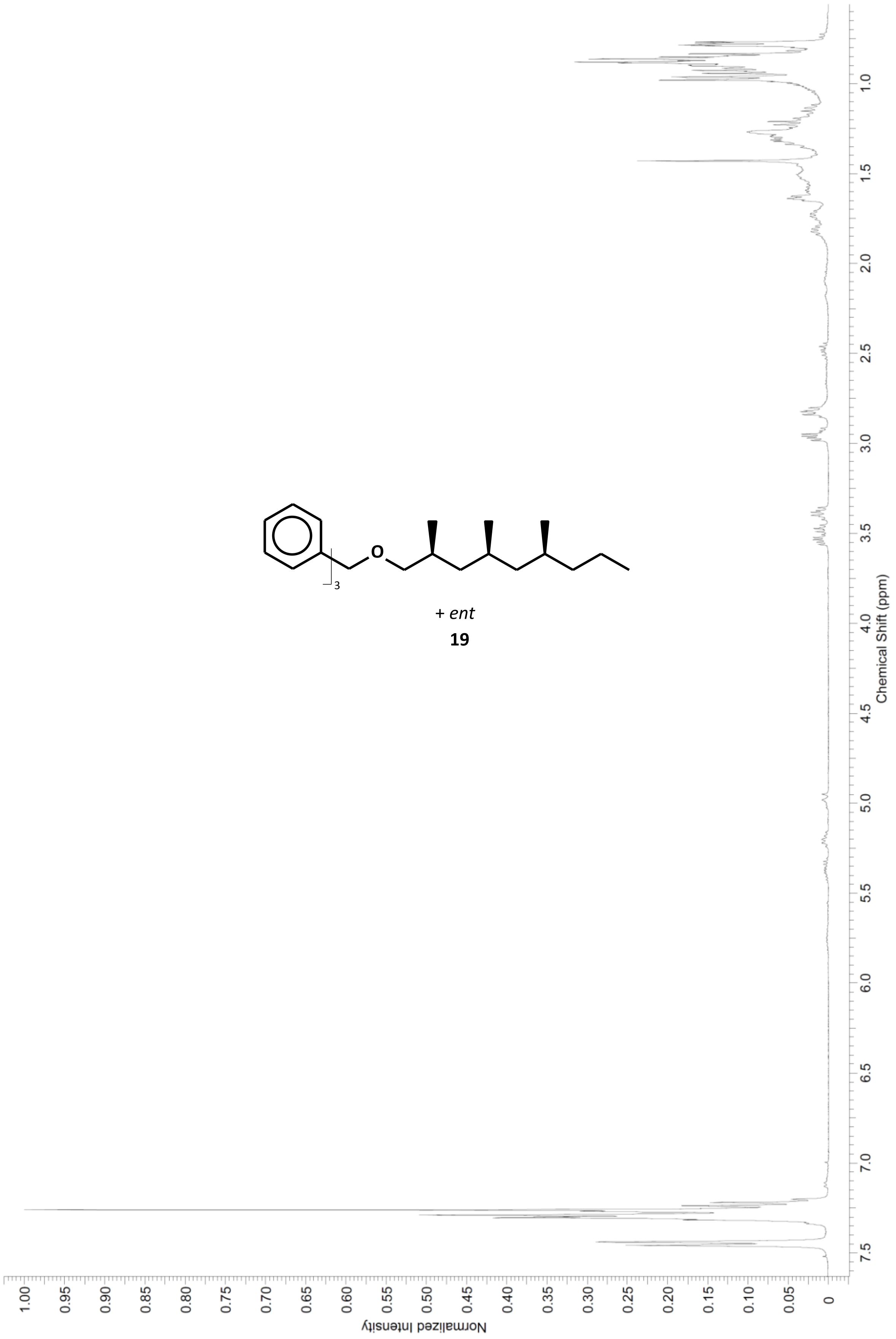

^{1}H-NMR spectrum (400 MHz, $CDCl_3$) of

syn-*syn*-2,4,6-trimethyl-1-(triphenylmethyloxy)nonane (**19**)

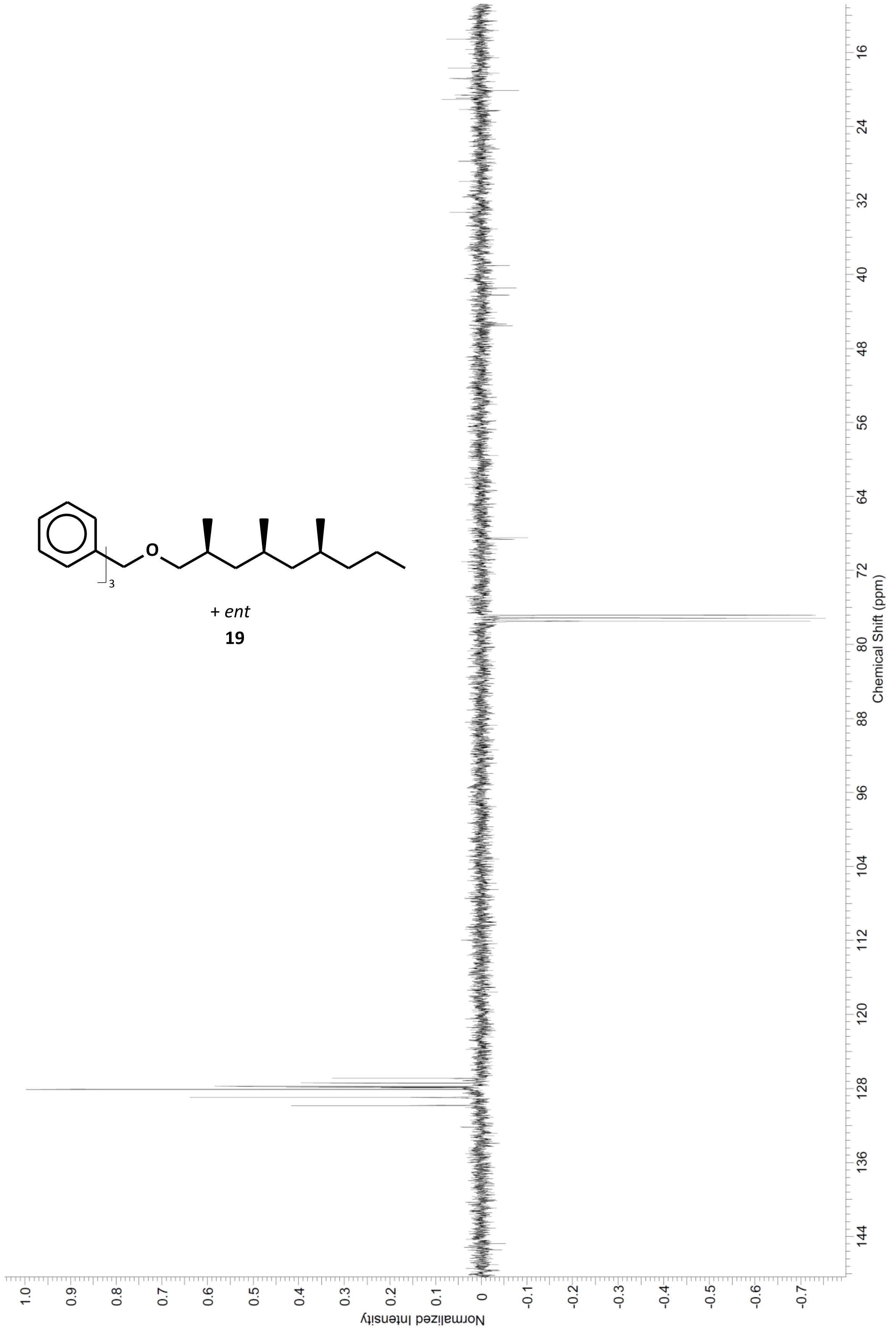

^{13}C-PENDANT-NMR spectrum (101 MHz, $CDCl_3$) of *syn*-*syn*-2,4,6-trimethyl-1-(triphenylmethyloxy)nonane (**19**)

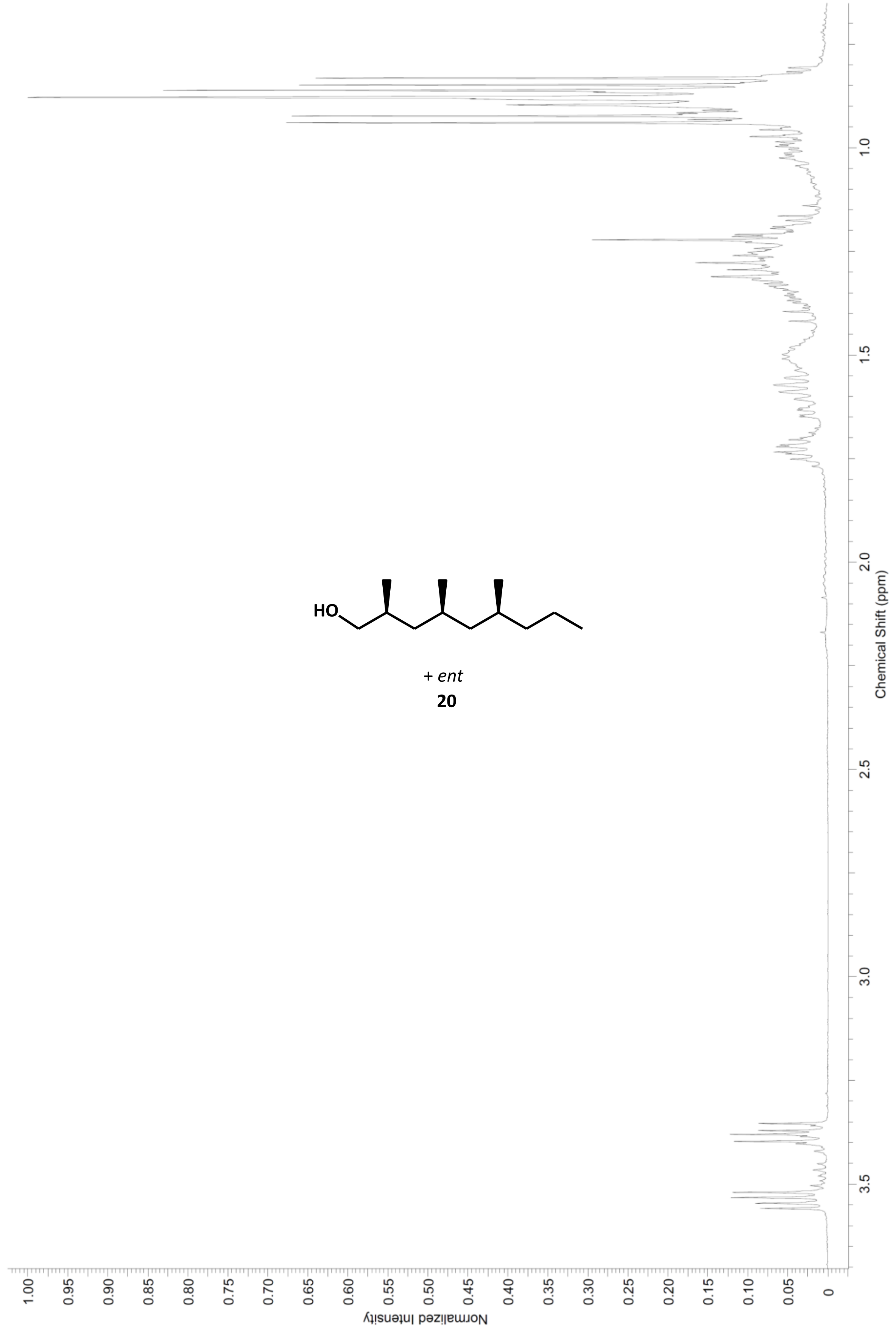

^{1}H-NMR spectrum (400 MHz, $CDCl_3$) of
syn,*syn*-2,4,6-trimethylnonan-1-ol (**20**)

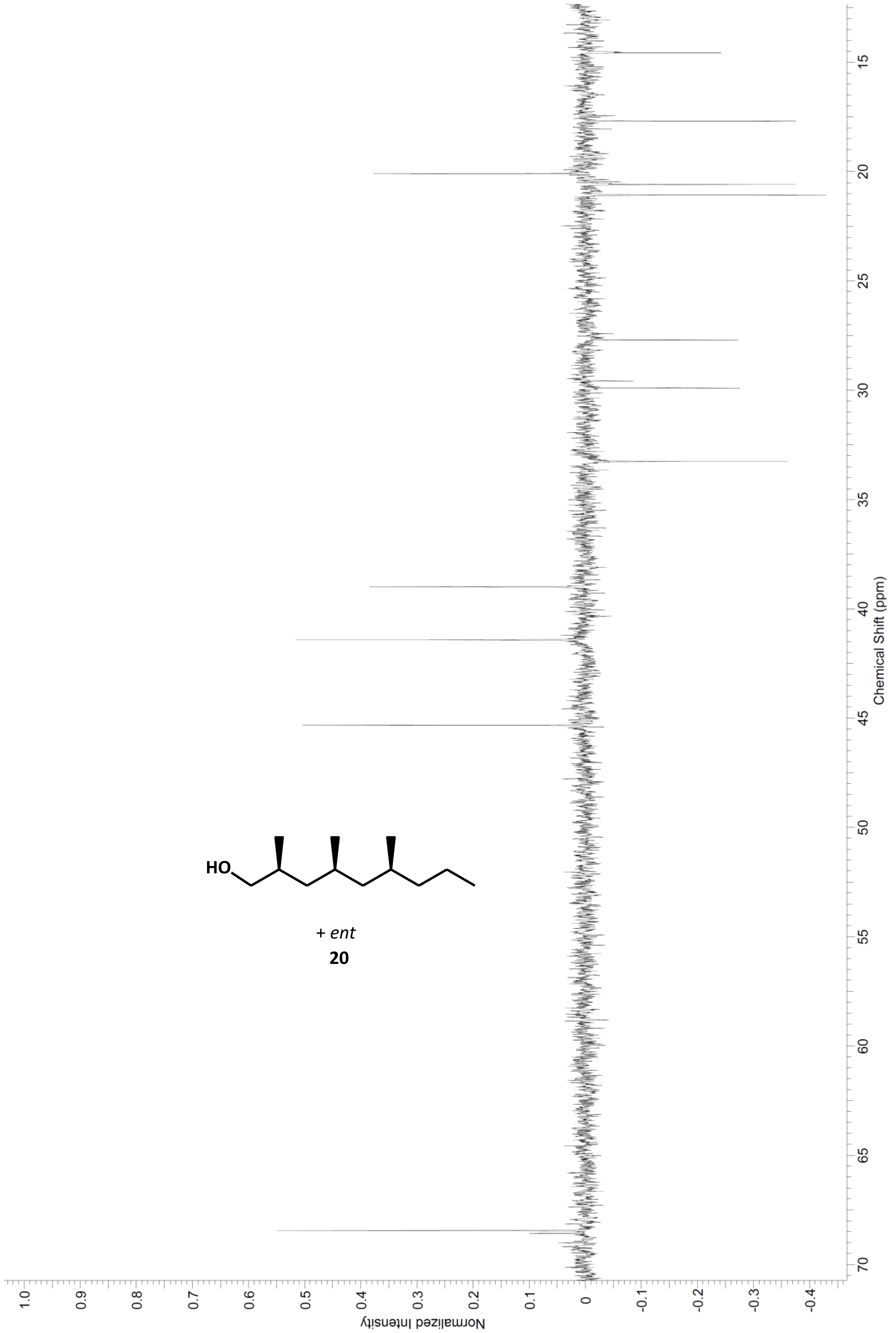

^{13}C-PENDANT-NMR spectrum (101 MHz, $CDCl_3$) of *syn*,*syn*-2,4,6-trimethylnonan-1-ol (**20**)

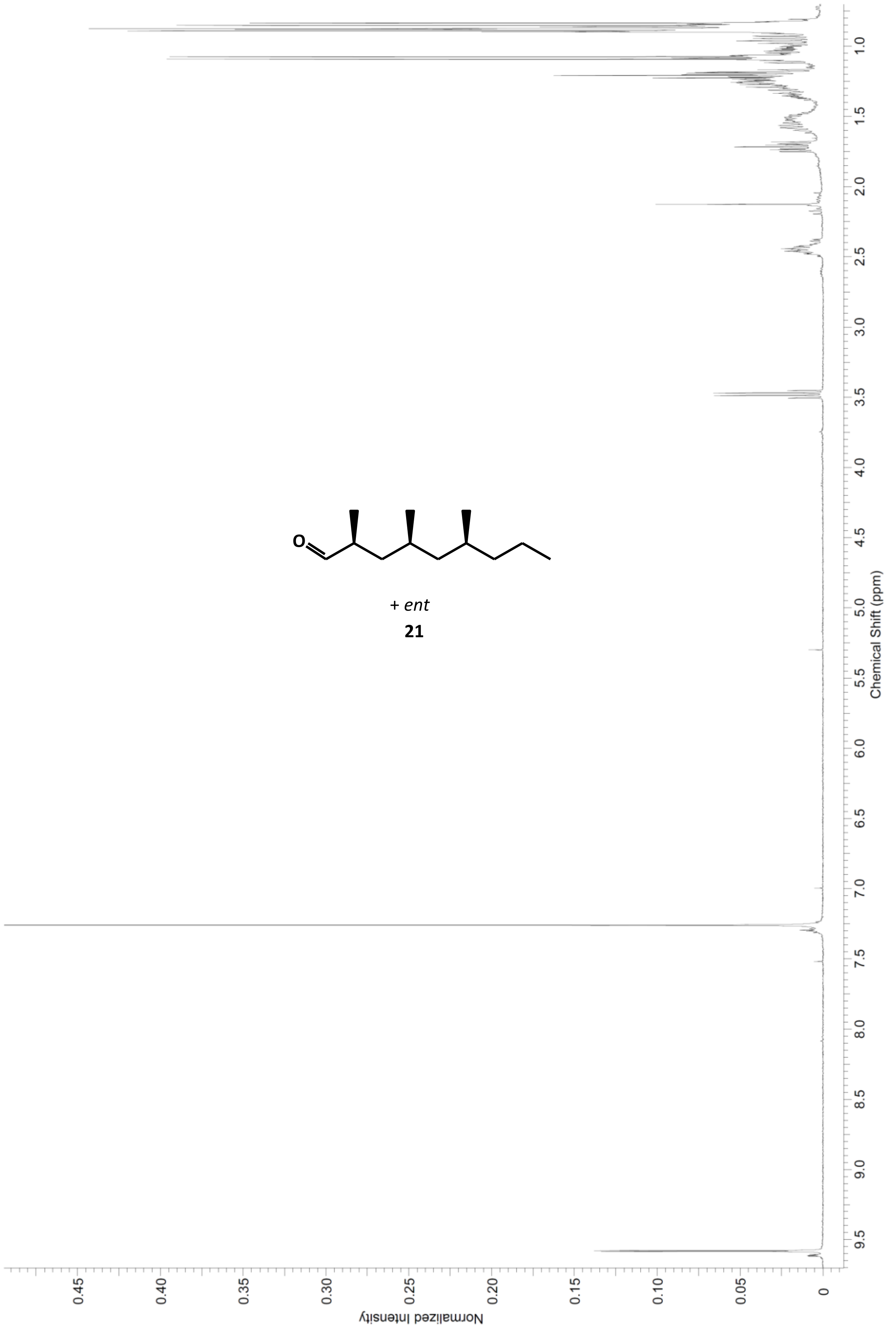

^{1}H-NMR spectrum (400 MHz, $CDCl_3$) of *syn*,*syn*-2,4,6-trimethylnonanal (**21**)

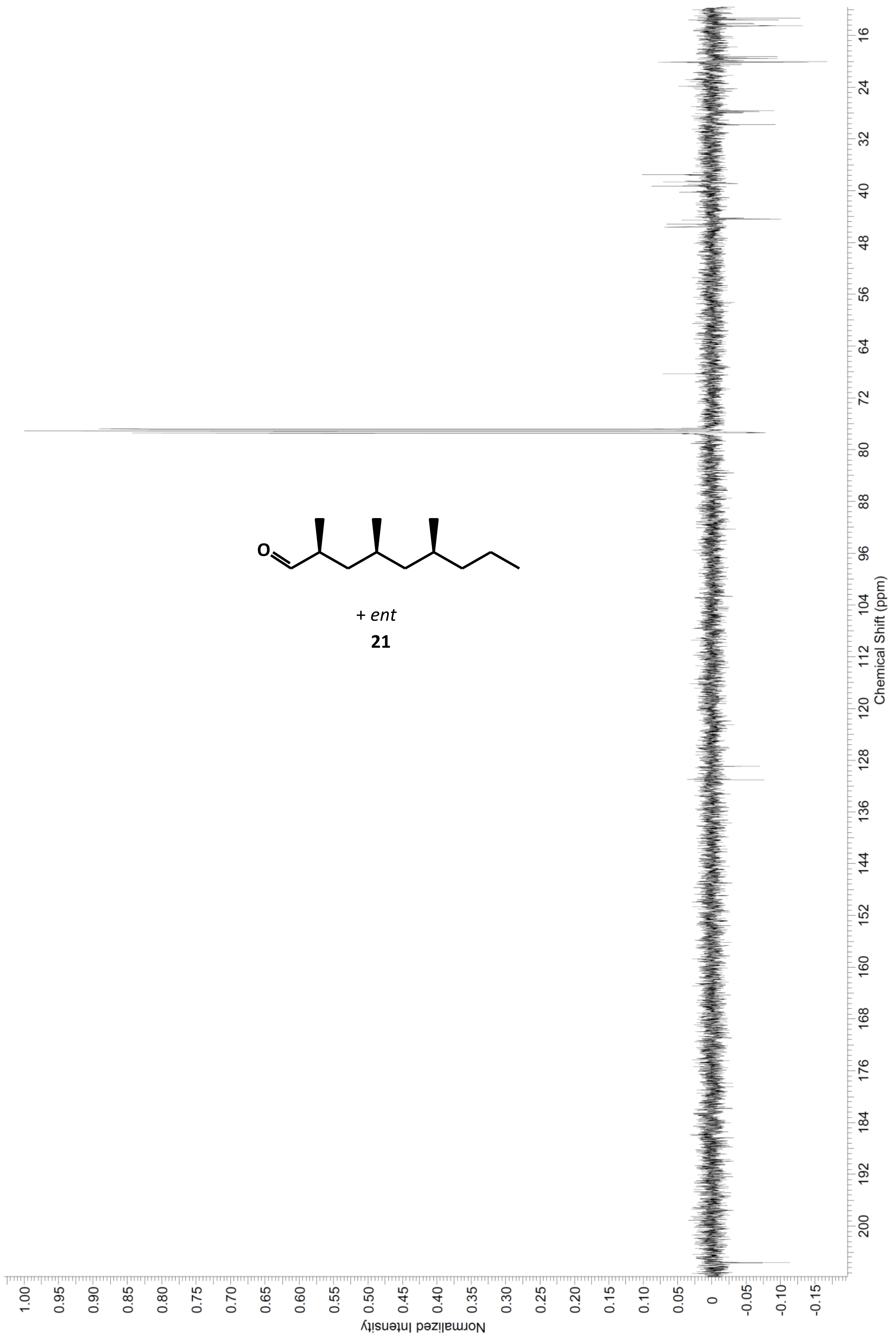

^{13}C-PENDANT-NMR spectrum (101 MHz, $CDCl_3$) of *syn*,*syn*-2,4,6-trimethylnonanal (**21**)

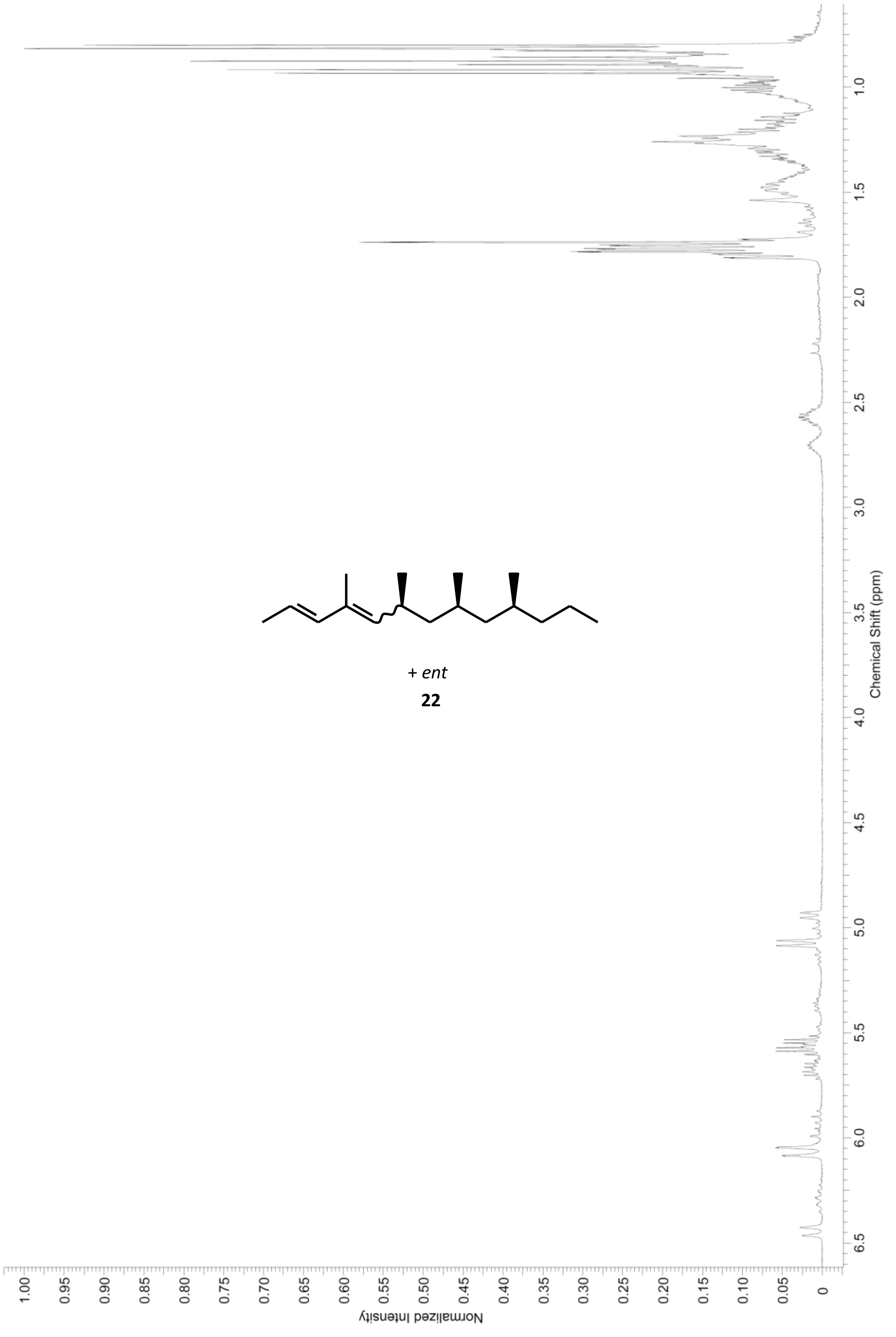

^{1}H-NMR spectrum (400 MHz, $CDCl_3$) of
(2*E*,4*E*/*Z*)-*syn*,*syn*-4,6,8,10-tetramethyltrideca-2,4-diene (**22**)

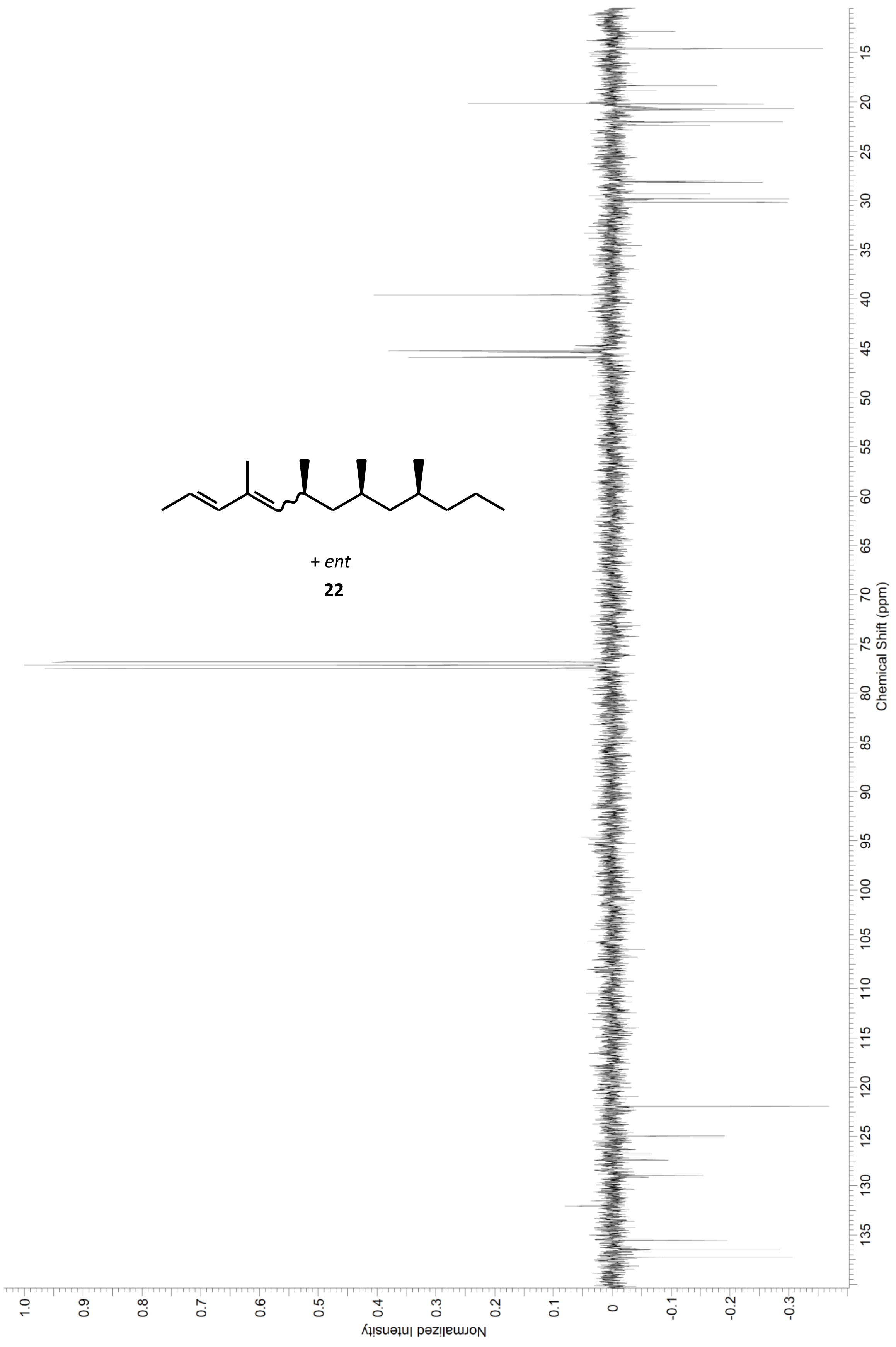

^{13}C-PENDANT-NMR spectrum (101 MHz, $CDCl_3$) of (2*E*,4*E*/*Z*)-*syn*,*syn*-4,6,8,10-tetramethyltrideca-2,4-diene (**22**)

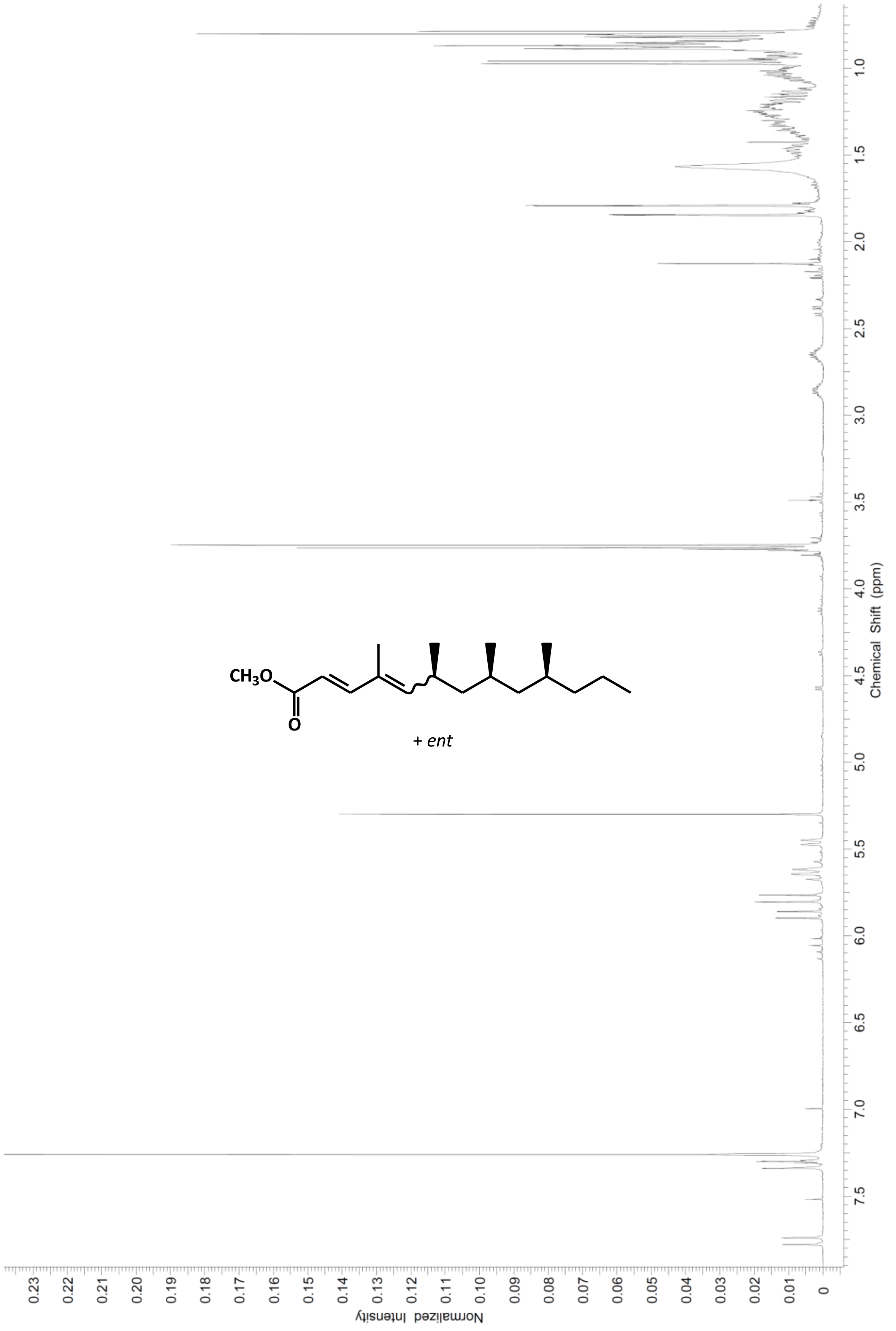

^{1}H-NMR spectrum (400 MHz, $CDCl_3$) of
methyl (2*E*,4*E*/*Z*)-*syn*,*syn*-4,6,8,10-tetramethyltrideca-2,4-dienoate

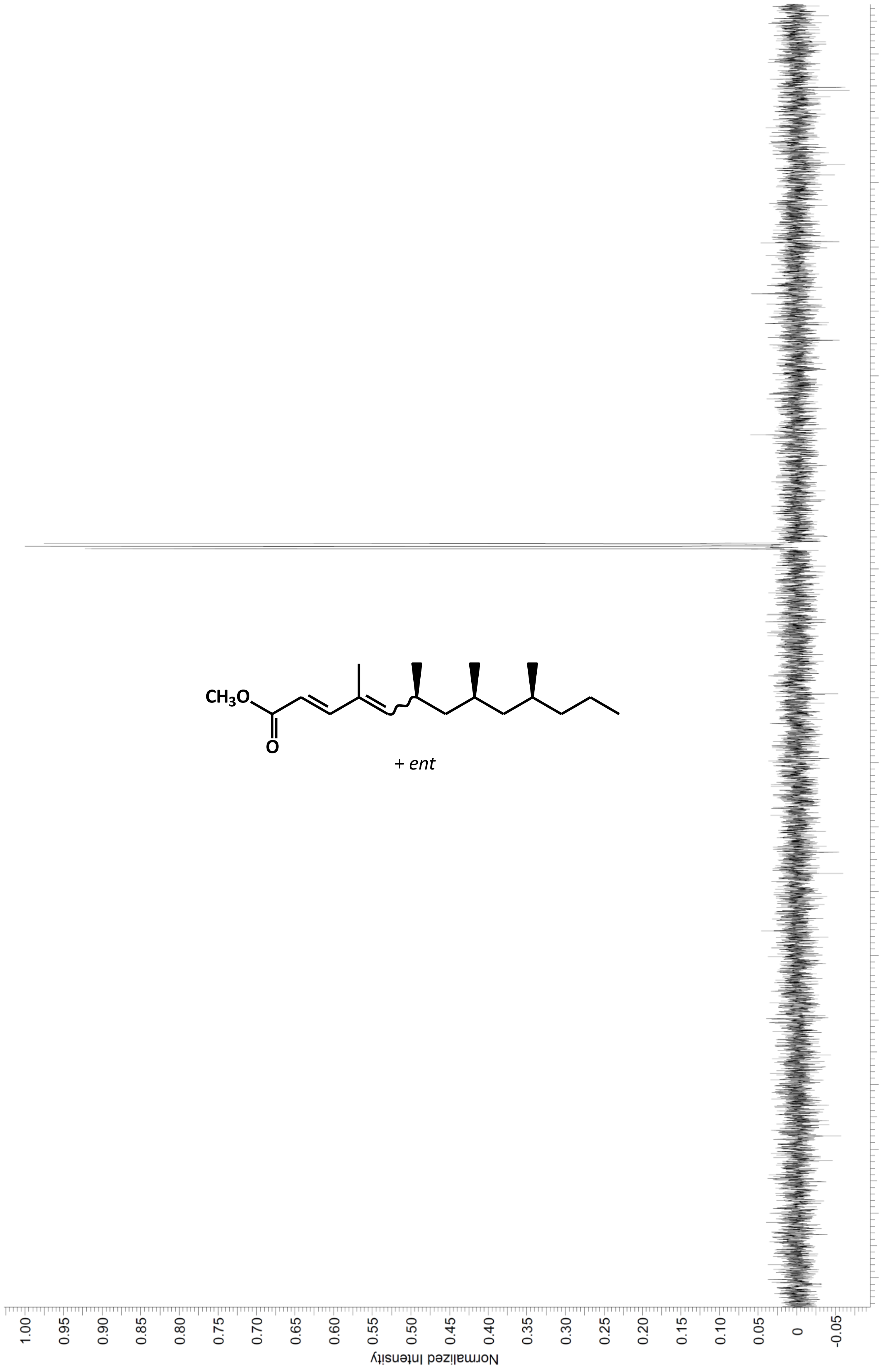

^{13}C-PENDANT-NMR spectrum (101 MHz, $CDCl_3$) of
methyl (2*E*,4*E*/*Z*)-*syn*,*syn*-4,6,8,10-tetramethyltrideca-2,4-dienoate

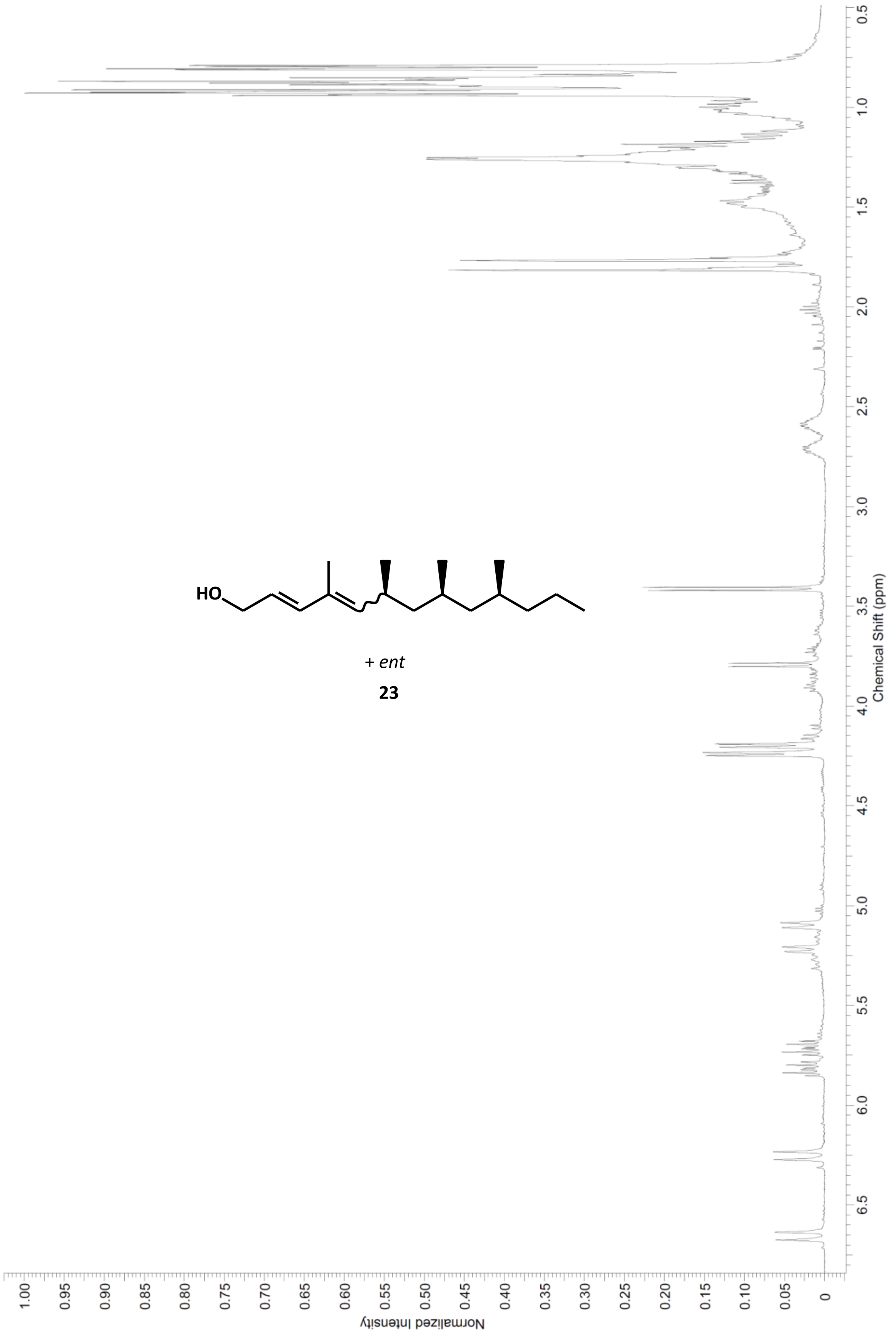

^{1}H-NMR spectrum (400 MHz, $CDCl_3$) of
(2*E*,4*E*/*Z*)-*syn*,*syn*-4,6,8,10-tetramethyltrideca-2,4-dien-1-ol (**23**)

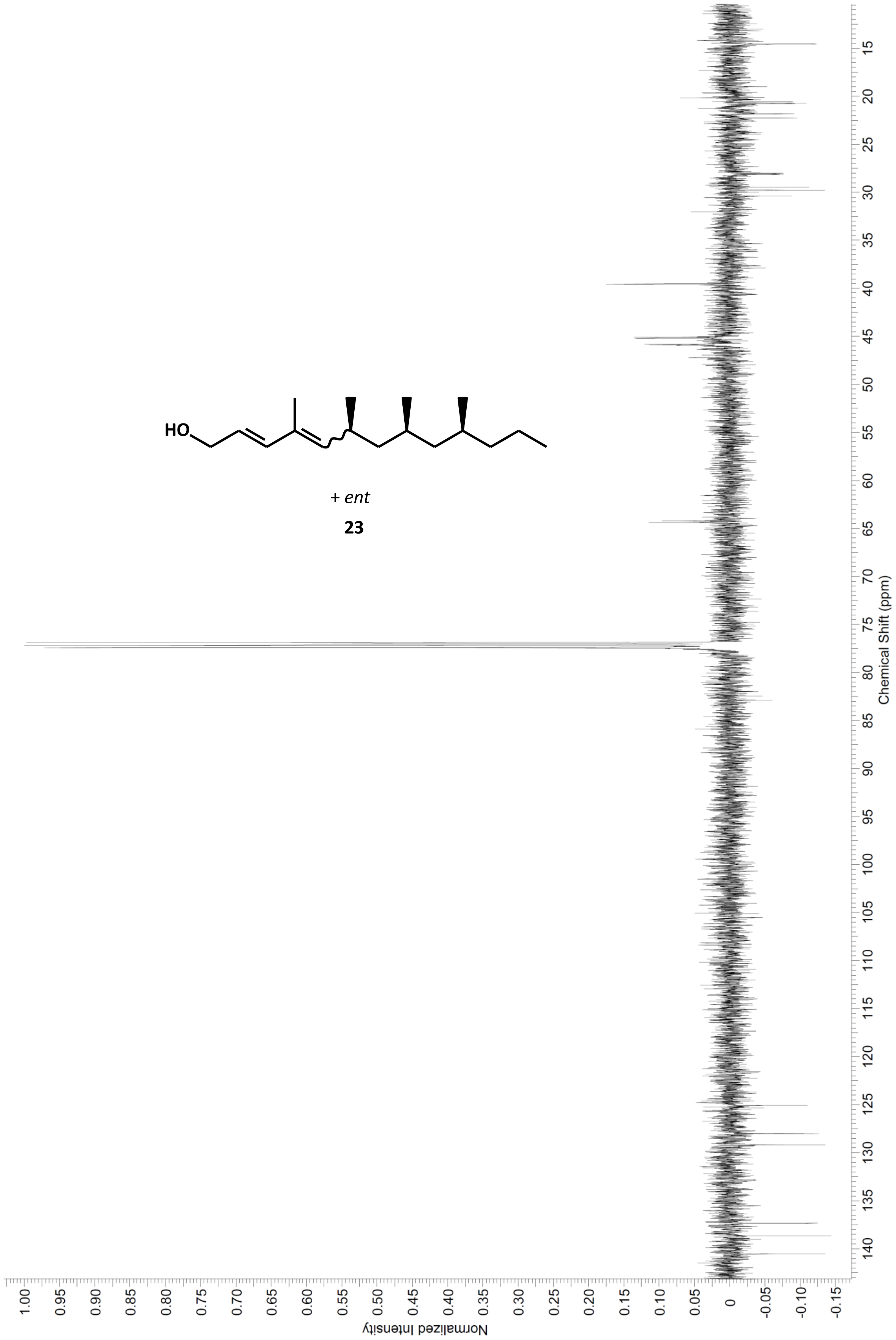

^{13}C-PENDANT-NMR spectrum (126 MHz, $CDCl_3$) of (2*E*,4*E*/*Z*)-*syn*,*syn*-4,6,8,10-tetramethyltrideca-2,4-dien-1-ol (**23**)

References

1. Haag, G.; Luu, B.; Hetru, C. *J. Chem. Soc. Perkin Trans*. I **1988**, 2353-2363.
2. Bartelt, R. J.; Dowd, P. F.; Plattner, R. D.; Weisleder, D. *J. Chem. Ecol*. **1990**, 16, 1015-1039.
3. Mori, K.; Kuwahara, S. *Tetrahedron* **1986**, *42*, 5545-5550.
4. Titze, J. Identifizierung und Synthese von Signalstoffen aus Insekten, PhD thesis, Hamburg. **2007**, 256 pp.
5. Markiewicz, J. T.; Schauer, D. J.; Löfstedt, J.; Corden, S. J.; Wiest, O.; Helquist, P. *J. Org. Chem*. **2010**, *75*, 2061-2064.
